# Effectiveness of decentralizing outpatient acute malnutrition treatment with community health workers and a simplified combined protocol: a cluster randomized controlled trial in emergency settings of Mali

**DOI:** 10.3389/fpubh.2024.1283148

**Published:** 2024-02-21

**Authors:** Noemí López-Ejeda, Pilar Charle-Cuéllar, Salimata Samake, Abdias Ogobara Dougnon, Luis Javier Sánchez-Martínez, Mahamadou N’tji Samake, Aliou Bagayoko, Magloire Bunkembo, Fanta Touré, Antonio Vargas, Saul Guerrero

**Affiliations:** ^1^EPINUT Research Group (ref. 920325), Unit of Physical Anthropology, Department of Biodiversity, Ecology and Evolution, Faculty of Biological Sciences, Complutense University of Madrid, Madrid, Spain; ^2^Nutrition and Health Department, Action Against Hunger, Madrid, Spain; ^3^Nutrition and Health Department, Action Against Hunger, Bamako, Mali; ^4^West and Central Africa Regional Office, Action Against Hunger, Dakar, Senegal; ^5^Nutrition Directorate of the General Directorate of Health and Public Hygiene, Ministry of Health, Bamako, Mali; ^6^Child Nutrition and Development Office, UNICEF, New York, NY, United States

**Keywords:** children, wasting, coverage, recovery, middle-upper arm circumference, ready-to-use therapeutic food

## Abstract

**Background:**

Outpatient treatment of acute malnutrition is usually centralized in health centers and separated into different programs according to case severity. This complicates case detection, care delivery, and supply chain management, making it difficult for families to access treatment. This study assessed the impact of treating severe and moderate cases in the same program using a simplified protocol and decentralizing treatment outside health centers through community health workers (CHWs).

**Methods:**

A three-armed cluster randomized controlled trial under a non-inferiority hypothesis was conducted in the Gao region of Mali involving 2,038 children between 6 and 59 months of age with non-complicated acute malnutrition. The control arm consisted of 549 children receiving standard treatment in health centers from nursing staff. The first intervention arm consisted of 800 children treated using the standard protocol with CHWs added as treatment providers. The second intervention arm consisted of 689 children treated by nurses and CHWs under the ComPAS simplified protocol, considering mid-upper arm circumference as the sole anthropometric criterion for admission and discharge and providing a fixed dose of therapeutic food for severe and moderate cases. Coverage was assessed through cross-sectional surveys using the sampling evaluation of access and coverage (SLEAC) methodology for a wide area involving several service delivery units.

**Results:**

The recovery rates were 76.3% in the control group, 81.8% in the group that included CHWs with the standard protocol, and 92.9% in the group that applied the simplified protocol, confirming non-inferiority and revealing a significant risk difference among the groups. No significant differences were found in the time to recovery (6 weeks) or in anthropometric gain, whereas the therapeutic food expenditure was significantly lower with the simplified combined program in severe cases (43 sachets fewer than the control). In moderate cases, an average of 35 sachets of therapeutic food were used. With the simplified protocol, the CHWs had 6% discharge errors compared with 19% with the standard protocol. The treatment coverage increased significantly with the simplified combined program (SAM +42.5%, MAM +13.8%).

**Implications:**

Implementing a simplified combined treatment program and adding CHWs as treatment providers can improve coverage while maintaining non-inferior effectiveness, reducing the expenditure on nutritional intrants, and ensuring the continuum of care for the most vulnerable children.

## 1 Introduction

Acute malnutrition or wasting is the most immediate, visible, and life-threatening form of undernutrition (1), affecting more than 45 million children under the age of five worldwide, 13.6 million of whom suffer from the most severe form (2). However, as these are prevalence estimates, the number of children affected annually is presumably higher (3). Several studies have demonstrated that the severity of wasting, and the presence of comorbidities are positively correlated with the risk of death if not treated properly. Compared to well-nourished children, all-cause mortality probability varies from up to 3.4 times in cases with moderate acute malnutrition (MAM) to up to 11.6 times in those with severe acute malnutrition (SAM) and up to 12.6 times when SAM is combined with other infectious diseases (4).

Treatment of acute malnutrition without medical complications is provided in outpatient facilities under the community management of acute malnutrition (CMAM) approach, which recommends two different treatment programs according to anthropometric severity at admission. These programs are run by different health staff in different locations and with different nutritional products managed by different United Nations (UN) agencies (5). Generally, SAM cases are treated by nurses in health centers with ready-to-use therapeutic food (RUTF) provided by the United Nations International Children’s Emergency Fund (UNICEF) and administered via a dose based on the child’s weight (6). However, MAM programs have varied considerably among countries as no international guidance existed until June 2023 (7). Children with MAM are treated by health staff or lay community health workers (CHWs) with fortified fortified blended flour, ready-to-use supplementary food (RUSF), or other lipid-based nutrient supplements generally provided by the World Food Program (8, 9).

Such differences complicate case detection, care delivery, and supply chain management and hinder families’ access to treatment (5). A review conducted in 21 low-and middle-income countries determined that less than 40% of SAM patients had access to the treatment they needed (5). The percentage was previously even lower for MAM. Given that its prevalence is markedly higher, and in the face of a lack of resources, treatment of SAM is often prioritized, resulting in MAM often being relegated to less effective nutritional counseling programs (8, 9).

In recent years, new simplified approaches for treating uncomplicated wasting have been proposed to increase the coverage and assure quality and effective treatment at a lower cost to countries (10). Recent reviews have shown that one simplified approach that has generated positive evidence is the decentralization of wasting treatment outside health centers by integrating it into the essential primary care package provided by CHWs at health posts closer to affected communities, an approach commonly referred to as integrated community case management (iCCM) (10, 11). However, some studies on low-literacy CHWs reported difficulties in managing the current nutritional treatment protocols, especially for SAM, which may limit its effectiveness (12–14). Similarly, other studies have shown that treatment outcomes of current protocols can be negatively affected if CHWs lack adequate supervision (15). Accordingly, a review conducted by UNICEF concluded that new studies analyzing the effects of combining treatment with CHWs and other simplified approaches are needed. Among the simplifications that can be tested are the use of middle-upper arm circumference (MUAC) as the sole criterion for admission and discharge (avoiding the use of the more complex weight-for-height (WHZ) indicator), the use of RUTF for both SAM and MAM, and modification of RUTF dosage (10).

Mali is one of the countries that has scaled up the treatment of SAM with CHWs. In 2015, the Health Ministry adapted its national primary healthcare policy to include acute malnutrition treatment in the activities that CHWs can provide outside health centers (16). Studies conducted in development contexts have shown positive results in terms of recovery, coverage, and cost-effectiveness (17–20). However, to date, no studies have evaluated this approach in emergency settings.

Over the last decade, Mali has been immersed in a complex humanitarian crisis due to armed conflict and extreme climatic conditions, with the northern region of Gao being one of the most affected (21). In 2019, the number of people internally displaced exceeded 199,000 most of whom were relocated to in the Mopti, Gao, Segou, Timbuktu, and Menaka regions, and approximately 2.2 million children were in need of humanitarian assistance (22). In that year, the overall prevalence of acute malnutrition in the country was 9.4%, with the prevalence in the Gao region reaching 11.6% (23). Owing to the COVID-19 pandemic, no prevalence surveys were conducted in the period 2020–2021, which coincides with the development period of the present study. The following survey, published after a dry period in 2022, showed an increase in the global prevalence of acute malnutrition of 10.8%, with a particularly alarming rise in the Gao region, which was the only region that exceeded the emergency threshold of 15%, reporting 16.1% (95% confidence interval (CI): 13.0–19.7), with SAM being 3.3% (95% CI: 2.2–4.9) (24).

Accordingly, the present study aimed to assess the impact on effectiveness and coverage of decentralizing acute malnutrition treatment with CHWs in the emergency settings of Mali, including both SAM and MAM cases within the same program. We applied a simplified protocol based on MUAC only for diagnosis and discharge, and a fixed dose of RUTF based on MUAC severity.

## 2 Population and methods

### 2.1 Study design and participants

A three-armed cluster randomized controlled trial was conducted from June 2020 to June 2021 in the Gao region of North Mali involving children aged 6–59 months with non-complicated acute malnutrition. The control group consisted of children treated by nurses in formal health centers using the standard treatment protocol approved by the Malian Ministry of Health (CMAM group) (25). The first intervention group applied the same treatment protocol but added lay CHWs as treatment providers in villages located at least 30 km from the referral health center (iCCM standard group). The second intervention group included both types of treatment providers (nurses and CHWs) but applied a combined, simplified protocol, known as the ComPAS protocol (26), which uses MUAC as the sole anthropometric measure for admission to and discharge from treatment and provides a fixed dose of RUTF to treat both severe and moderate cases (iCCM simplified group). None of the CHWs had previous experience treating malnutrition; they had only been involved in screening activities.

Table 1 summarizes the characteristics of the three study groups. It should be noted that patients with a weight at admission of less than 5 kg received half the RUTF dose proposed in the original ComPAS protocol. This is because, according to the Malian standard CMAM protocol, these children would have received a total quantity ranging between 105 and 130 g/day (1.25 and 1.5 sachets per day, depending on weight). Providing them the same dose as the rest of the children would have meant increasing their dose to 180 g/day (2 sachets per day). Therefore, we decided to provide a reduced amount of one sachet/day (92 g/day) to these children and tested this approaches’ effectiveness.

**Table 1:** Description of study procedures and treatment protocol applied in each study group.

		Control group (CMAM)	Intervention group 1 (iCCM Standard)	Intervention group 2 (iCCM Simplified)*
Treatment providers		Nurses at health centers	Nurses at health centers + CHWs in villages	Nurses at health centers + CHWs in villages
Follow-up frequency		Weekly	Weekly	Weekly
SAM	Admission criteria	Mild edema (+) or WHZ < −3 or MUAC < 115 mm	Mild edema (+) or WHZ < −3 or MUAC < 115 mm	Mild edema (+) or MUAC < 115 mm
	Product and dosage	RUTF/weight: 170 Kcal/kg/day	RUTF/weight: 170 Kcal/kg/day	RUTF fixed dosage: 2 sachets/day (=1,000 Kcal/day)**
	Recovery criteria	No edema and WHZ ≥ −1.5 or MUAC ≥ 125 mm for 2 consecutive follow-ups	No edema and WHZ ≥ −1.5 or MUAC ≥ 125 mm for 2 consecutive follow-ups	No edema and MUAC ≥ 125 mm for 2 consecutive follow-ups
MAM	Admission criteria	WHZ −3 to −2 or MUAC 115–125 mm	WHZ −3 to −2 or MUAC 115–125 mm	MUAC 115–125 mm
	Product and dosage	RUSF fixed dosage: 1 sachet/day (=537 Kcal/day)	RUSF fixed dosage: 1 sachet/day (=537 Kcal/day)	RUTF fixed dosage: 1 sachet/day (=500 Kcal/day)
	Recovery criteria	No edema and WHZ ≥ −1.5 or MUAC ≥ 125 mm for 2 consecutive follow-ups	No edema and WHZ ≥ −1.5 or MUAC ≥ 125 mm for 2 consecutive follow-ups	No edema and MUAC ≥ 125 mm for 2 consecutive follow-ups

CHWs, community health workers; CMAM, community management of acute malnutrition; iCCM, integrated community case management; MUAC, mid-upper arm circumference; RUSF, ready-to-use supplementary food; RUTF, ready-to-use therapeutic food; MAM, moderate acute malnutrition; SAM, severe acute malnutrition; WHZ, weight-for-height z-score.

*ComPAS simplified protocol proposed by Bailey et al. (26).

**ComPAS protocol modification: 1 sachet/day (=500 Kcal/day) if the weight at admission was <5 kg.

Patients with moderate and severe edema (++/+++) or severe medical complications and those who failed the appetite test (consumption of 30–45 g of RUTF within 15 min) were excluded from the study and referred for inpatient treatment. In addition, infectious comorbidities [e.g., malaria, diarrhea, and acute respiratory infection (ARI)] were recorded for those admitted. The primary outcome was recovery, which was established according to the criteria described in Table 1 for each study group. Secondary outcomes were the other treatment results: default when the child was absent for two consecutive follow-up visits; non-response when a weight loss of more than 5% in a visit or stagnation in anthropometric gain was recorded in two consecutive visits (considering weight or MUAC for standard protocol groups and only MUAC for the group applying the simplified protocol); referral when a medical complication or warning sign requiring inpatient treatment appeared; transfer when the family moved to another health area and the child was transferred between outpatient treatment programs; and death when the child died during treatment. Additionally, in cases discharged as recovered, the number of sachets of nutritional intrants was recorded; the time to recovery, also called length of stay (LOS), was calculated from the dates of admission and discharge, and the anthropometric gain was calculated according to standardized indicators for CMAM programs (27).

### 2.2 Sampling, randomization, and allocation

The sample size in each cluster was calculated using Sealed Envelope online software (28) built on the Blackwelder formula (29) based on a non-inferiority hypothesis for a primary binary outcome (recovery: yes/no), assuming 80% power and 95% sensitivity, with a recovery rate of 75% in the control group, as required by SPHERE standards (30), and a recovery rate of 85% in the intervention group, as most previous studies involving CHWs reported higher cure rates (11). The non-inferiority limit was set at 5% recovery (half of the expected difference between 75 and 85%), resulting in a required sample size of 87 children per cluster. The number of required clusters was calculated according to Hayes and Bennett’s formula for unmatched studies with a primary outcome reported as a proportion (31) and assuming a 0.05 intra-cluster correlation, as in the ComPAS study (26). The results indicated a minimum of six clusters (treatment sites) were required per study arm. After adding a 10% loss to follow-up, the estimated sample size was 576 SAM children per study arm and a total sample of 1,728 SAM children. Owing to initial economic limitations, SAM cases were prioritized to complement evidence from the ComPAS study (26), in which the majority of cases were moderate (76.2%). Therefore, sampling was initially designed to test the program’s effectiveness in the SAM cases. However, during this study, it was possible to collect data from MAM cases that were included without prior sample calculations.

Although blinding was not possible for the treatment providers, they could not access the overall outcomes during the study period. Of all possible health centers in the Gao region, those that reported the highest number of cases treated in the same period of the previous year and those that were at least 5 km away from each other to avoid patient crossover were selected. The final treatment sites (clusters) are described in the supporting information (Supplementary Table S1, Supplementary Figure S1).

The unit of randomization was the health center. Given that CHWs are assigned to a specific health center to which they report their activities and are responsible for supervising, they should apply the same treatment protocol as that in their reference center. The study coordinator in the field performed the randomization using an Excel spreadsheet with a 2:1:1 allocation ratio to avoid sample imbalance; for each health center assigned to the intervention group, two health centers were assigned to the control group. This approach was based on previous studies on iCCM interventions conducted in Mali, where CHWs were found to treat more SAM cases than health centers causing a markedly lower number of children in the control group without CHWs (17). All children attending the treatment sites who complied with the inclusion criteria were invited to participate in this study.

### 2.3 Coverage and socioeconomic assessments

Baseline and endline coverages were assessed using large-scale cross-sectional surveys of a representative sample of households in each study arm. The sample size was calculated based on the lists of villages by health area in each arm, population figures provided by the Gao District medical authorities, and wasting prevalence provided by the results of the last Standardized Monitoring and Assessment of Relief and Transitions (SMART) survey. In total, 4,829 children aged 6–59 months from 63 villages were screened in the initial survey and 6,619 children from 65 villages were screened in the final survey. Coverage was estimated following the recommendations for wide areas involving several service delivery units of the Simplified Lot Quality Assurance Sampling Evaluation of Access and Coverage (SLEAC) Technical Reference (32) designed explicitly for CMAM programs. The survey included case identification by edema, WHZ, and MUAC and applied the cutoff points accepted in the country’s standard treatment protocol described for the control group in Table 1. An initial assessment was performed from February to March 2020 prior to the start of enrollment. The final coverage assessment was conducted employing the same methodology in the same villages in May 2021. Twelve independent surveyors who were trained for 5 days performed the assessments. For more details on the methodology, the complete coverage reports are available as Supplementary material.

To better contextualize the results, a socioeconomic questionnaire was administered to a random sample of participants (those cases that coincided at the treatment site with the supervisor on their visit). Results were collected from 30 of the 32 treatment sites (two health centers, one in the control group and another in the simplified iCCM group, were missing). The questionnaire collected demographic, livelihood, and food security variables, similar to those gathered in the Demographic Health Surveys (DHS) (33). In addition, dietary diversity was assessed according to the Food Consumption Score developed by the World Food Program (34) based on the frequency of consumption of nine food groups. Finally, the questionnaire asked about the main barriers to treatment access, as identified by Rogers et al. (5).

### 2.4 Implementation and ethical considerations

Cascade training was developed before the children’s enrollment to ensure the quality of care. In the first stage, the head of the health district from the Ministry of Health and the study coordinator from Action Against Hunger received training for 3 days. In the second stage, they were responsible for the 21-day training of the nurses and CHWs, including the learning modules of all primary healthcare activities included in the country’s iCCM package: promotion of good hygiene and feeding practices, preventive actions (antenatal consultation, family planning, and vaccination), danger sign identification, referral procedures, and curative activities for malaria, diarrhea, pneumonia, and acute malnutrition. An additional day of specific training in management was provided to nurses and CHWs assigned to the simplified protocol group. In the third stage, all CHWs completed an internship at their reference health center for 3 months. After the study setup, the CHWs received monthly joint supervision from the Action Against Hunger staff and health center nurses.

The implementation of the study was assessed every 3 months by a committee formed by the head of health district team and local stakeholders (Association Amis de Gao). The study was additionally assessed by a national committee comprising specialized staff from the Nutrition Directorate of the Ministry of Health, the National Public Health Institute (*Institut National de Santé Publique*, INSP) and the Action Against Hunger coordinator.

For children’s inclusion, treatment providers obtained informed consent in the local language from all parents or caregivers. Those who refused to participate received treatment as in any other case; however, their data were not included in the study records. This study was approved by the Ethics Committee of the INSP, the reference agency of the Ministry of Health of the government of Mali (decision no. 35/2029/CE-EX-INRSP), and the Ethics Committee of the Hospital Clínico San Carlos, the reference organism for human studies of the Complutense University of Madrid, Spain (favorable report C.I. 19/363-R-X-BC). The study protocol was registered in the ISRCTN under reference ISRCTN-60973756, and reporting followed the Consolidated Standards of Reporting Trials (CONSORT) guidelines (35) and its extension to cluster randomized trials (36) and non-inferiority randomized trials (37) (Supplementary Tables S2, S3). No specifications were reported for multi-arm randomized trials because, according to the corresponding CONSORT extension (38), no special adjustments to sample size calculation or data analysis are necessary when comparisons between arms are made on a pairwise basis.

### 2.5 Data collection and analysis

Study supervisors collected treatment data directly from the patient record book at the treatment site via tablets using the Kobo Toolbox app (39). Socioeconomic data were recorded using the Kobo application through direct interviews with mothers or caregivers at the treatment site. Initially, a monthly collection visit was planned; however, the worsening in security situation prevented the teams from traveling at various times during the study, making data collection difficult. Finally, eight of the 12 planned visits were made, five of which were consecutive during the first month of the study. Several data collectors worked simultaneously; therefore, all sites were visited on the same dates.

Cases that had been registered as recovered but did not meet the anthropometric criteria were classified as “early discharge.” The country protocol indicates that children admitted by MUAC should be recovered by MUAC, those admitted by WHZ should be recovered by WHZ, and those admitted simultaneously meeting both criteria should be recovered by the MUAC or WHZ criteria. However, data collection limitations resulted in a final database with missing data or potential errors that could not be confirmed. Patients without anthropometric information at discharge were excluded. In all groups, those that did not have anthropometry recorded at admission were considered recovered if either of the indicators at discharge had met the corresponding recovery criteria and as “early discharge” if both indicators had not met the criteria. The same criterion was applied for cases with implausible anthropometry at admission but who remained in treatment for several weeks; for these cases, the anthropometric data were considered to result from transcription errors. Accordingly, their data were excluded from the analysis of anthropometry at admission, weight, and MUAC gain. In the control and standard iCCM groups, those cases that did not have either of the two anthropometric variables recorded at discharge but met the recovery criterion with the recorded variable according to the anthropometry at admission were considered recovered and those that did not considered “early discharge.” In the simplified group, all children whose MUAC at discharge was <125 mm were considered to have had “early discharge.”

Statistical analyses were performed using R Studio. Baseline characteristics were compared using Fisher’s exact test and the Mann–Whitney U test. Statistical power to assess non-inferiority for the primary outcome (recovery) was calculated using the epi.ssninfb function in the epiR package (40). The adjusted risk differences between pairs of protocols were estimated using random effects logistic models through the glmer function of the lme4 package, adjusting for clusters as random effects (41). The CIs of the estimates were calculated using bootstrapping and performing 200 resamplings, as proposed by Kleinman and Norton (42). Non-inferiority was declared if the upper bound of the 95% CI for the difference in recovery was less than 5%. Mixed-effects models were used to compare quantitative variables, treating the clusters as random effects in the lme function of the nlme package (43). The Cochran–Mantel–Haenszel test was used to compare the final coverage between the study groups, adjusted for baseline coverage. For secondary outcomes and variables, a bilateral hypothesis (2-sided) was considered with a confidence limit of 95% (*p* < 0.05).

## 3 Results

**Figure 1:**
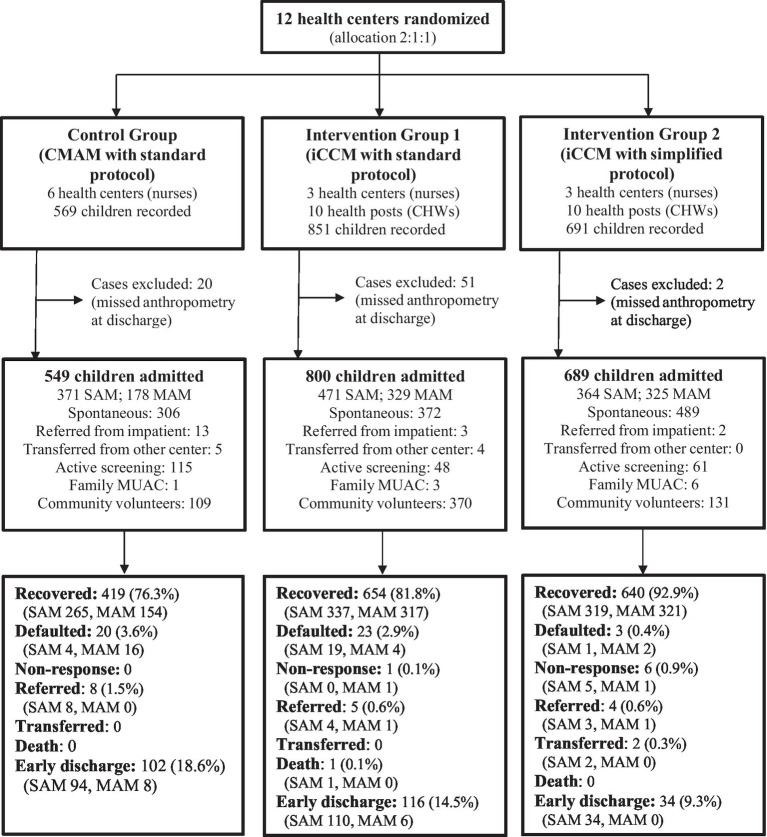
Study participants flow diagram. CHWs, community health workers; CMAM, community management of acute malnutrition; iCCM, integrated community case management; MAM, moderate acute malnutrition; MUAC, mid-upper arm circumference; SAM, severe acute malnutrition.

The sample size in each group was as follows: 549 children in the control group (371 SAM and 178 MAM), 800 in the iCCM group using the standard protocol (471 SAM and 329 MAM), and 689 in the iCCM group using the simplified protocol (364 SAM and 325 MAM). Owing to security reasons in the field, it was not possible to reach the sample size proposed for SAM cases, and only 70% of the calculated sample (1,206 children out of 1,728) was obtained. Figure 1 presents a flow diagram of participant selection. The statistical power achieved for the primary outcome comparison exceeded 90% for all pairs of comparisons, except for the control vs. iCCM standard for the SAM subgroup (49.3%) (Supplementary Table S4).

Table 2 shows the socioeconomic results for the entire sample (specific results for SAM and MAM are available in the supporting information file, Supplementary Tables S5, S6). Although significant differences were found for some items, none of the groups showed a worse condition in all aspects evaluated. The control group generally had more cases of housing construction with access to electricity and fewer cases of food insecurity than the other two groups (lack of food experienced three or more times in the previous weeks). Regarding access to health services, the iCCM standard group reported more difficulties, and the iCCM simplified group had a greater distance to the treatment centers.

**Table 2:** Socioeconomic characteristics of acutely malnourished children at admission, compared by study group.

Total sample		Control CMAM^a^ (*n* = 202)		iCCM standard^b^ (*n* = 326)		iCCM simplified^c^ (*n* = 148)		a vs. b *p*-value	a vs. c *p*-value	b vs. c *p*-value
		*N*	Mean (SD) or % (*n*)	*N*	Mean (SD) or % (*n*)	*N*	Mean (SD) or % (*n*)			
Demograph	Cohabiting people	202	7.2 (2.5)	326	8.9 (4.0)	148	7.5 (2.9)	**<0.001**	0.500	**<0.001**
	Children under 5 cohabiting	196	1.4 (1.3)	318	1.5 (1.7)	140	1.1 (1.4)	0.286	0.182	**0.016**
	Years of education of primary caregiver	163	0.19 (0.5)	301	0.50 (1.7)	139	0.30 (0.6)	**0.033**	0.506	0.192
Livelihoods	Type of housing	201		318		146				
	In property		88.1% (177)		96.9% (308)		92.5% (135)	**<0.001**	0.244	0.122
	For rent		10.5% (20)		1.3% (4)		4.1% (6)	**<0.001**	0.097	0.105
	On loan		1.5% (3)		1.9% (6)		3.4% (5)	0.999	0.999	0.999
	With access to safe water	202	12.4% (24)	326	10.1% (32)	148	10.8% (15)	0.999	0.999	0.999
	With safe sanitation	192	19.8% (37)	224	1.3% (3)	116	4.3% (5)	**<0.001**	**<0.001**	0.181
	With electricity	202	85.2% (172)	326	23.0% (75)	148	15.5% (22)	**<0.001**	**<0.001**	0.082
	With arable land	201	13.4% (26)	326	89.0% (290)	147	86.4% (127)	**<0.001**	**<0.001**	0.520
	With livestock	197	41.1% (81)	326	64.7% (211)	146	36.3% (44)	**<0.001**	0.430	**<0.001**
	With construction floor	198	36.4% (72)	325	4.3% (13)	144	4.2% (6)	**<0.001**	**<0.001**	0.999
	With construction roof	197	29.4% (45)	325	6.2% (19)	147	4.1% (6)	**<0.001**	**<0.001**	0.490
Food security	Number of meals/day	201	2.8 (0.35)	325	2.8 (0.43)	148	2.5 (0.58)	0.440	**<0.001**	**<0.001**
	Lack of food last 4 weeks	175		325		148				
	Never		12.6% (21)		9.9% (31)		18.9% (27)	0.432	0.313	**0.028**
	Rarely		74.9% (131)		63.4% (206)		58.1% (86)	**0.024**	**0.006**	0.320
	3–10 times		11.4% (19)		24.0% (78)		21.6% (31)	**0.003**	**0.039**	0.652
	More than 10 times		1.1% (2)		2.8% (9)		1.4% (2)	0.999	0.999	0.999
	Food consumption score	202	61.0 (16.6)	326	52.1 (21.3)	148	43.7 (18.6)	**<0.001**	**<0.001**	**<0.001**
	Poor diet		0.5% (1)		13.5% (43)		6.8% (9)	**<0.001**	0.005	**0.047**
	Limited diet		6.9% (13)		8.9% (28)		28.4% (41)	0.520	**<0.001**	**<0.001**
	Acceptable diet		92.6% (187)		77.6% (253)		64.9% (96)	**<0.001**	**<0.001**	**0.005**
Heath care access	Behavior if child is sick	201		326		145				
	Health post or CHW		79.1% (159)		88.3% (288)		86.9% (126)	**0.018**	0.166	0.771
	Traditional medicine		16.9% (33)		11.7% (37)		6.9% (9)	0.230	**0.028**	0.230
	Self medication		4.0% (8)		0.0 (0)		6.2% (9)	**0.002**	0.488	**<0.001**
	With difficulty of access	199	11.6% (22)	325	25.5% (83)	147	12.9% (18)	**<0.001**	0.827	**0.006**
	Time to get to treatment	172		321		146				
	30 min or less		77.3% (133)		75.7% (243)		48.0% (70)	0.770	**<0.001**	**<0.001**
	Up to 1.5 h		11.1% (18)		15.0% (46)		34.9% (47)	0.290	**<0.001**	**<0.001**
	More than 2 h		11.6% (19)		9.4% (29)		17.1% (24)	0.520	0.430	0.071

CHW, Community Health Worker; CMAM, Community management of acute malnutrition; iCCM, integrated Community Case Management; SD, standard deviation. In bold the significant results.^a,^^b,^^c^ They are identifiers to understand the last three columns that compare two by two the study groups (group a, b and c).

Table 3 shows the admission characteristics for the entire sample compared by group (the results for the SAM and MAM cases are available in the supporting information file, Supplementary Table S7). There was a slightly higher proportion of girls in all groups. The median age differed by only 2 months among the groups, but the iCCM group had a higher proportion of younger children. The iCCM simplified group showed greater anthropometric severity at admission with lower WHZ and MUAC values than the other two groups for the total sample and within the SAM group. Similarly, in the SAM cases, the iCCM standard group showed lower values than the control group for all indicators. Regarding other diseases assessed at admission, malaria and diarrhea had a low prevalence in all groups. Acute respiratory infections were more prevalent in the control and iCCM standard groups, especially among MAM cases, affecting a quarter of the sample. The prevalence was significantly lower (<5%) in the simplified protocol group.

**Table 3:** Admission characteristics compared between study groups for the total sample.

Total sample	Control CMAM^a^ *N* = 549	iCCM standard^b^ *N* = 800	iCCM simplified^c^ *N* = 689	a vs. b *p*-value	a vs. c *p*-value	b vs. c *p*-value
Sex male, % (*n*)	43.4 (238)	47.6 (381)	49.2 (339)	0.133	**0.045**	0.567
**Age (months)**						
Median (IQR)	14.0 [10.0–20.0]	12.0 [9.0–20.0]	12.0 [9.0–18.0]	**0.030**	**<0.001**	**0.002**
6–12, % (*n*)	43.5 (239)	51.9 (415)	55.2 (380)	**0.005**	**<0.001**	0.082
12–24, % (*n*)	45.0 (247)	40.1 (321)	39.6 (273)			
> 24, % (*n*)	11.5 (63)	8.0 (64)	5.2 (36)			
Anthropometry	Median [IQR]	Median [IQR]	Median [IQR]			
Weight (kg)	7.0 [6.0–8.1]	6.9 [6.0–7.9]	6.2 [5.4–7.0]	0.113	**<0.001**	**<0.001**
WHZ (z-score)	−2.99 [−3.59 to −2.26]	−2.82 [−3.48 to −2.09]	−3.09 [−4.69 to −2.39]	**0.026**	**0.007**	**<0.001**
MUAC (mm)	116.0 [111.0–120.0]	115.0 [111.0–120.0]	114.0 [110.0–120.0]	0.228	0.071	0.420
Edema, % (*n*)	0 (0)	0 (0)	0.3 (1)	–	0.497	0.428
Other diseases	% (n)	% (n)	% (n)			
Malaria	3.3 (18)	3.6 (29)	1.6 (11)	0.733	0.059	**0.016**
Diarrhea	1.8 (9)	0.9 (7)	2.2 (13)	0.182	0.669	0.067
ARI	16.1 (79)	16.7 (114)	3.6 (213)	0.767	**<0.001**	**<0.001**

ARI, acute respiratory infection; CMAM, community management of acute malnutrition; iCCM, integrated community case management; MUAC, mid-upper arm circumference; WHZ, weight-for-height z-score. The significant results are in bold.

^a,^^b,^^c^ They are identifiers to understand the last three columns that compare two by two the study groups (group a, b and c).

**Figure 2:**
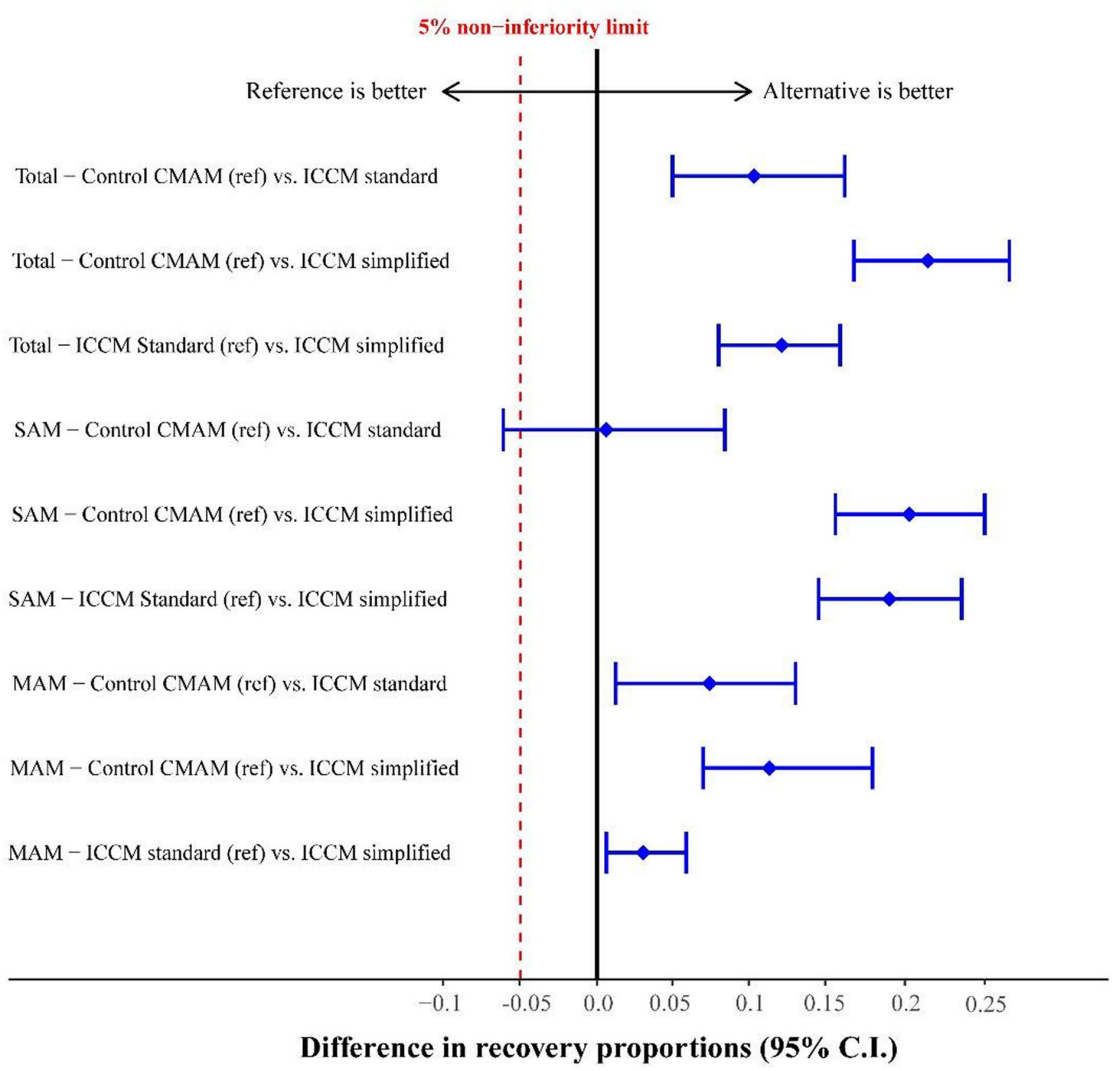
Risk difference in the recovery proportion of study groups adjusted by clusters for the total sample and by severe (SAM) and moderate (MAM) cases.

Table 4 shows the results of the program’s effectiveness. The iCCM simplified group reached the highest recovery proportion (93%), followed by the iCCM standard (82%), and the control group (76%), with a significant risk difference between some groups after adjusting for cluster, sex, age, and MUAC severity at admission. Recovery in SAM cases was lower than that in MAM cases owing to a markedly higher proportion of early discharge. The default cases were below 4% for the total sample and were slightly higher for MAM cases in the control group (9%). Notably, only one death was recorded during the study. Figure 2 shows the non-inferiority of the intervention groups, verifying that in no case did the 95% CI of the difference in proportions exceed the preset value of 5%. Supplementary Table S8 provides the intra-cluster correlation coefficients for recovery for each paired comparison. In addition, Supplementary Table S9 and Supplementary Figure S2 provide the results of the outcomes contemplated in the SPHERE standards (recovered, defaulted, and death), which can be considered the per-protocol analysis. The proportion of recovered children was high in all groups (>95%), with non-inferiority in the intervention groups compared to the control group for the total sample and the SAM or MAM subgroups.

**Table 4:** Treatment outcomes compared by study group for the total sample and severe and moderate cases.

	Control CMAM^a^ % (*n*)	iCCM standard^b^ % (*n*)	iCCM simplified^c^ % (*n*)	b vs. a Risk difference [95% C.I.]*	*p*-value	c vs. a Risk difference [95% C.I.]*	*p*-value	c vs. b Risk difference [95% C.I.]*	*p*-value
**Total sample**	*N* = 549	*N* = 800	*N* = 689						
Recovered	76.3 (419)	81.8 (654)	92.9 (640)	**0.10 [0.04–0.15]**	**<0.001**	**0.21 [0.16–0.26]**	**<0.001**	**0.12 [0.09–0.15]**	**<0.001**
Defaulted	3.6 (20)	2.9 (23)	0.4 (3)	−0.01 [−0.03–0.01]	0.277	–	–	–	–
Non-response	0 (0)	0.1 (1)	0.9 (6)	–	–	–	–	–	–
Referenced	1.5 (8)	0.6 (5)	0.6 (4)	–	–	–	–	–	–
Transferred	0 (0)	0 (0)	0.3 (2)	–	–	–	–	–	–
Death	0 (0)	0.1 (1)	0 (0)	–	–	–	–	–	–
Early discharge	18.6 (102)	14.5 (116)	4.9 (34)	**−0.08 [−0.14 to −0.02]**	**0.002**	**−0.17 [−0.22 to −0.12]**	**<0.001**	**−0.10 [−0.13 to −0.08]**	**<0.001**
**SAM**	*N* = 371	*N* = 471	*N* = 364						
Recovered	71.4 (265)	71.5 (337)	87.6 (319)	0.02 [−0.06–0.10]	0.338	**0.23 [0.17–0.29]**	**<0.001**	**0.20 [0.14–0.25]**	**<0.001**
Defaulted	1.1 (4)	4.0 (19)	0.3 (1)	–	–	–	–	–	–
Non-response	0 (0)	0 (0)	1.4 (5)	–	–	–	–	–	–
Referenced	2.2 (8)	0.8 (4)	0.8 (3)	–	–	–	–	–	–
Transferred	0 (0)	0 (0)	0.5 (2)	–	–	–	–	–	–
Death	0 (0)	0.2 (1)	0 (0)	–	–	–	–	–	–
Early discharge	25.3 (94)	23.4 (110)	9.3 (34)	−0.04 [−0.12 to 0.03]	0.126	**−0.22 [−0.27 to −0.17]**	**<0.001**	**-0.17 [−0.21 to −0.13]**	**<0.001**
**MAM**	*N* = 178	*N* = 329	*N* = 325						
Recovered	86.5 (154)	96.4 (317)	98.8 (321)	**0.07 [0.02–0.13]**	**0.006**	**0.12 [0.06–0.18]**	**<0.001**	**0.03 [0.01–0.06]**	**0.010**
Defaulted	9.0 (16)	1.2 (4)	0.6 (2)	–	–	–	–	–	–
Non-response	0 (0)	0.3 (1)	0.3 (1)	–	–	–	–	–	–
Referenced	0 (0)	0.3 (1)	0.3 (1)	–	–	–	–	–	–
Transferred	–	–	–	–	–	–	–	–	–
Death	–	–	–	–	–	–	–	–	–
Early discharge	4.5 (8)	1.8 (6)	0 (0)	–	–	–	–	–	–

*Adjusted for cluster, sex, age, and anthropometry at admission only performed for recovered, defaulted, and early discharge because the remainder of the outcomes registered less than 5% of cases in the 3 study groups. The significant results are in bold. C.I., confidence interval; CMAM, community management of acute malnutrition model; iCCM, integrated community case management; SAM, severe acute malnutrition; MAM, moderate acute malnutrition.

^a,^^b,^^c^ They are identifiers to understand the last three columns that compare two by two the study groups (group a, b and c).

Children who recovered in the control group recorded significantly more missing follow-up visits (6.9%) compared to the standard iCCM group (3.7%, *p* = 0.008) and the simplified iCCM group (1.7%, *p* < 0.001). The difference between the iCCM groups was also statistically significant (*p* = 0.025). Table 5 shows the results regarding the time to recovery and nutritional product expenditure reported for the recovered cases. The average LOS was 6 weeks in the three groups for the total sample and the SAM subgroup, although a higher dispersion in the data was found in the iCCM groups. In children with MAM, cases in the simplified group required, on average, 6 more days for recovery. In the SAM subgroup, children treated with the simplified protocol had the lowest therapeutic food expenditure (on average, 18 sachets fewer than that in the other iCCM group and 43 sachets fewer than that in the control group). In the MAM group, children treated with RUTF in the simplified protocol required 35 sachets on average. In the two groups with the standard protocol, the group that included CHWs as treatment providers recorded lower RUSF expenditures than the control group (13 fewer sachets on average).

**Table 5:** Time to recovery, amount of ready-to use food expenditure used for treatment, and anthropometric gain of recovered children compared by study group.

	Control CMAM^a^ Median [IQR]	iCCM standard^b^ Median [IQR]	iCCM simplified^c^ Median [IQR]	a vs. b *p*-value	a vs. c *p*-value	b vs. c *p*-value
**Whole program**						
LOS (days)	42.0 [35.0–50.0]	42.0 [32.75–69.0]	43.0 [30.5–67.5]	0.329	0.342	0.780
RUTF sachets	120.0 [100.0–140.0]	95.0 [75.0–120.0]	49.0 [35.0–77.0]	**0.020**	**<0.001**	**<0.001**
RUSF sachets	47.5 [32.0–56.0]	35.0 [28.0–42.0]	–	0.462	–	–
Weight gain (g/kg/day)	4.66 [3.17–6.71]	3.77 [2.02–6.66]	4.76 [2.82–7.14]	0.517	0.816	0.407
MUAC gain (mm/day)	0.34 [0.21–0.55]	0.23 [0.12–0.39]	0.25 [0.13–0.39]	0.632	0.337	0.552
**SAM**						
LOS (days)	42.0 [34.75–49.0]	42.0 [31.25–63.0]	42.0 [35.0–63.0]	0.385	0.405	0.733
RUTF sachets	120.0 [100.0–140.0]	95.0 [75.0–120.0]	76.5 [70.0–91.0]	**0.020**	**<0.001**	**0.002**
Weight gain (g/kg/day)	5.95 [4.65–8.03]	5.18 [2.83–7.04]	5.89 [3.49–7.47]	0.478	0.918	0.415
MUAC gain (mm/day)	0.31 [0.21–0.52]	0.32 [0.21–0.49]	0.37 [0.25–0.47]	0.606	0.614	0.834
**MAM**						
LOS (days)	42.0 [35.0–50.0]	42.0 [33.25–77.0]	48.0 [28.0–70.0]	0.202	0.340	0.911
RUTF sachets	–	–	35.0 [28.0–42.0]	–	–	–
RUSF sachets	47.5 [32.0–56.0]	35.0 [28.0–42.0]	–	0.462	–	–
Weight gain (g/kg/day)	3.53 [2.56–4.43]	2.63 [1.35–4.85]	3.16 [1.59–4.63]	0.822	0.480	0.442
MUAC gain (mm/day)	0.40 [0.21–0.55]	0.14 [0.08–0.27]	0.14 [0.08–0.25]	0.323	0.051	0.198

CMAM, community management of acute malnutrition; iCCM, integrated community case management; LOS, length of stay; MUAC, mid-upper arm circumference; RUTF, ready-to-use therapeutic food; RUSF, ready-to-use supplementary food. Significant results are in bold.

^a,^^b,^^c^ They are identifiers to understand the last three columns that compare two by two the study groups (group a, b and c).

In addition, treatment outcomes were compared by matching the anthropometric entry and discharge criteria to assess the effects of the nutritional product and dosage modification. Accordingly, only those cases admitted with an MUAC < 125 mm in the control and the iCCM standard groups were included, eliminating those cases with MUAC > 125 mm but WHZ < −2 on admission, and considering only MUAC ≥ 125 mm as the recovery criterion. Early discharge cases (MUAC < 125 mm) were also excluded. In total, 40.4% (222) of the cases in the control group, 17.0% (136) in the standard ICCM group, and 4.9% (33) in the simplified iCCM group were excluded from the analysis (Supplementary Table S10). The results in this subsample show that the proportion of recovered patients in the simplified group was 97.7%, compared to 95.5% in the iCCM standard group and 91.4% in the control group indicating a significant risk difference among groups. In addition, Supplementary Table S11 provides the other treatment results for this subgroup, which were admitted and discharged by the MUAC. When considering the entire sample, children with SAM with the simplified protocol expended significantly less RUTF with similar anthropometric gains; 43 and 23 sachets fewer than the control group and iCCM standard groups, respectively.

Supplementary Table S12 presents the treatment results for the 176 children weighing less than 5 kg who were admitted. The proportion of recovered patients in the simplified protocol group was 91.3%, compared to 76.0 and 69.5% in the control and iCCM standard groups, respectively, although the magnitude of these differences was only significant between the iCCM groups. This was due to the differences in early discharge cases. The time to recovery in this low-weight group was longer than that of the total sample in the iCCM standard, which reached 60 days on average. The simplified protocol group had a significantly lower therapeutic food expenditure, and anthropometric gain did not differ between the groups.

Table 6 shows the treatment outcomes obtained by CHWs and nurses within each iCCM group. Nurses in the standard iCCM group had significantly higher recovery rates in the SAM and MAM subgroups. In contrast, no significant differences were found between the two treatment providers in the simplified protocol group. The difference in recovery rate was much more pronounced in the SAM group, where the CHWs achieved a 32% lower recovery than nurses compared to a 10% lower recovery rate in the simplified protocol group. Other treatment results (time to recovery, anthropometric gain, and product expenditure) compared by provider are provided in the supporting information (Supplementary Table S13). In SAM cases treated under the simplified protocol, those treated by CHWs required one additional week to recover (48 vs. 41 days) but registered lower RUTF expenditures and lower anthropometric gain than those treated by nurses. There were no registered MAM cases treated by nurses in the simplified protocol group; therefore, a comparison with CHWs could not be made.

**Table 6:** Treatment outcomes compared by provider in the standard and simplified protocol groups.

	iCCM standard				iCCM simplified			
	CHWs % (*n*)	Nurses % (*n*)	Risk difference [95% C.I.]*	*p*-value	CHWs % (*n*)	Nurses % (*n*)	Risk difference [95% C.I.]*	*p*-value
**Total sample**	*N* = 579	*N* = 221			*N* = 560	*N* = 214		
Recovered	76.2 (441)	96.4 (213)	**0.20 [0.16–0.24]**	**<0.001**	92.1 (516)	96.1 (124)	0.01 [−0.04–0.07]	0.312
Defaulted	4.0 (23)	0 (0)	–	–	0.5 (3)	0 (0)	–	–
Non-response	0.2 (1)	0 (0)	–	–	1.1 (6)	0 (0)	–	–
Referenced	0.7 (4)	0.5 (1)	–	–	0.4 (2)	1.6 (2)	–	–
Transferred	0 (0)	0 (0)	–	–	0.4 (2)	0 (0)	–	–
Death	0.2 (1)	0 (0)	–	–	–	–	–	–
Early discharge	18.8 (109)	3.2 (7)	–	–	5.5 (31)	2.3 (3)	–	–
**SAM**	*N* = 336	*N* = 135			*N* = 235	*N* = 129		
Recovered	62.5 (210)	94.1 (127)	**0.35 [0.28–0.41]**	**<0.001**	83.0 (195)	93.1 (124)	0.02 [−0.08–0.11]	0.395
Defaulted	5.7 (19)	0 (0)	–	–	0.4 (1)	0 (0)	–	–
Non-response	0 (0)	0 (0)	–	–	2.1 (5)	0 (0)	–	–
Referenced	0.9 (3)	0.7 (1)	–	–	0.4 (1)	1.6 (2)	–	–
Transferred	0 (0)	0 (0)	–	–	0.9 (2)	0 (0)	–	–
Death	0.3 (1)	0 (0)	–	–	0 (0)	0 (0)	–	–
Early discharge	30.7 (103)	5.2 (7)	–	–	13.2 (31)	2.3 (3)	–	–
**MAM**	*N* = 231	*N* = 86			*N* = 325			
Recovered	95.1 (231)	100.0 (86)	**0.05 [0.02–0.05]**	**0.001**	98.8 (321)	–	–	–
Defaulted	1.6 (4)	0 (0)	–	–	0.6 (2)	–	–	–
Non-response	0.4 (1)	0 (0)	–	–	0.3 (1)	–	–	–
Referenced	0.4 (1)	0 (0)	–	–	0.3 (1)	–	–	–
Transferred	0 (0)	0 (0)	–	–	0 (0)	–	–	–
Death	0 (0)	0 (0)	–	–	0 (0)	–	–	–
Early discharge	2.5 (6)	0 (0)	–	–	0 (0)	–	–	–

*Adjusted for cluster, sex, age, and anthropometry at admission only performed for recovered and early discharge because the remainder of the outcomes registered less than 5% of cases in the 3 study groups. CHWs, community health workers; CI, confidence interval; CMAM, community management of acute malnutrition; iCCM, integrated community case management; LOS, length of stay; MUAC, mid-upper arm circumference; RUTF, ready-to-use therapeutic food; RUSF, ready-to-use supplementary food; MAM, moderate acute malnutrition; SAM, severe acute malnutrition. Significant results are in bold.

**Figure 3:**
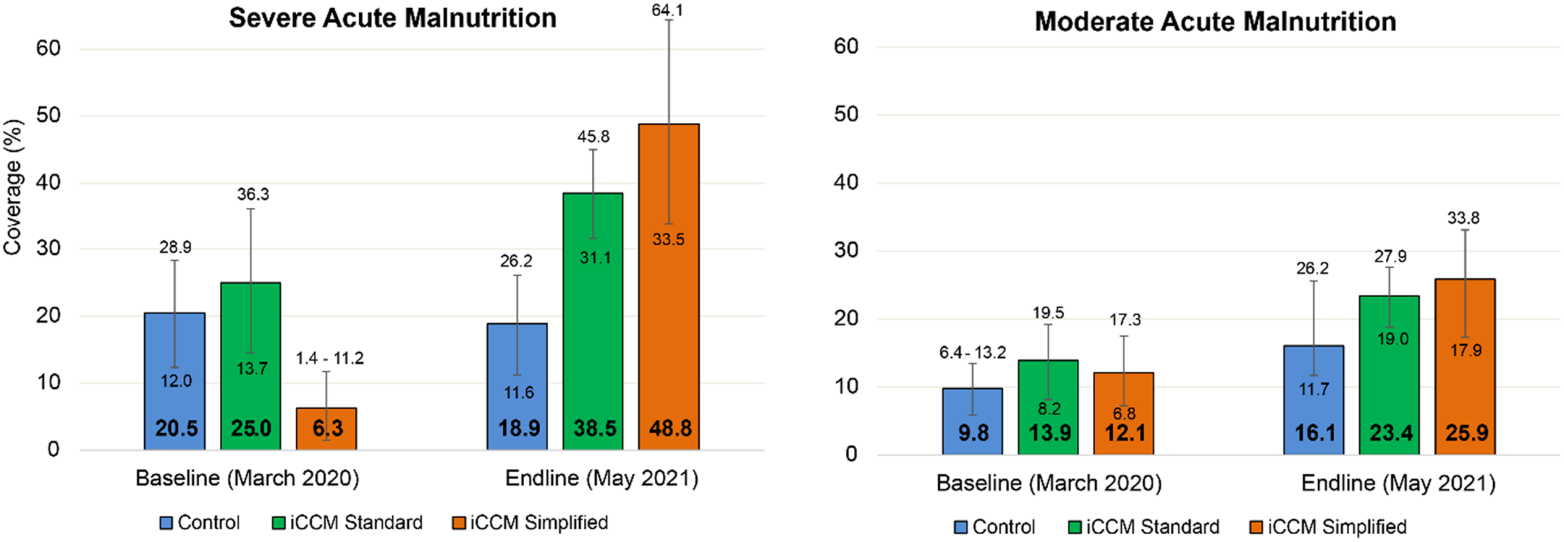
Acute malnutrition coverage with 95% confidence interval at baseline and endline by study group.

Figure 3 shows the results of the treatment coverage analysis. In the SAM sample, coverage increased by +42.5% in the simplified iCCM group, whereas in the other groups, the CIs overlapped between the beginning and end of the study. The comparison of the endline coverage adjusted for baseline coverage (Cochran–Mantel–Haenszel analysis) showed significant differences between the control group and the standard iCCM group (18.9% vs. 38.5%, *p* = 0.002) but not between the other groups. In MAM cases, coverage was increased in both iCCM groups (iCCM standard +9.5%, and iCCM simplified +13.8%), and the comparison of endline coverage adjusted to baseline coverage was significant between the control (16.1%) and both iCCM groups (23.4%, *p* = 0.013 for iCCM standard and 25.9%, *p* = 0.040 for iCCM simplified), but not between the iCCM groups (23.4% vs. 25.9%, *p* = 0.952). Complete coverage reports are provided in the Supplementary material.

## 4 Discussion

This study found that recovery with the simplified combined program was not inferior to that with the standard program, even though the children reported greater anthropometric severity at admission. The same was observed in the ComPAS study, a randomized controlled trial conducted in non-emergency settings in Kenya and South Sudan (26), although the recovery proportion was markedly lower (44 and 48% in the control and simplified protocol groups, respectively). This difference was mainly due to the high proportion of defaulters recorded (31 and 25%, respectively), whereas in the present study, the proportion of defaulters did not exceed 4% in any group. It should be noted that the ComPAS study did not include CHWs; therefore, treatment was provided only by nurses at health centers. Our previous studies in Mali have shown that the decentralization of treatment with CHWs results in lower default rates by reducing geographical and economic access barriers for families (17–19). In addition, the recovery rates found here for the simplified group (92.9%) were very close to the 92.3% reported in a large Malian cohort of 27,800 children treated in non-emergency settings in the southwest with the same combined, simplified protocol and also involving CHWs as treatment providers (44). Moreover, in the present study, high recovery rates of over 90% were achieved in the most vulnerable group of children admitted; that is, those weighing less than 5 kg.

However, it is worth mentioning that the present study sought to compare the effectiveness of the new simplified protocol by considering the entire set of programmatic adaptations applied simultaneously (changes in the admission and discharge criteria, plus changes in the treatment product and its quantity calculation). This program design resulted in groups with different patient profiles owing to different anthropometric criteria. This differentiates it from the ComPAS study, which focused on evaluating the effect of the change in product and dose exclusively and matched the entry and discharge criteria of the control and intervention groups by considering only MUAC as an anthropometric indicator (26). To compare our results more accurately, we performed an effectiveness analysis of a subgroup of children admitted and discharged with MUAC, similar to the ComPAS study. The results showed that the recovery rate in the simplified group (97.7%) was similar to that in the control and iCCM standard groups (91.4 and 95.5%, respectively), as were the time to recovery (43 vs. 42 days) and anthropometric gain. In SAM cases, the RUTF expenditure with the simplified protocol (77 sachets) was significantly lower than that of the control (120 sachets) and iCCM standard (100 sachets) groups, and the recovery rates were also similar (96.7% vs. 93.9 and 93.0%, respectively).

Different simplified protocols have shown recovery rates comparable to those of traditional treatments. The OptiMA protocol proposes reducing the RUTF dose by increasing the weight or MUAC rather than increasing the RUTF, as is the common practice. Children with MUAC < 115 mm or edema receive 175 Kcal/kg/day of RUTF, those with MUAC between 115 and 119 mm receive 125 kcal/kg/day, and those with MUAC > 120 mm receive 75 kcal/kg/day. This new model has demonstrated recovery rates of 86.3% in a trial conducted in Burkina Faso (45) and between 72 and 96% in observational studies conducted in the Democratic Republic of Congo (46, 47). Another study conducted in Myanmar, in which the standard protocol was maintained until specific anthropometric values were reached (MUAC ≥ 110 mm and WHZ ≥ −3 z-score), and then a fixed dose of one sachet/day was applied, also recorded recovery rates above 90% (48). The Modelling an Alternative Nutrition Protocol Generalizable for Outpatient (MANGO) study in Burkina Faso applied a similar 2-stage methodology for dose calculation. Specifically, it provided the standard protocol for 2 weeks for all children (150–200 Kcal/kg/day) and reduced this from the third week onwards with a higher percentage of reduction based the higher weight of the child (−13% for those between 3 and 3.5 kg to −53% for those between 10 and 15 kg). This modified protocol achieved a recovery rate of 52.7%, which was not significantly different from the standard protocol (55.4%) (49). The recovery rates of the present study are similar to or better than those reported in other simplified treatment models but facilitate case management by simplifying the dose calculation of the nutritional product from the beginning of the treatment.

In connection with this, when analyzing the results by treatment provider in the present study, we found that CHWs with the standard protocol did not reach the SPHERE limit of 75% recovery for SAM (31) due to a high proportion of early discharge cases (above 30%). In the group with the simplified protocol, the CHWs recorded far fewer early discharge cases (13%), although this was still a greater proportion than that of nurses with the same protocol (2%). It should be noted that the proportion of early discharge of nurses in the control group (18.6%) was much higher than in the other groups (3.2% vs. 2.3%). This could be due to work overload in health centers, which may be reduced by opening new treatment sites with CHWs. Our previous studies have shown that CHWs identify more comorbidities in children with malnutrition than nurses in health centers, which could be related to the greater availability of time per patient (19, 50).

Our study’s results differ from those reported in the aforementioned Malian cohort in which CHWs with a simplified protocol obtained slightly better recovery results than nurses (44), highlighting the importance of simplifying the management of cases in complex contexts such as Gao, where there are fewer skilled human resources and CHWs possess less experience and literacy (51, 52). Another Malian study highlighted the importance of supportive supervision to ensure the quality of SAM treatment provided by CHWs when the program was scaled to larger regions (15).

As for other treatment outcomes, children with SAM treated under the simplified combined program recorded lower RUTF expenditure until recovery compared to both groups with the standard protocol, with a similar anthropometric gain for the entire sample and the SAM subgroup. In the MAM cases, the number of RUTF sachets expended in the simplified group was also lower than that in the control group (35 vs. 48). Similar results were found in the ComPAS study conducted in Kenya and South Sudan (26); however, in the present study, the average time to recovery in the simplified group was shorter (43 vs. 65 days), and the expenditure of RUTF sachets was lower (49 vs. 122). Compared with the results of a large southwestern Mali cohort treated with CHWs and the same simplified protocol (44), the SAM cases in the present study also had a shorter LOS (42 vs. 56 days) and lower RUTF sachet expenditure (77 vs. 96 days), with similar anthropometry at admission. The opposite was observed in the MAM group, with a longer LOS (48 vs. 28 days) but lower RUTF sachet expenditure than in the control cohort (35 vs. 42). This could be explained by the fact that in the present study, the proportion of MAM cases younger than 24 months was higher (93.2% vs. 77.4%). In addition, seven sachets of RUTF were administered after recovery was achieved.

Recommendations for the management of MAM in emergency settings (53) include using RUTF as a primary alternative to RUSF stock-out to avoid SAM deterioration and reduce the risk of death. These recommendations state that the nutritional composition of RUTF is very similar to that of RUSF if provided at a dose of one sachet/day, as in the present study. Studies, such as those by James et al. (54) in Ethiopia, have shown that a lack of access to specific programs for MAM cases results in high rates of deterioration owing to SAM and a low rate of recovery once patients start SAM treatment, with an average LOS of approximately 9 weeks (63 days). Other studies with different combined, simplified protocols, such as OptiMA, also reported reduced deterioration rates in the SAM arm compared with the standard arm (5% vs. 16%) (46).

A study conducted in Mali has shown that the treatment of MAM with any product is cost-effective compared to non-treatment owing to the significant reduction in the risk of death (RUSF being the most cost-effective product compared to different types of fortified flour) (55). In addition, the ComPAS study showed that treatment with the combined simplified protocol using RUTF reduces costs compared to the traditional protocol, with an overall cost reduction of 12% and $123 less per case recovered, although important differences were found between the countries included in the study (26). Conversely, iCCM programs decentralizing SAM treatment with CHWs have shown improved cost-effectiveness, with a significant reduction in societal costs applicable to families, thus facilitating access to treatment (18, 56). Merging these simplified approaches could translate into substantial savings for health systems by ensuring a continuum of care for at-risk children (57). Other programmatic changes related to screening could provide additional benefits, such as the Family-MUAC approach, in which CHWs train caregivers to detect malnutrition early in their homes so that treatment can begin with lower anthropometric severity (44, 57).

The present study shows that using the ComPAS simplified protocol and adding CHWs as treatment providers outside health facilities in emergency settings can effectively improve SAM coverage while maintaining effectiveness and reducing expenditures on nutritional intrants. A recent systematic review of post-conflict situations stated that in contexts with high disease burdens and weak infrastructure, CHWs are key actors in increasing access to basic healthcare (58). The present study showed a significant increase in SAM coverage in the groups that incorporated CHWs. Conversely, in the control group, where treatment was only provided in health centers, respectively, SAM coverage decreased. A similar positive impact of decentralizing treatment with CHWs closer to the communities on SAM coverage has already been reported in the development contexts of Mali (16), Angola (59), Bangladesh (60), Mauritania (61), Niger (50), or Tanzania (62). Regarding MAM, the coverage increased in all groups, although with less marked differences; this result may be due to the fact that during the project, the supply of RUSF to the health centers was assured, thereby avoiding the usual stock-out that occurs in emergency contexts, especially in recent years, since the World Food Program funding for MAM treatment has been reduced (63).

In the present study, the group of CHWs applying the simplified protocol significantly increased coverage by 42.5%. This result differs from that reported in the ComPAS study, where no differences in coverage were found between groups using standard or simplified protocols (26). However, the ComPAS study’s rationale stated that reducing expenditures on nutritional products would facilitate an increase in coverage and, in turn, increase the public health impact of treatment in a resource-constrained environment by extending treatment to more children. Other studies have reported an association between increased coverage and reduced expenditures on nutritional products, as in a randomized controlled trial developed by Maust et al. in the post-conflict setting of Sierra Leone (64). In that study, the authors found that co-treating SAM and MAM cases with RUTF at a reduced dose resulted in higher coverage (71% vs. 55% in the control group using the standard country protocol).

Nevertheless, the coverage figures reported in the present study were markedly lower than those reported in the aforementioned studies, with none of the groups reaching the 50% required by the SPHERE standards for acute malnutrition treatment programs in rural settings (31). A previous geospatial study conducted in another rural region of Mali (20) emphasized the importance of not only increasing the number of CHWs but also considering their location to avoid leaving high-density underserved areas and overlapping with other provision sites. This study also highlights the need to maintain malnutrition screening at the community level for the iCCM approaches to be effective for treatment coverage. In addition, studies have indicated that one of the most relevant barriers to achieving high treatment coverage is the stock-out of nutritional input (5, 12). CHWs are often located in remote and difficult-to-access locations compared to health centers, which makes the supply chain more complex. In this study, local authorities at the district level ensured supply during the study period; therefore, there were no stockouts. In addition, the Malian government usually prioritizes emergency areas over development areas, where stockouts may be a common problem. The simplified combined protocol could be a viable solution for remote areas using a single product and markedly reduce the expense of sachets recovered per child.

The present study has several limitations. First, it involved 32 treatment delivery sites, of which 12 health centers were randomized. Applying a randomization ratio of 2:1:1 to avoid sampling imbalance resulted in the inclusion of only three health centers in the intervention groups, which may have reduced the variability of the sample compared with the control group. The sample size for the SAM cases initially calculated could not be reached due to security problems in the field that prevented data collectors from accessing treatment sites during certain periods. Nevertheless, the statistical power achieved ensured non-inferiority of recovery in the simplified iCCM group. Additionally, there was a sampling imbalance between groups, although robust statistical tests were applied to this type of sampling distribution. Furthermore, limitations on access to treatment sites due to security issues limited the verification of anthropometric variables, especially WHZ, in groups with the standard protocol. Therefore, it was not possible to accurately assess the cases of early discharge, which may have been different in these groups. Likewise, no information is available on MAM cases treated by nurses; therefore, comparing their performance with that of CHWs was impossible.

Regarding strengths, this is the first study to apply a randomized controlled trial methodology in an emergency context and combine the decentralization of treatment with CHWs with several other simplified approaches proposed by UNICEF (10). The progress of the study was followed by an advisory committee formed by local, national, and West African regional authorities and organizations at various points in its development. The results are similar to those reported by other studies in which CHWs or similar simplified protocols were included, which underpins the evidence reported to date. In addition, we analyzed the possible impact of this new approach on the management of the most vulnerable cases of children under 5 kg and found positive outcomes in this group.

Future studies should focus on testing this decentralized, combined, and simplified treatment approach on a single scale, including larger geographic regions and more treatment providers. Furthermore, this approach must be tested in contexts that differ from Mali in terms of geography, climate conditions, and population composition, such as East Africa and Asian countries, where the prevalence of acute malnutrition is even higher (2). Given reports of significant reductions in nutritional product expenditures and similar anthropometric gains, the cost-effectiveness of the intervention should be analyzed to provide values of expenditure per child treated or disability-adjusted life-years averted (DALYs) to make it easier for policymakers to quantify the benefits of pooling all these modifications compared to the current protocol. Additionally, future studies should analyze the risk of relapse in each treatment model to assess the real impact of cases discharged as recovered without having reached anthropometric recovery and the overall impact on health systems.

## 5 Conclusion

The simultaneous application of several simplified approaches in emergency settings has the potential to increase treatment coverage while reducing the amount of ready-to-use food used for treatment and assuring children’s recovery. Additionally, treating MAM within the same program as SAM using a single nutritional product (RUTF) in an integrated manner with other preventive and curative activities can ensure a continuum of care.

The evidence generated by the present study could help policymakers promote the implementation of simplified combined treatment programs in complex contexts where access to treatment centers and supply chains could be limited, allowing more children to be treated and reaching them in a less severe stage, thus reducing their risk of death.

## Data availability statement

The raw data supporting the conclusions of this article will be made available by the authors, without undue reservation.

## Ethics statement

The studies involving humans were approved by Ethics Committee of the Institut National de Santé Publique (INSP), the reference agency of the Ministry of Health of the government of Mali (decision n° 35/2029/CE-EX-INRSP), and the Ethics Committee of the Hospital Clínico San Carlos, reference organism for human studies of the Complutense University of Madrid, Spain (favorable report C.I. 19/363-R-X-BC). The studies were conducted in accordance with the local legislation and institutional requirements. Written informed consent for participation in this study was provided by the participants’ legal guardians/next of kin.

## Author contributions

NL-E: Conceptualization, Data curation, Formal analysis, Methodology, Writing – original draft. PC-C: Conceptualization, Funding acquisition, Investigation, Project administration, Resources, Supervision, Validation, Writing – review & editing. SS: Project administration, Resources, Supervision, Validation, Writing – review & editing. AD: Funding acquisition, Project administration, Resources, Supervision, Validation, Writing – review & editing. LS-M: Data curation, Formal analysis, Validation, Visualization, Writing – review & editing. MS: Supervision, Validation, Writing – review & editing. AB: Supervision, Validation, Writing – review & editing. MB: Funding acquisition, Project administration, Resources, Validation, Writing – review & editing. AV: Conceptualization, Funding acquisition, Supervision, Validation, Writing – review & editing. SG: Conceptualization, Validation, Writing – review & editing. FT: Funding acquisition, Project administration, Resources, Supervision, Validation, Writing – review & editing.
