# Supplementary material for: Effectiveness of decentralizing outpatient acute malnutrition treatment with community health workers and a simplified combined protocol: a cluster randomized controlled trial in emergency settings of Mali
Source: Front Public Health. 2024 Feb 21;12:1283148. doi: 10.3389/fpubh.2024.1283148 (PMC10915236; doi:10.3389/fpubh.2024.1283148)
Supplement: Supplementary file 1 [file Data_Sheet_1.docx]

Effectiveness of decentralizing outpatient acute malnutrition treatment with Community Health Workers and a simplified-combined protocol: a cluster randomized controlled trial in emergency settings of Mali

Supplementary Material

| **Table S1.** Treatment provision sites (villages) by study group | | | |
| --- | --- | --- | --- |
|  | **Control group**  **(CMAM)** | **Intervention 1**  **(ICCM Standard)** | **Intervention 2**  **(ICCM simplified)** |
| **Health Centers** (Nurses) | Aljanabandia | Forgho | Bagnadji |
|  | Djoulabougou Saneye | Lobou | Magnadawe |
|  | Gadeye | Zinda | Wabaria |
|  | Kochakareye |  |  |
|  | Tacharane |  |  |
|  | Tin Aouker |  |  |
| **Community Health Workers** |  | Banikane | Arhabou |
|  |  | Doubaria Gourma | Baringouma |
|  |  | Gabame | Battale |
|  |  | Gorom Gorom | Kadji |
|  |  | Kardjimé | Karibandia Gourma |
|  |  | Koïma | Kokorom |
|  |  | Koissa I | Korom |
|  |  | Marga | Oura Migno |
|  |  | Tianame | Seyna Haoussa |
|  |  | Traore | Tandagari |


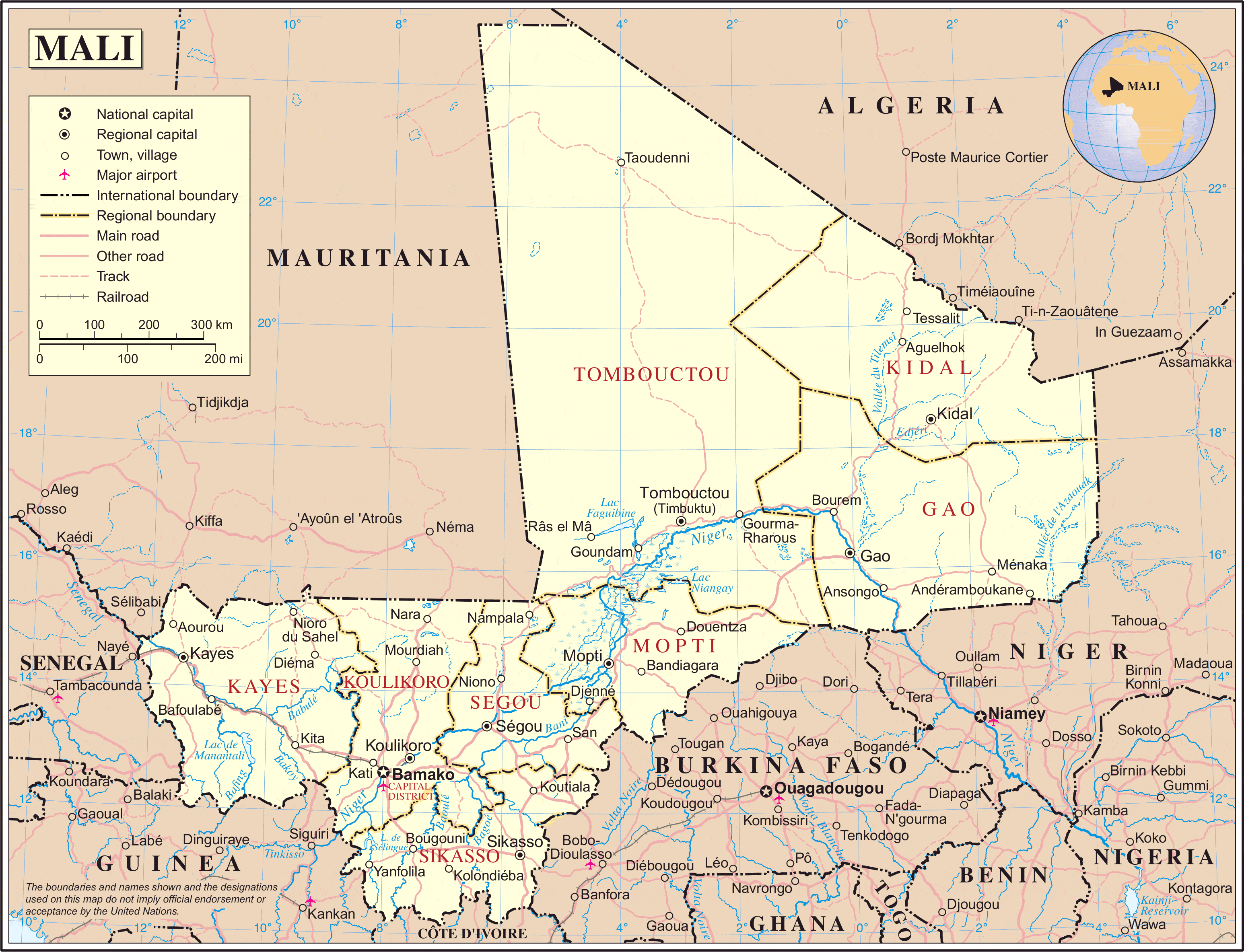

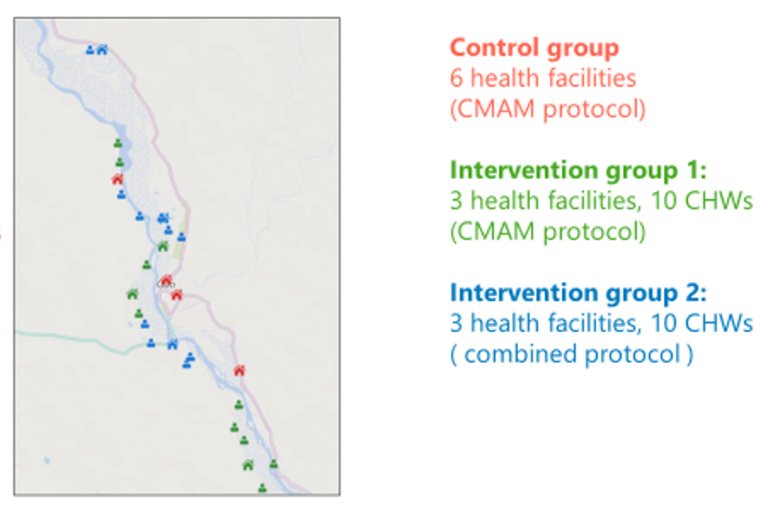


**Figure S1.** Treatment provision sites location in the Gao Region of northern Mali

**Table S2:** CONSORT 2010 checklist for reporting a cluster randomized trial

| Section/Topic | Item No | Standard Checklist item | Extension for cluster designs | Location* |
| --- | --- | --- | --- | --- |
| Title and abstract | | | |  |
|  | 1a | Identification as a randomised trial in the title | Identification as a cluster randomised trial in the title | **Title** |
|  | 1b | Structured summary of trial design, methods, results, and conclusions (for specific guidance see CONSORT for abstracts) | See table 2 | **Abstract** |
| Introduction | | | |  |
| Background and objectives | 2a | Scientific background and explanation of rationale | Rationale for using a cluster design | **Introduction paragraphs 1 to 6** |
|  | 2b | Specific objectives or hypotheses | Whether objectives pertain to the cluster level, the individual participant level or both | **Introduction paragraph 7** |
| Methods | | | |  |
| Trial design | 3a | Description of trial design (such as parallel, factorial) including allocation ratio | Definition of cluster and description of how the design features apply to the clusters | **Section 2.2** |
|  | 3b | Important changes to methods after trial commencement (such as eligibility criteria), with reasons |  | **Not applicable** |
| Participants | 4a | Eligibility criteria for participants | Eligibility criteria for clusters | **Section 2.1 and 2.2** |
|  | 4b | Settings and locations where the data were collected |  | **Supporting info Table S3 and Figure S1** |
| Interventions | 5 | The interventions for each group with sufficient details to allow replication, including how and when they were actually administered | Whether interventions pertain to the cluster level, the individual participant level or both | **Section 2.1** |
| Outcomes | 6a | Completely defined pre-specified primary and secondary outcome measures, including how and when they were assessed | Whether outcome measures pertain to the cluster level, the individual participant level or both | **Section 2.1** |
|  | 6b | Any changes to trial outcomes after the trial commenced, with reasons |  | **Section 2.5** |
| Sample size | 7a | How sample size was determined | Method of calculation, number of clusters(s) (and whether equal or unequal cluster sizes are assumed), cluster size, a coefficient of intracluster correlation (ICC or *k*), and an indication of its uncertainty | **Section 2.2** |
|  | 7b | When applicable, explanation of any interim analyses and stopping guidelines |  | **Not applicable** |
| Randomisation: | | | |  |
| Sequence generation | 8a | Method used to generate the random allocation sequence |  | **Section 2.2** |
|  | 8b | Type of randomisation; details of any restriction (such as blocking and block size) | Details of stratification or matching if used | **Section 2.2** |
| Allocation concealment mechanism | 9 | Mechanism used to implement the random allocation sequence (such as sequentially numbered containers), describing any steps taken to conceal the sequence until interventions were assigned | Specification that allocation was based on clusters rather than individuals and whether allocation concealment (if any) was at the cluster level, the individual participant level or both | **Section 2.2** |
| Implementation | 10 | Who generated the random allocation sequence, who enrolled participants, and who assigned participants to interventions | Replace by 10a, 10b and 10c |  |
|  | 10a |  | Who generated the random allocation sequence, who enrolled clusters, and who assigned clusters to interventions | **Section 2.2** |
|  | 10b |  | Mechanism by which individual participants were included in clusters for the purposes of the trial (such as complete enumeration, random sampling) | **Section 2.2** |
|  | 10c |  | From whom consent was sought (representatives of the cluster, or individual cluster members, or both), and whether consent was sought before or after randomisation | **Section 2.4** |
|  |  |  |  |  |
| Blinding | 11a | If done, who was blinded after assignment to interventions (for example, participants, care providers, those assessing outcomes) and how |  | **Not applicable** |
|  | 11b | If relevant, description of the similarity of interventions |  | **Not applicable** |
| Statistical methods | 12a | Statistical methods used to compare groups for primary and secondary outcomes | How clustering was taken into account | **Section 2.5** |
|  | 12b | Methods for additional analyses, such as subgroup analyses and adjusted analyses |  | **Section 2.5** |
| Results | | | |  |
| Participant flow (a diagram is strongly recommended) | 13a | For each group, the numbers of participants who were randomly assigned, received intended treatment, and were analysed for the primary outcome | For each group, the numbers of clusters that were randomly assigned, received intended treatment, and were analysed for the primary outcome | **Figure 1** |
|  | 13b | For each group, losses and exclusions after randomisation, together with reasons | For each group, losses and exclusions for both clusters and individual cluster members | **Figure 1** |
| Recruitment | 14a | Dates defining the periods of recruitment and follow-up |  | **Section 2.1** |
|  | 14b | Why the trial ended or was stopped |  | **Not Applicable** |
| Baseline data | 15 | A table showing baseline demographic and clinical characteristics for each group | Baseline characteristics for the individual and cluster levels as applicable for each group | **Tables 2-3 and Supporting info tables S5-S7** |
| Numbers analysed | 16 | For each group, number of participants (denominator) included in each analysis and whether the analysis was by original assigned groups | For each group, number of clusters included in each analysis | **Not applicable** |
| Outcomes and estimation | 17a | For each primary and secondary outcome, results for each group, and the estimated effect size and its precision (such as 95% confidence interval) | Results at the individual or cluster level as applicable and a coefficient of intracluster correlation (ICC or k) for each primary outcome | **Figure 2 and tables 4. Supporting info figure S2 and tables S9-S11** |
|  | 17b | For binary outcomes, presentation of both absolute and relative effect sizes is recommended |  | **Tables 4 and S9 and figures 2 and S2** |
| Ancillary analyses | 18 | Results of any other analyses performed, including subgroup analyses and adjusted analyses, distinguishing pre-specified from exploratory |  | **Table 6 and supporting info table S10-S14** |
| Harms | 19 | All important harms or unintended effects in each group (for specific guidance see CONSORT for harms) |  | **Not applicable** |
| Discussion | | | |  |
| Limitations | 20 | Trial limitations, addressing sources of potential bias, imprecision, and, if relevant, multiplicity of analyses |  | **Third paragraph starting from the end of the discussion** |
| Generalisability | 21 | Generalisability (external validity, applicability) of the trial findings | Generalisability to clusters and/or individual participants (as relevant) | **Third paragraph starting from the end of the discussion** |
| Interpretation | 22 | Interpretation consistent with results, balancing benefits and harms, and considering other relevant evidence |  | **All discussion** |
| Other information | | |  |  |
| Registration | 23 | Registration number and name of trial registry |  | **Section 2.4** |
| Protocol | 24 | Where the full trial protocol can be accessed, if available |  | **Section 2.4** |
| Funding | 25 | Sources of funding and other support (such as supply of drugs), role of funders |  | **Section 2.4 and**  **Funding Statement** |

**Table S3:** CONSORT Statement 2006 checklist for non-inferiority and equivalence trials

| ***PAPER SECTION* And topic** | 1. **Item** | **Descriptor** | **Reported on**  **Location** |
| --- | --- | --- | --- |
| 1. *TITLE & ABSTRACT* | 1 | [How participants were allocated to interventions](http://www.consort-statement.org/index.aspx?o=1107) (*e.g*., "random allocation", "randomized", or "randomly assigned"),  s*pecifying that the trial is a non-inferiority or equivalence trial.* | Title and abstract |
| *INTRODUCTION* Background | 2 | [Scientific background and explanation of rationale](http://www.consort-statement.org/index.aspx?o=1016),  *including the rationale for using a non-inferiority or equivalence design.* | Introduction paragraph 4 |
| *METHODS* Participants | 3 | [Eligibility criteria for participants](http://www.consort-statement.org/index.aspx?o=1017#3a) *(detailing whether participants in the non-inferiority or equivalence trial are similar to those in any trial(s) that established efficacy of the reference treatment)* and the [settings and locations where the data were collected](http://www.consort-statement.org/index.aspx?o=1017#3b). | Section 2.1, 2.2 and supporting info table S1-figure S1 |
| Interventions | 4 | [Precise details of the interventions intended for each group *detailing whether the reference treatment in the non-inferiority or equivalence trial is identical (or very similar) to that in any trial(s) that established efficacy,* and how and when they were actually administered](http://www.consort-statement.org/index.aspx?o=1021). | Section 2.1 |
| Objectives | 5 | [Specific objectives and hypotheses](http://www.consort-statement.org/index.aspx?o=1022), *including the hypothesis concerning non-inferiority or equivalence*. | Section 2.1 and 2.2 |
| Outcomes | 6 | [Clearly defined primary and secondary outcome measures](http://www.consort-statement.org/index.aspx?o=1023#6a) *detailing whether the outcomes in the non-inferiority or equivalence trial are identical (or very similar) to those in any trial(s) that established efficacy of the reference treatment* and, when applicable, any [methods used to enhance the quality of measurements](http://www.consort-statement.org/index.aspx?o=1023#6b) (*e.g.*, multiple observations, training of assessors). | Section 2.1 and 2.4 |
| Sample size | 7 | [How sample size was determined](http://www.consort-statement.org/index.aspx?o=1024#7a) *detailing whether it was calculated using a non-inferiority or equivalence criterion and specifying the margin of equivalence with the rationale for its choice*. When applicable, [explanation of any interim analyses and stopping rules](http://www.consort-statement.org/index.aspx?o=1024#7b) (*and whether related to a non-inferiority or equivalence hypothesis*). | Section 2.2 |
| Randomization -- Sequence generation | 8 | [Method used to generate the random allocation sequence, including details of any restrictions](http://www.consort-statement.org/index.aspx?o=1025) (*e.g*., blocking, stratification) | Section 2.2 |
| Randomization -- Allocation concealment | 9 | [Method used to implement the random allocation sequence](http://www.consort-statement.org/index.aspx?o=1026) (*e.g*., numbered containers or central telephone), clarifying whether the sequence was concealed until interventions were assigned. | Section 2.2 |
| Randomization -- Implementation | 10 | [Who generated the allocation sequence, who enrolled participants, and who assigned participants to their groups](http://www.consort-statement.org/index.aspx?o=1027). | Section 2.2 |
| Blinding (masking) | 11 | [Whether or not participants, those administering the interventions, and those assessing the outcomes were blinded to group assignment](http://www.consort-statement.org/index.aspx?o=1028#11a). If done, [how the success of blinding was evaluated](http://www.consort-statement.org/index.aspx?o=1028#11b). | Section 2.2 |
| Statistical methods | 12 | [Statistical methods used to compare groups for primary outcome(s)](http://www.consort-statement.org/index.aspx?o=1029#12a), *specifying whether a one or two-sided confidence interval approach was used*. [Methods for additional analyses](http://www.consort-statement.org/index.aspx?o=1029#12b), such as subgroup analyses and adjusted analyses. | Section 2.5 |
| 1. *RESULTS*   Participant flow | 13 | [Flow of participants through each stage](http://www.consort-statement.org/index.aspx?o=1018) (a diagram is strongly recommended). Specifically, for each group report the numbers of participants randomly assigned, receiving intended treatment, completing the study protocol, and analyzed for the primary outcome. [Describe protocol deviations from study as planned, together with reasons](http://www.consort-statement.org/index.aspx?o=1086). | Figure 1 |
| Recruitment | 14 | [Dates defining the periods of recruitment and follow-up](http://www.consort-statement.org/index.aspx?o=1087). | Section 2.1 |
| Baseline data | 15 | [Baseline demographic and clinical characteristics of each group](http://www.consort-statement.org/index.aspx?o=1088). | Tables 2-3 and supporting info tables S5-S7 |
| Numbers analyzed | 16 | [Number of participants (denominator) in each group included in each analysis and whether the analysis was](http://www.consort-statement.org/index.aspx?o=1089) *“intention-to-treat”* *and/or* *alternative analyses were conducted*. State the results in absolute numbers when feasible (*e.g*., 10/20, not 50%). | Across all tables |
| Outcomes and estimation | 17 | [For each primary and secondary outcome, a summary of results for each group, and the estimated effect size and its precision](http://www.consort-statement.org/index.aspx?o=1090) (*e.g.*, 95% confidence interval). *For the outcome(s) for which non-inferiority or equivalence is hypothesized, a figure showing confidence intervals and margins of equivalence may be useful*. | Tables 4-6 and supporting info tables S9-S13 |
| Ancillary analyses | 18 | [Address multiplicity by reporting any other analyses performed](http://www.consort-statement.org/index.aspx?o=1091), including subgroup analyses and adjusted analyses, indicating those pre-specified and those exploratory. | Table 6 and supporting info tables S9-S13 |
| Adverse events | 19 | [All important adverse events or side effects in each intervention group](http://www.consort-statement.org/index.aspx?o=1092). | Not applicable |
| *DISCUSSION* Interpretation | 20 | [Interpretation of the results](http://www.consort-statement.org/index.aspx?o=1019), taking into account the *non-inferiority or equivalence hypothesis and any other* study hypotheses, sources of potential bias or imprecision and the dangers associated with multiplicity of analyses and outcomes. | Across all the discussion section |
| Generalizability | 21 | [Generalizability (external validity) of the trial findings](http://www.consort-statement.org/index.aspx?o=1094). | Discussion – last paragraph |
| Overall evidence | 22 | [General interpretation of the results in the context of current evidence](http://www.consort-statement.org/index.aspx?o=1095). | Across all the discussion section |

| **Table S4.** Statistical power of the sample sizes achieved in each study group. | | | |
| --- | --- | --- | --- |
| **Sample** | **Protocol comparison** | **Power (1-β)** |  |
| TOTAL | Control vs. ICCM standard | 0.9985 |  |
|  | Control vs. ICCM Simplified | 1.000 |  |
|  | iCCM Standard vs. iCCM simplified | 1.000 |  |
| SAM | Control vs. ICCM standard | 0.4927 |  |
|  | Control vs. ICCM Simplified | 1.000 |  |
|  | iCCM Standard vs. iCCM simplified | 1.000 |  |
| MAM | Control vs. ICCM standard | 0.9999 |  |
|  | Control vs. ICCM Simplified | 0.9999 |  |
|  | iCCM Standard vs. iCCM simplified | 0.9999 |  |
| iCCM: integrated community case management; MAM: moderate acute malnutrition; SAM: severe acute malnutrition. | | | |

| **Table S5.** Socioeconomic characteristics of severe acutely malnourished children at admission compared by study group. | | | | | | | | | | | | | | | | | | |
| --- | --- | --- | --- | --- | --- | --- | --- | --- | --- | --- | --- | --- | --- | --- | --- | --- | --- | --- |
| **SEVERE ACUTE MALNUTRITION CASES** | | **Control CMAM ^a^**  (n=198) | | | **iCCM standard ^b^**  (n=202) | | | | **iCCM simplified ^c^**  (n=108) | | | | a vs. b  p-value | | a vs. c p-value | | b vs. c p-value | |
|  |  | N | Mean (SD)  or % (n) | | N | | Mean (SD)  or % (n) | | N | | Mean (SD)  or % (n) | |  |  |  |  |  |  |
| **Demograph** | Cohabiting people | 198 | 7.23 (2.52) | | 202 | | 8.48 (3.74) | | 108 | | 7.68 (3.00) | | **<0.001** | | 0.231 | | 0.070 | |
|  | Children under 5 cohabiting | 192 | 1.40 (1.27) | | 194 | | 1.52 (1.83) | | 105 | | 1.17 (1.20) | | 0.440 | | 0.420 | | 0.170 | |
|  | Years of education of primary caregiver | 160 | 0.19 (0.48) | | 187 | | 0.55 (1.94) | | 105 | | 0.31 (0.58) | | **0.039** | | 0.465 | | 0.298 | |
| **Livelihoods** | Type of housing | 197 |  | | 194 | |  | | 107 | |  | |  | |  | |  | |
|  | In property |  | 88.33 (174) | |  | | 99.48 (193) | |  | | 97.20 (104) | | **<0.001** | | **0.030** | | 0.260 | |
|  | For rent |  | 10.15 (20) | |  | | 0.52 (1) | |  | | 0.93 (1) | | **<0.001** | | **0.010** | | 0.999 | |
|  | On loan |  | 1.52 (3) | |  | | 0.00 (0) | |  | | 1.87 (2) | | 0.730 | | 0.999 | | 0.730 | |
|  | With access to safe water | 198 | 12.12 (24) | | 202 | | 10.89 (22) | | 108 | | 13.89 (15) | | 0.999 | | 0.999 | | 0.999 | |
|  | With safe sanitation | 198 | 19.19 (38) | | 202 | | 1.48 (3) | | 108 | | 0.93 (1) | | **<0.001** | | **<0.001** | | 0.999 | |
|  | With electricity | 198 | 84.85 (168) | | 202 | | 26.24 (53) | | 108 | | 17.59 (19) | | **<0.001** | | **<0.001** | | 0.110 | |
|  | With arable land | 197 | 13.71 (27) | | 202 | | 91.09 (184) | | 107 | | 90.65 (97) | | **<0.001** | | **<0.001** | | 0.999 | |
|  | With livestock | 193 | 40.93 (79) | | 202 | | 64.36 (130) | | 106 | | 46.23 (49) | | **<0.001** | | 0.445 | | **0.006** | |
|  | With construction floor | 79 | 14.57 (26.62) | | 130 | | 8.65 (18.50) | | 49 | | 1.80 (2.14) | | 0.073 | | **0.001** | | 0.073 | |
|  | With construction roof | 79 | 12.31 (16.50) | | 130 | | 6.37 (8.24) | | 49 | | 3.20 (3.14) | | **<0.001** | | **<0.001** | | 0.085 | |
| **Food security** | Number of meals/day | 197 | 2.86 (0.34) | | 201 | | 2.84 (0.44) | | 108 | | 2.57 (0.57) | | 0.610 | | **<0.001** | | **<0.001** | |
|  | Lack of food last 4 weeks | 171 |  | | 201 | |  | | 108 | |  | |  | |  | |  | |
|  | Never |  | 12.86 (22) | |  | | 11.94 (24) | |  | | 22.22 (24) | | 0.910 | | 0.120 | | 0.080 | |
|  | Rarely |  | 74.27 (127) | |  | | 56.72 (114) | |  | | 54.63 (59) | | **0.002** | | **0.002** | | 0.816 | |
|  | 3-10 times |  | 11.70 (20) | |  | | 27.36 (55) | |  | | 22.22 (24) | | **<0.001** | | 0.058 | | 0.394 | |
|  | More than 10 times |  | 1.17 (2) | |  | | 3.98 (8) | |  | | 0.93 (1) | | 0.530 | | 0.999 | | 0.530 | |
|  | Food Consumption Score | 198 | 61.04 (16.59) | | 202 | | 50.24 (20.51) | | 108 | | 44.74 (18.11) | | **<0.001** | | **<0.001** | | **0.013** | |
|  | Poor diet |  | 0.50 (1) | |  | | 13.37 (27) | |  | | 5.55 (6) | | **<0.001** | | **0.031** | | **0.053** | |
|  | Limited diet |  | 7.07 (14) | |  | | 9.90 (20) | |  | | 25.93 (28) | | 0.403 | | **<0.001** | | **<0.001** | |
|  | Acceptable diet |  | 92.43 (183) | |  | | 76.73 (155) | |  | | 68.52 (74) | | **<0.001** | | **<0.001** | | 0.150 | |
| **Heath care access** | Behavior if child is sick | 198 |  | | 202 | |  | | 106 | |  | |  | |  | |  | |
|  | Health post or CHW |  | 78.28 (155) | |  | | 85.64 (173) | |  | | 84.91 (90) | | 0.220 | | 0.430 | | 0.999 | |
|  | Traditional medicine |  | 17.68 (35) | |  | | 14.36 (29) | |  | | 7.55 (8) | | 0.442 | | **0.038** | | 0.136 | |
|  | Self medication |  | 4.04 (8) | |  | | 0.00 (0) | |  | | 8.49 (9) | | **0.022** | | 0.177 | | **<0.001** | |
|  | With difficulty of access | 197 | 11.28 (22) | | 201 | | 30.35 (61) | | 107 | | 17.76 (19) | | **<0.001** | | 0.163 | | **0.047** | |
|  | Time to get to treatment | 169 |  | | 200 | |  | | 107 | |  | |  | |  | |  | |
|  | 30 minutes or less |  | 76.93 (130) | |  | | 71.50 (143) | |  | | 52.78 (57) | | 0.287 | | **<0.001** | | **0.003** | |
|  | Up to 1.5 hours |  | 11.24 (19) | |  | | 17.50 (35) | |  | | 37.04 (40) | | 0.122 | | **<0.001** | | **<0.001** | |
|  | More than 2 hours |  | 11.83 (20) | |  | | 11.00 (22) | |  | | 10.18 (11) | | 0.999 | | 0.999 | | 0.999 | |
| CHW: community health worker; CMAM: community management of acute malnutrition; iCCM: integrated community case management; SD: standard deviation. In bold the significant results. | | | | | | | | | | | | | | | | | | |
| **Table S6.** Socioeconomic characteristics of moderate acutely malnourished children at admission compared by study group. | | | | | | | | | | | | | | | | | |  |
| **MODERATE ACUTE MALNUTRITION CASES** | | **Control CMAM ^a^**  (n=4) | | **iCCM standard ^b^**  (n=124) | | | | **iCCM simplified ^c^**  (n=40) | | | | a vs. b  p-value | | a vs. c p-value | | b vs. c p-value | |  |
|  |  | N | Mean (SD)  or % (n) | N | | Mean (SD)  or % (n) | | N | | Mean (SD)  or % (n) | |  |  |  |  |  |  |  |
| **Demograph** | Cohabiting people | 4 | 7.50 (2.38) | 124 | | 9.50 (4.27) | | 40 | | 6.95 (2.58) | | 0.630 | | 0.788 | | **0.001** | |  |
|  | Children under 5 cohabiting | 4 | 1.00 (1.41) | 124 | | 1.57 (1.54) | | 35 | | 0.91 (1.82) | | 0.964 | | 0.964 | | 0.099 | |  |
|  | Years of education of primary caregiver | 3 | 0.00 (0.00) | 114 | | 0.44 (1.29) | | 34 | | 0.21 (0.48) | | 0.999 | | 0.999 | | 0.910 | |  |
| **Livelihoods** | Type of housing | 4 |  | 124 | |  | | 39 | |  | |  | |  | |  | |  |
|  | In property |  | 75.00 (3) |  | | 92.74 (115) | |  | | 79.49 (31) | | 0.999 | | 0.999 | | 0.120 | |  |
|  | For rent |  | 25.00 (1) |  | | 2.42 (3) | |  | | 12.82 (5) | | 0.547 | | 0.999 | | 0.084 | |  |
|  | On loan |  | 0.00 (0) |  | | 4.84 (6) | |  | | 7.69 (3) | | 0.999 | | 0.999 | | 0.999 | |  |
|  | With access to safe water | 4 | 25.00 (1) | 124 | | 8.87 (11) | | 40 | | 2.50 (1) | | 0.960 | | 0.960 | | 0.960 | |  |
|  | With safe sanitation | 4 | 0.00 (0) | 91 | | 0.00 (0) | | 34 | | 11.76 (4) | | - | | 0.999 | | **0.012** | |  |
|  | With electricity | 4 | 100.00 (4) | 124 | | 17.74 (22) | | 40 | | 10.00 (4) | | **0.001** | | **<0.001** | | 0.359 | |  |
|  | With arable land | 4 | 0.00 (0) | 124 | | 85.48 (106) | | 40 | | 75.00 (30) | | **<0.001** | | **0.024** | | 0.196 | |  |
|  | With livestock | 4 | 50.00 (2) | 124 | | 65.32 (84) | | 40 | | 10.00 (4) | | 0.920 | | 0.290 | | **<0.001** | |  |
|  | With construction floor | 2 | 25.00 (35.35) | 81 | | 9.70 (19.79) | | 4 | | 4.25 (2.87) | | 0.680 | | 0.680 | | 0.680 | |  |
|  | With construction roof | 2 | 9.00 (4.24) | 81 | | 5.48 (7.76) | | 4 | | 7.25 (3.20) | | 0.999 | | 0.999 | | 0.999 | |  |
| **Food security** | Number of meals/day | 4 | 2.50 (0.58) | 124 | | 2.80 (0.40) | | 40 | | 2.25 (0.54) | | 0.370 | | 0.370 | | **<0.001** | |  |
|  | Lack of food last 4 weeks | 4 |  | 124 | |  | | 40 | |  | |  | |  | |  | |  |
|  | Never |  | 0.00 (0) |  | | 6.46 (8) | |  | | 10.00 (4) | | 0.999 | | 0.999 | | 0.999 | |  |
|  | Rarely |  | 100.00 (4) |  | | 74.18 (92) | |  | | 67.50 (27) | | 0.999 | | 0.999 | | 0.999 | |  |
|  | 3-10 times |  | 0.00 (0) |  | | 18.55 (23) | |  | | 20.00 (8) | | 0.999 | | 0.999 | | 0.999 | |  |
|  | More than 10 times |  | 0.00 (0) |  | | 0.81 (1) | |  | | 2.50 (1) | | 0.999 | | 0.999 | | 0.999 | |  |
|  | Food Consumption Score | 4 | 60.25 (18.56) | 124 | | 55.17 (22.28) | | 40 | | 40.83 (19.71) | | 0.644 | | 0.177 | | **0.001** | |  |
|  | Poor diet |  | 0.00 (0) |  | | 13.71 (17) | |  | | 10.00 (4) | | 0.999 | | 0.999 | | 0.999 | |  |
|  | Limited diet |  | 0.00 (0) |  | | 7.26 (9) | |  | | 35.00 (14) | | 0.999 | | 0.786 | | **<0.001** | |  |
|  | Acceptable diet |  | 100.00 (4) |  | | 79.03 (98) | |  | | 55.00 (22) | | 0.693 | | 0.451 | | **0.016** | |  |
| **Heath care access** | Behavior if child is sick | 4 |  | 124 | |  | | 39 | |  | |  | |  | |  | |  |
|  | Health post or CHW |  | 100.00 (4) |  | | 92.74 (115) | |  | | 92.31 (36) | | 0.999 | | 0.999 | | 0.999 | |  |
|  | Traditional medicine |  | 0.00 (0) |  | | 7.26 (9) | |  | | 7.69 (3) | | 0.999 | | 0.999 | | 0.999 | |  |
|  | Self medication |  | 0.00 (0) |  | | 0.00 (0) | |  | | 0.00 (0) | | - | | - | | - | |  |
|  | With difficulty of access | 4 | 25.00 (1) | 124 | | 17.74 (22) | | 40 | | 0.00 (0) | | 0.999 | | 0.300 | | **0.028** | |  |
|  | Time to get to treatment | 3 |  | 123 | |  | | 38 | |  | |  | |  | |  | |  |
|  | 30 minutes or less |  | 100.00 (3) |  | | 81.30 (100) | |  | | 34.21 (13) | | 0.940 | | 0.200 | | **<0.001** | |  |
|  | Up to 1.5 hours |  | 0.00 (0) |  | | 10.57 (13) | |  | | 28.95 (11) | | 0.999 | | 0.999 | | **0.035** | |  |
|  | More than 2 hours |  | 0.00 (0) |  | | 8.13 (10) | |  | | 36.84 (14) | | 0.999 | | 0.999 | | **<0.001** | |  |
| CHW: community health worker; CMAM: community management of acute malnutrition; iCCM: integrated community case management; SD: standard deviation. In bold the significant results. | | | | | | | | | | | | | | | | | |  |

| **Table S7.** Admission characteristics compared between study groups for severe and moderate cases. | | | | | | | | | | | | | |
| --- | --- | --- | --- | --- | --- | --- | --- | --- | --- | --- | --- | --- | --- |
|  | | **Control CMAM ^a^** | | **iCCM standard ^b^** | | **iCCM simplified ^c^** | | a vs. b  p-value | | a vs. c  p-value | | b vs. c  p-value | |
| **SAM** | | N=371 | | N=471 | | N=364 | |  | |  | |  | |
| Sex Male, %(n) | | 45.8 (170) | | 46.7 (220) | | 48.9 (178) | | 0.835 | | 0.417 | | 0.531 | |
| Age (months) | |  | |  | |  | |  | |  | |  | |
| Median [IQR] | | 13.0 [10.0 – 19.0] | | 12.0 [9.0 – 18.0] | | 12.0 [8.0 – 16.0] | | **0.016** | | **<0.001** | | **0.013** | |
| 6 – 12, % (n) | | 47.4 (176) | | 56.3 (265) | | 62.1 (226) | |  | |  | |  | |
| 12-24, % (n) | | 43.3 (167) | | 37.5 (185) | | 34.1 (124) | | **0.032** | | **<0.001** | | 0.086 | |
| >24, % (n) | | 9.6 (37) | | 6.5 (32) | | 3.8 (14) | |  | |  | |  | |
| Anthropometry | | Median [IQR] | | Median [IQR] | | Median [IQR] | |  | |  | |  | |
| Weight (kg) | | 6.6 [5.8 -7.4] | | 6.4 [5.6 – 7.2] | | 6.0 [5.2 – 6.9] | | 0.070 | | **<0.001** | | **<0.001** | |
| WHZ (z-score) | | -3.32 [-3.78, -2.73] | | -3.29[-3.73, -2.71] | | -4.29 [-5.56, -3.16] | | 0.639 | | **<0.001** | | **<0.001** | |
| MUAC (mm) | | 112.0 [110.0 – 115.0] | | 112.0 [110.0 – 114.0] | | 111.0 [109.0 – 112.0] | | **0.029** | | **<0.001** | | **<0.001** | |
| Other diseases | | % (n) | | % (n) | | % (n) | |  | |  | |  | |
| Malaria | | 4.9 (18) | | 5.7 (27) | | 2.7 (10) | | 0.645 | | 0.177 | | 0.042 | |
| Diarrhea | | 2.4 (9) | | 1.1 (5) | | 1.5 (5) | | 0.176 | | 0.431 | | 0.749 | |
| ARI | | 12.3 (45) | | 13.1 (59) | | 4.6 (15) | | 0.752 | | **<0.001** | | **<0.001** | |
| **MAM** | | N=178 | | N=329 | | N=325 | |  | |  | |  | |
| Sex Male, %(n) | | 38.2 (68) | | 48.9 (161) | | 49.5 (161) | | **0.025** | | **0.015** | | 0.938 | |
| Age (months) | |  | |  | |  | |  | |  | |  | |
| Median [IQR] | | 17.0 [10.0 – 23.0] | | 14.0 [11.8 – 21.0] | | 13.0 [9.0 – 19.0] | | 0.217 | | **0.002** | | **0.021** | |
| 6 – 12, % (n) | | 35.4 (63) | | 45.6 (150) | | 47.4 (154) | | **0.033** | | **0.001** | | 0.388 | |
| 12-24, % (n) | | 48.9 (87) | | 44.7 (147) | | 45.8 (149) | |  |  |  |  |  |  |
| >24, % (n) | | 15.7 (28) | | 9.7 (32) | | 6.8 (22) | |  |  |  |  |  |  |
| Anthropometry | | Median [IQR] | | Median [IQR] | | Median [IQR] | |  | |  | |  | |
| Weight (kg) | | 7.9 [6.8 – 9.0] | | 7.5 [6.9 – 8.4] | | 6.5 [5.6 – 7.1] | | 0.055 | | **<0.001** | | **<0.001** | |
| WHZ (z-score) | | -2.36 [-2.91, -2.09] | | -2.19 [-2.65, -1.75] | | -2.41 [-2.95, -2.19] | | **0.002** | | 0.211 | | **0.001** | |
| MUAC (mm) | | 120.0 [118.0 – 123.0] | | 120.0 [119.0 -122.0] | | 120.0 [118.0 – 122.0] | | 0.426 | | 0.495 | | 0.859 | |
| Other diseases | | % (n) | | % (n) | | % (n) | |  | |  | |  | |
| Malaria | | 0 (0) | | 0.6 (2) | | 0.3 (1) | | 0.543 | | 1.000 | | 1.000 | |
| Diarrhea | | 0 (0) | | 0.7 (2) | | 3.1 (8) | | 1.000 | | 0.055 | | **0.049** | |
| ARI | | 27.2 (34) | | 23.8 (55) | | 2.3 (5) | | 0.522 | | **<0.001** | | **<0.001** | |
| ARI: acute respiratory infection; CMAM: community management of acute malnutrition; iCCM: integrated community case management; MUAC: mid-upper arm circumference; SAM: severe acute malnutrition; MAM: moderate acute malnutrition; MUAC: mid-upper arm circumference; WHZ: weight-for-height z-score. In bold the significant results. | | | | | | | | | | | | | |

| **Table S8.** Difference in the recovery proportion in the pairwise comparisons of study groups and intra-cluster correlation coefficient for recovery in the total sample and by severity. | |
| --- | --- |
| **TOTAL SAMPLE** | **Intra-cluster correlation coefficient [95% C.I.]** |
| iCCM standard vs. Control CMAM | 0.0007 [0.0006 to 0.0008] |
| iCCM simplified vs. Control CMAM | 0.06528 [0.0597 to 0.0708] |
| iCCM simplified vs. iCCM standard | 0.0457 [0.0418 to 0.0496] |
| **SAM** |  |
| iCCM Standard vs. Control CMAM | -0.0023 [-0.0023 to -0.0022] |
| iCCM simplified vs. Control CMAM | 0.0486 [0.0445 to 0.0528] |
| iCCM simplified vs. iCCM standard | 0.04831 [0.0442 to 0.0524] |
| **MAM** |  |
| iCCM standard vs. Control CMAM | 0.0023 [0.0021 to 0.0025] |
| iCCM simplified vs. Control CMAM | 0.0811 [0.0741 to 0.0880] |
| iCCM simplified vs. iCCM S standard | 0.0406 [0.0372 to 0.0441] |
| C.I. Confidence Interval; CMAM: community management of acute malnutrition; iCCM: integrated community case management; MAM: moderate acute malnutrition; SAM: severe acute malnutrition. | |

| **Table S9.** Sphere Standards treatment outcomes compared by study group for the total sample and severe and moderate cases (per-protocol analysis). | | | | | | | | | |
| --- | --- | --- | --- | --- | --- | --- | --- | --- | --- |
|  | **Control CMAM ^a^**  % (n) | **iCCM standard ^b^**  % (n) | **iCCM simplified ^c^**  % (n) | b vs. a  Risk difference [95% C.I.]* | p-value | c vs. a  Risk difference [95% C.I.]* | p-value | c vs. b  Risk difference [95% C.I.]* | p-value |
| **TOTAL SAMPLE** | N=439 | N=678 | N=643 |  |  |  |  |  |  |
| Recovered | 95.4 (419) | 96.5 (654) | 99.5 (640) | 0.01 [-0.02 – 0.03] | 0.224 | **0.04 [0.02 – 0.06]** | **<0.001** | **0.03 [0.01 – 0.05]** | **<0.001** |
| Defaulted | 4.6 (20) | 3.4 (23) | 0.5 (3) | -0.01 [-0.03 – 0.02] | 0.255 | -- |  | -- |  |
| Death | 0 (0) | 0.1 (1) | 0 (0) | -- |  | -- |  | -- |  |
| **SAM** | N=269 | N=357 | N=320 |  |  |  |  |  |  |
| Recovered | 98.5 (265) | 94.4 (337) | 99.7 (319) | **-0.03 [-0.06 – -0.01]** | **0.010** | 0.01 [-0.01 - 0.03] | 0.066 | **0.04 [0.02 – 0.06]** | **<0.001** |
| Defaulted | 1.5 (4) | 5.3 (19) | 0.3 (1) | -- |  | -- |  | -- |  |
| Death | 0 (0) | 0.3 (1) | 0 (0) | -- |  | -- |  | -- |  |
| **MAM** | N=170 | N=321 | N=323 |  |  |  |  |  |  |
| Recovered | 90.6 (154) | 98.8 (317) | 99.4 (321) | **0.05 [0.01 – 0.09]** | **0.003** | **0.07 [0.02 – 0.11]** | **0.003** | 0.01 [-0.02 – 0.04] | 0.165 |
| Defaulted | 9.4 (16) | 1.2 (4) | 0.6 (2) | -- |  | -- |  | -- |  |
| Death | -- | -- | -- | -- |  | -- |  | -- |  |
| *Adjusted for cluster, sex, age, and anthropometry at admission only performed for recovered, defaulted, and early discharge because the remainder of the outcomes registered less than 5% of cases in the 3 study groups. The significant results are in bold. CI: confidence interval; CMAM: community management of acute malnutrition model; iCCM: integrated community case management; SAM: severe acute malnutrition; MAM: moderate acute malnutrition. | | | | | | | | | |

**Figure S2.** Risk difference in the recovery proportion of study groups adjusted by clusters, for the total sample and by severe (SAM) and moderate (MAM) cases considering only the Sphere Standards outcomes (per-protocol analysis)


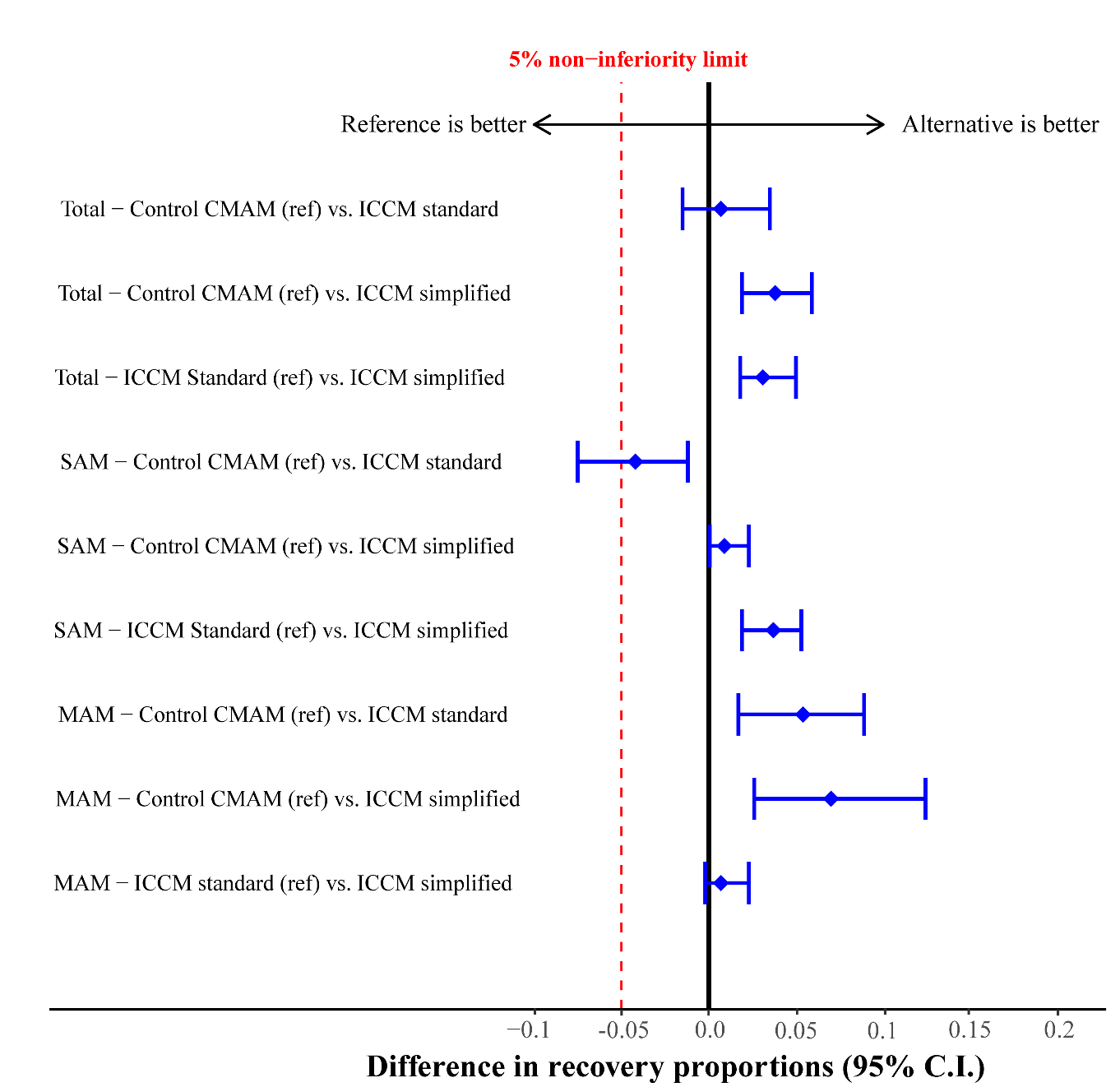


| **Table S10.** Treatment outcomes of the MUAC-only subsamples (those admitted with MUAC < 125mm in all groups). | | | | | | | | | |
| --- | --- | --- | --- | --- | --- | --- | --- | --- | --- |
|  | **Control CMAM ^a^**  % (n) | **iCCM standard ^b^**  % (n) | **iCCM simplified ^c^**  % (n) | b vs. a  Risk difference [95% C.I.]* | p-value | c vs. a  Risk difference [95% C.I.]* | p-value | c vs. b  Risk difference [95% C.I.]* | p-value |
| **TOTAL SAMPLE** | N=327 | N=664 | N=655 |  |  |  |  |  |  |
| Recovered | 91.4 (299) | 95.5 (634) | 97.7 (640) | **0.07 [0.03 – 0.07]** | **<0.001** | **0.10 [0.06 – 0.14]** | **<0.001** | **0.03 [0.01 – 0.05]** | **<0.001** |
| Defaulted | 6.1 (20) | 3.5 (23) | 0.5 (3) | -0.03 [-0.06 – 0.01] | 0.051 | **--** | **--** | **--** | -- |
| Non-response | 0 (0) | 0.2 (1) | 0.9 (6) | -- | -- | -- | -- | -- | -- |
| Referenced | 2.4 (8) | 0.8 (5) | 0.6 (4) | **--** | **--** | **--** | -- | -- | -- |
| Transferred | 0 (0) | 0 (0) | 0.3 (2) | -- | -- | -- | -- | -- | -- |
| Death | 0 (0) | 0.2 (1) | 0 (0) | -- | -- | -- | -- | -- | -- |
| **SAM **** | N=197 | N=341 | N=330 |  |  |  |  |  |  |
| Recovered | 93.9 (185) | 93.0 (317) | 96.7 (319) | -0.02 [-0.08 – 0.04] | 0.289 | **0.07 [0.02 – 0.12]** | **0.003** | **0.06 [0.03 – 0.09]** | **<0.001** |
| Defaulted | 2.0 (4) | 5.6 (19) | 0.3 (1) | -- | -- | -- | **--** | **--** | -- |
| Non-response | 0 (0) | 0 (0) | 1.5 (5) | -- | -- | -- | **--** | **--** | -- |
| Referenced | 4.1 (8) | 1.2 (4) | 0.9 (3) | **--** | **--** | **--** | -- | -- | -- |
| Transferred | 0 (0) | 0 (0) | 0.6 (2) | -- | -- | -- | -- | -- | -- |
| Death | 0 (0) | 0.3 (1) | 0 (0) | -- | -- | -- | -- | -- | -- |
| **MAM ***** | N=130 | N=323 | N=325 |  |  |  |  |  |  |
| Recovered | 87.7 (114) | 98.1 (317) | 98.8 (321) | **0.06 [0.01 – 0.11]** | **0.012** | **0.08 [0.03 – 0.14]** | **0.002** | **0.01 [-0.03 – 0.04]** | **0.010** |
| Defaulted | 12.3 (16) | 1.2 (4) | 0.6 (2) | **--** | **--** | **--** | -- | -- | -- |
| Non-response | 0 (0) | 0.3 (1) | 0.3 (1) | -- | -- | -- | -- | -- | -- |
| Referenced | 0 (0) | 0.3 (1) | 0.3 (1) | -- | -- | -- | -- | -- | -- |
| Transferred | 0 (0) | 0 (0) | 0 (0) | -- | -- | -- | -- | -- | -- |
| Death | 0 (0) | 0 (0) | 0 (0) | -- | -- | -- | -- | -- | -- |
| *Adjusted for cluster, sex, age, and anthropometry at admission only performed for recovered, defaulted, and early discharge because the remainder of the outcomes registered less than 5% of cases in the 3 study groups. ** Not included cases with MUAC >125 and WHZ <-2 at admission nor those early discharged (MUAC < 125mm). Recovered: MUAC ≥ 125 mm. *** Not included cases with MUAC >115 and WHZ <-3 at admission nor those early discharged (MUAC < 125mm). Recovered: MUAC ≥ 125 mm. The significant results are in bold. CI: confidence interval; CMAM: community management of acute malnutrition model; iCCM: integrated community case management; SAM: severe acute malnutrition; MAM: moderate acute malnutrition. | | | | | | | | | |

| **Table S11.** Time to recovery, amount of ready-to use food used for treatment and anthropometric gain of recovered children compared by study group in the MUAC-only subsample (those admitted with MUAC < 125mm in all groups). | | | | | | | |
| --- | --- | --- | --- | --- | --- | --- | --- |
|  | **Control CMAM ^a^**  Median [IQR] | | **iCCM standard ^b^**  Median [IQR] | **iCCM simplified ^c^**  Median [IQR] | a vs. b  p-value | a vs. c  p-value | b vs. c  p-value |
| **WHOLE PROGRAM*** | |  |  |  |  |  |  |
| LOS (days) | 42.0 [35.0 – 49.0] | | 42.0 [31.0 – 68.0] | 43.0 [30.0 – 69.0] | 0.320 | 0.327 | 0.795 |
| RUTF sachets | 120.0 [100.0 – 150.0] | | 100.0 [75.0 – 120.0] | 49.0 [35.0 – 77.0] | **0.006** | **<0.001** | **<0.001** |
| RUSF sachets | 42.0 [28.0 – 56.0] | | 35.0 [28.0 – 42.0] | -- | 0.459 | -- | -- |
| Weight gain  (g/kg/day) | 4.75 [3.18 – 6.26] | | 3.81 [1.98 – 6.67] | 4.80 [2.85 – 7.14] | 0.317 | 0.562 | 0.603 |
| MUAC gain  (mm/day) | 0.36 [0.22 – 0.63] | | 0.24 [0.13 – 0.41] | 0.24 [0.13– 0.39] | 0.904 | 0.695 | 0.525 |
| **SAM**** |  | |  |  |  |  |  |
| LOS (days) | 42.0 [34.0 – 49.0] | | 42.0 [31.0 – 63.0] | 42.0 [35.0 – 63.0] | 0.481 | 0.437 | 0.638 |
| RUTF sachets | 120.0 [100.0 – 150.0] | | 100.0 [75.0 – 120.0] | 76.5 [70.0 – 91.0] | **0.006** | **<0.001** | **0.004** |
| Weight gain  (g/kg/day) | 5.70 [4.28 – 7.68] | | 5.19 [2.84 – 7.14] | 5.89 [3.49 – 7.42] | 0.430 | 0.664 | 0.702 |
| MUAC gain  (mm/day) | 0.36 [0.23 – 0.69] | | 0.34 [0.23 – 0.50] | 0.36 [0.24 – 0.47] | 0.324 | 0.409 | 0.859 |
| **MAM*** |  | |  |  |  |  |  |
| LOS (days) | 42.0 [35.0 – 50.0] | | 42.0 [33.0 – 77.0] | 48.0 [28.0 – 70.0] | 0.228 | 0.344 | 0.888 |
| RUTF sachets | -- | | -- | 35.0 [28.0 – 42.0] | -- | -- | -- |
| RUSF sachets | 42.0 [28.0 – 56.0] | | 35.0 [28.0 – 42.0] | -- | 0.459 | -- | -- |
| Weight gain  (g/kg/day) | 3.50 [2.48 – 4.51] | | 2.57 [1.31 – 4.80] | 3.17 [1.58 – 4.67] | 0.447 | 0.824 | 0.441 |
| MUAC gain  (mm/day) | 0.36 [0.19 – 0.55] | | 0.14 [0.08 – 0.28] | 0.14 [0.08 – 0.25] | 0.392 | 0.053 | 0.193 |
| * Not included cases with MUAC >125 and WHZ <-2 at admission nor those early discharged (MUAC < 125mm). Recovered: MUAC ≥ 125 mm.  ** Not included cases with MUAC >115 and WHZ <-3 at admission nor those early discharged (MUAC < 125mm). Recovered: MUAC ≥ 125 mm.  CMAM: community management of acute malnutrition; iCCM: integrated community case management; LOS: length of stay; MUAC: mid-upper arm circumference; MAM: moderate acute malnutrition; SAM: severe acute malnutrition; RUTF: Ready-to-use therapeutic food; RUSF: Ready-to-use supplementary food. In bold are the significant results. | | | | | | | |

| **Table S12.** Treatment results of children admitted with a weight under five kilograms. | | | | | | |
| --- | --- | --- | --- | --- | --- | --- |
| **TOTAL SAMPLE** | **Control CMAM ^a^**  N=25 | **iCCM standard ^b^**  N=59 | **iCCM simplified ^c^**  N=92 | b vs. a  Risk difference [95% C.I.]* | c vs. a  Risk difference [95% C.I.]* | c vs. b  Risk difference [95% C.I.]* |
| **Outcomes** | % (n) | % (n) | % (n) |  |  |  |
| Recovered | 76.0 (19) | 69.5 (41) | 91.3 (84) | -0.04 [-0.28 – 0.20]  p=0.370 | 0.12 [-0.9 – 0.54]  p=0.205 | **0.19 [0.06 – 0.36]**  **p=0.008** |
| Defaulted | 0 (0) | 8.5 (5) | 0 (0) | -- | -- | -- |
| Non-response | 0 (0) | 0 (0) | 3.3 (3) | -- | -- | -- |
| Referenced | 0 (0) | 3.4 (2) | 0 (0) | -- | -- | -- |
| Transferred | 0 (0) | 0 (0) | 0 (0) | -- | -- | -- |
| Death | 0 (0) | 0 (0) | 0 (0) | -- | -- | -- |
| Early discharge | 24.0 (6) | 18.6 (11) | 5.4 (5) | -0.11 [-0.37 – 0.10]  p=0.165 | -0.18 [-0.49 – 0.03]  p=0.090 | -0.09 [-0.20 – 0.02]  p=0.054 |
| **Of those recovered:** | Median [IQR] | Median [IQR] | Median [IQR] | a vs. b (p-value) | a vs. c (p-value) | b vs. c (p-value) |
| LOS (days) | 42.0 [32.0 – 53.0] | 59.5 [40.25 – 79.0] | 45.0 [32.0 – 69.0] | 0.232 | 0.564 | 0.427 |
| RUTF sachets | 100.0 [75.0 – 108.75] | 90.0 [60.0 – 120.0] | 56.0 [35.0 – 70.0] | 0.802 | **0.006** | **0.002** |
| RUSF sachets | 38.5 [33.25 – 42.0] | 42.0 [31.5 – 73.5] | -- | 0.436 | -- | -- |
| Weight gain  (g/kg/day) | 7.45 [5.95 – 10.12] | 5.98 [3.35 – 10.29] | 7.13 [4.29 – 8.18] | 0.306 | 0.487 | 0.564 |
| MUAC gain  (mm/day) | 0.29 [0.20 – 0.47] | 0.30 [0.20 – 0.48] | 0.25 [0.16 – 0.43] | 0.484 | 0.629 | 0.586 |
| *Adjusted for cluster, sex, age, and anthropometry at admission only performed for recovered and early discharge because the remainder of the outcomes registered less than 5% of cases in the 3 study groups. CMAM: community management of acute malnutrition; iCCM: integrated community case management; LOS: length of stay; MUAC: mid-upper arm circumference; MAM: moderate acute malnutrition; SAM: severe acute malnutrition; RUTF: Ready-to-use therapeutic food; RUSF: Ready-to-use supplementary food. In bold are the significant results. | | | | | | |

| **Table S13**. Treatment results compared by health care provider in the standard and simplified protocol groups for severe and moderate cases | | | | | | |
| --- | --- | --- | --- | --- | --- | --- |
| **TOTAL SAMPLE** | **iCCM Standard** | |  | **iCCM simplified** | |  |
|  | **CHWs**  Median [IQR] | **Nurses**  Median [IQR] | p-value | **CHWs**  Median [IQR] | **Nurses**  Median [IQR] | p-value |
| **TOTAL SAMPLE** |  |  |  |  |  |  |
| LOS (days) | 42.0 [31.0 – 65.0] | 42.0 [35.0 – 77.0] | 0.437 | 48.0 [28.0 – 77.0] | 41.0 [35.0 – 48.0] | 0.586 |
| RUTF sachets | 95.0 [70.5 – 120.0] | 100.0 [79.0 – 107.5] | 0.891 | 42.0 [33.5 – 64.75] | 84.0 [70.0 – 98.0] | **<0.001** |
| RUSF sachets | 28.0 [27.0 – 42.0] | 42.0 [35.0 – 42.0] | 0.090 | -- | -- | -- |
| Weight gain  (g/kg/day) | 3.87 [2.44 – 6.71] | 3.35 [1.45 – 6.49] | 0.191 | 3.37 [1.93 – 6.32] | 6.53 [5.10 – 7.64] | 0.576 |
| MUAC gain  (mm/day) | 0.24 [0.14 – 0.38] | 0.21 [0.10 – 0.43] | 0.512 | 0.20 [0.11 – 0.36] | 0.39 [0.33 – 0.48] | 0.292 |
| **SAM** |  |  |  |  |  |  |
| LOS (days) | 55.0 [35.0 – 70.0] | 35.0 [28.0 – 42.0] | 0.068 | 48.0 [33.5 – 84.0] | 41.0 [35.0 – 48.0] | 0.515 |
| RUTF sachets | 95.0 [70.5 – 120.0] | 100.0 [79.0 – 107.5] | 0.891 | 70.0 [56.0 – 84.0] | 84.0 [70.0 – 98.0] | **0.034** |
| RUSF sachets | -- | -- | -- | -- | -- | -- |
| Weight gain  (g/kg/day) | 4.61 [2.60 – 6.70] | 5.93 [3.90 – 7.35] | 0.374 | 3.97 [2.19 – 7.10] | 6.53 [5.10 – 7.64] | 0.995 |
| MUAC gain  (mm/day) | 0.28 [0.19 – 0.43] | 0.40 [0.29 – 0.57] | 0.266 | 0.33 [0.19 – 0.46] | 0.39 [0.33 – 0.48] | 0.253 |
| **MAM** |  |  |  |  |  |  |
| LOS (days) | 38.5 [28.0 – 56.0] | 77.0 [42.0 – 84.0] | 0.219 | 48.0 [28.0 – 70.0] | -- | -- |
| RUTF sachets | -- | -- | -- | 35.0 [28.0 – 42.0] | -- | -- |
| RUSF sachets | 28.0 [27.5 – 42.0] | 42.0 [35.0 – 42.0] | 0.090 | -- | -- | -- |
| Weight gain  (g/kg/day) | 3.57 [2.18 – 6.78] | 1.47 [0.92 – 2.00] | 0.495 | 3.16 [1.59 – 4.63] | -- | -- |
| MUAC gain  (mm/day) | 0.18 [0.10 – 0.34] | 0.08 [0.06 – 0.12] | 0.584 | 0.14 [0.08 – 0.25] | -- | -- |
| CHWs: community health workers; iCCM: integrated community case management; LOS: length of stay; MUAC: mid-upper arm circumference; MAM: moderate acute malnutrition; SAM: severe acute malnutrition; RUTF: Ready-to-use therapeutic food; RUSF: Ready-to-use supplementary food. In bold are the significant results. | | | | | | |
