# Supplementary material for: Effectiveness of decentralizing outpatient acute malnutrition treatment with community health workers and a simplified combined protocol: a cluster randomized controlled trial in emergency settings of Mali
Source: Front Public Health. 2024 Feb 21;12:1283148. doi: 10.3389/fpubh.2024.1283148 (PMC10915236; doi:10.3389/fpubh.2024.1283148)
Supplement: Supplementary file 2 [file Presentation_1.pdf]

ENQUETE DE COUVERTURE

NOM DU PROJET: « Efficacité, coût-efficacité et couverture du traitement de la malnutrition aigüe sévère délivré par les agents de santé communautaire à travers le protocole modifié dans des contextes d'urgence au Mali »

LOCALISATION: Région de Gao-Mali

'

DATE D'ENQUÊTE: Mars 2020

AUTEUR: Georges Alain Tchamba et Salimata Samake

TYPE D'ENQUÊTE: Couverture à grande échelle

TYPE DE PROGRAMME: MAS et MAM dans les CSCOM et dans la communauté

ORGANISATION EXECUTANTE: Action Contre la Faim

BAILLEUR : ELRHA et OFDA

TABLE DE MATIERE

LISTE DES TABLEAUX ii
LISTES DES FIGURES ii
REMERCIEMENTS iv
ACRONYMES v
RESUME vi
I. Contexte et justification 1
II. Objectifs 3
1. Objectif général 3
2. Objectifs spécifiques 3
III. METHODOLOGIE 4
1. Domaine couvert par l'enquête 4
2. Echantillonnage et plan de l'enquête 4
2.1. Population d'étude 4
2.2. Base de sondage 4
2.3. Taille de l'échantillon 4
2.4. Sélection des grappes 7
2.5. Sélection des ménages 8
2.6. Sélection des sujets 9
2.7. Procédure d'échantillonnage pour la recherche des raisons pour les cas couverts et non couverts 9
3. Préparation de la collecte des données 9
3.1. Analyse qualitative 9
3.2. Données anthropométriques 10
3.3. Estimation de la couverture avec intervalle de confiance à 95% 12
3.4. Ressources Humaines de l'enquête 16
3.5. Formation des enquêteurs 16
3.6. Standardisation des mesures 16
3.7. Traitement et analyse des données 18
4. Organisation sur le Terrain 18
5. Limites de l'enquête 19
6. Problèmes rencontrés 19
7. Considérations éthiques 20
IV. RESULTAT 21
1. Description de l'échantillon 21
2. Estimation de la couverture 23
3. Raison de la non couverture du programme 29
4. Raison de la couverture du programme 38
V. DISCUSSION 50
VI. RECOMMANDATIONS 55
ANNEXES 57

LISTE DES TABLEAUX

Tableau 1 Prévalence de la malnutrition à Gao, enquête SMART, 2019 5
Tableau 2 Calculs de la taille d'échantillon pour l'enquête sur la couverture des zones étendues, Gao, mars 2020 7
Tableau 3: Personnel et entretiens complétés pour l'investigation et étude communautaire – Gao, mars 2020 10
Tableau 4: Définitions des cas MAS et MAM dans les zones d'intervention et contrôle, mars 2020 11
Tableau 5: Définitions des cas MAS et MAM dans les zones d'intervention et contrôle, mars 2020 12
Tableau 6: Définitions des cas couverts et non couverts dans les zones d'intervention et contrôle, mars 2020 12
Tableau 7: Synthèse des données collectées, Gao, mars 2020 21
Tableau 8: Comparaison taille de l'échantillon attendue et atteinte dans les zones d'intervention et contrôle, mars 2020 21
Tableau 9: Nombre de cas couvert et non couvert pour le bras contrôle selon les critères d'admission, Gao mars 2020 23
Tableau 10: Nombre de cas couvert et non couvert pour le bras 1 selon les critères d'admission, Gao mars 2020 23
Tableau 11: Nombre de cas couvert et non couvert pour le bras 2 selon les critères d'admission, Gao mars 2020 24
Tableau 12: Couverture unique de la MAS et MAM pour chaque bras du projet, Gao, mars 2020 26
Tableau 13: Couverture de période pour le bras contrôle, Gao mars 2020 27
Tableau 14: Couverture de période pour le bras 1, Gao mars 2020 27
Tableau 15: Couverture de période pour le bras 2, Gao mars 2020 27
Tableau 16: Description des boosters, bras Contrôle: Gao mars 2020 43
Tableau 17: Description des boosters, Bras 1: Gao mars 2020 44
Tableau 18: Description des boosters, Bras 2: Gao mars 2020 45
Tableau 19 : Description des barrières, bras Contrôle ACF Gao,mars 2020 46
Tableau 20 : Description des barrières, Bras 1, ACF Gao,mars 2020 48
Tableau 21 : Description des barrières, Bras 2 ACF Gao,mars 2020 49

LISTES DES FIGURES

Figure 1 : Calculateur pour calculer la taille de l'échantillon dans les bras d'intervention et contrôle– ACF Gao mars 2020 6
Figure 2 : Raison de non accès aux soins de prise en charge de la MAM et MAS bras contrôle, mars 2020 30
Figure 3 : Raison de non accès aux soins de prise en charge de la MAM et MAS bras1, mars 2020 30
Figure 4 Raison de non accès aux soins de prise en charge de la MAM et MAS bras 2, mars 2020 31
Figure 5 Raison de non accès aux soins de prise en charge de la MAM et MAS bras 2, mars 2020 32

Figure 6 Lieu de traitement dans le bras contrôle, bras 1 et bras 2, mars 2020 33
Figure 7 Connaissance de la PCIMA dans le bras contrôle, bras 1 et bras 2, mars 2020 34
Figure 8 connaissance des maladies infantiles dans le bras contrôle, bras 1 et bras 2, mars 2020 34
Figure 9 Raison d'abandon du programme dans le bras contrôle, bras 1 et bras 2, mars 2020 35
Figure 10 Perception de la PCIMA dans le bras contrôle, bras 1 et bras 2, mars 2020 35
Figure 11 Prise de décision pour aller dans les structures de santé dans le bras contrôle, bras 1 et bras 2, mars 2020 37
Figure 12 Dépistage des enfants MAM et MAS, bras contrôle, 1 et 2, mars 2020 38
Figure 13 Booster dans le bras contrôle, mars 2020 40
Figure 14 Booster dans le bras 1 , mars 2020 40
Figure 15 Booster dans le bras 2, Gao mars 2020 41

REMERCIEMENTS

Nos remerciements sont adressés à toutes les personnes qui ont rendu possible la réalisation de cette évaluation : aux autorités administratives et sanitaires, au personnel des structures de santé, ainsi qu’aux communautés visitées pour leur collaboration et participation active.
Merci également à toute l’équipe d’Action Contre la Faim qui a rendu cette évaluation possible grâce à l’appui financier, aux Districts pour leur participation active, constructive et enthousiaste tout au long de l’évaluation, ainsi qu’aux enquêteurs, enquêtrices et superviseurs pour la qualité de leur travail et leur motivation, sans qui ce travail n’aurait pu être réalisé.

L’évaluation a été menée par l’équipe d’Action Contre la Faim Mali

ACRONYMES

ACF	Action Contre la Faim-
AS	Agent de Santé
ATPE	Aliment Thérapeutique prêt à l'Emploi
CSCOM :	Centre de santé communautaire
DS	District Sanitaire
ELRHA :	Enhancing Learning and Research for Humanitarian Assistance.
MAG	Malnutrition Aigüe Globale
MAS	Malnutrition Aigüe Sévère
ODK :	Open Data Kit
OFDA:	Office for US Foreign Disaster Assistance
OMS	Organisation Mondiale de la Santé
ONG	Organisation Non Gouvernementale
P/T	Poids/Taille
PAM	Programme Alimentaire Mondial
PB	Périmètre Brachial
PCIMA	Prise en Charge Intégrée de la Malnutrition Aigue
RECO	Relais Communautaire
SMART	Standardized Monitoring and assessment of Relief and Transition

RESUME

Cette évaluation de la couverture a été menée dans le bras contrôle (protocole standard au centre de santé), bras 1 (protocole standard au centre de santé et site ASC) et bras 2 (protocole simplifié au centre de santé et site ASC) dans le district de Gao au Mali. Ces 3 bras ont été tirés par randomisation parmi les villages de l'ensemble du district dans le cadre de l'évaluation de base « Baseline » du projet ICCM+ (Integrated Community Case Management) financé par OFDA et ELRHA. Ce projet a pour but de décentraliser la prise en charge de la malnutrition aigüe au niveau de la communauté en s'appuyant sur les agents de santé communautaire (ASC) et de voir s'il y'a une augmentation de la couverture de la prise en charge des MAS dans le bras 1 et bras 2. Cette enquête de couverture a été réalisée du 20 février au 19 mars 2020 et a pour objectifs d'estimer la couverture unique, les barrières et les boosters dans les bras contrôle, bras 1 et bras 2.

L'enquête de couverture a été réalisée dans le bras contrôle et le bras d'intervention (bras 1 et bras2) en utilisant une méthodologie adaptée « d'enquête sur une grande zone ». La taille de l'échantillon a été calculée à partir du calculateur Sampsize[1] indiqué pour ce type d'enquête. Les villages ont été sélectionnés par la méthode de tirage aléatoire systématique pour chaque bras. Les enfants étaient identifiés par la stratégie porte à porte où tous les enfants étaient mesurés dans chaque ménage. Lorsque le périmètre brachial était supérieur à 140 mm en absence d'œdème, les mesures anthropométriques n'étaient plus prises. La couverture unique de la malnutrition aigüe sévère (MAS) et de la malnutrition aigüe modéré (MAM) chez les enfants âgés de 6 à 59 mois a été mesurée à l'aide de trois indicateurs : périmètre brachial, indice Poids-Taille et œdème. L'indice Poids-Taille a été calculé à partir du logiciel ENA-delta version janvier 2020 et Excel. Les indices nutritionnels ont été calculés en utilisant la population de référence OMS (2006). Une enquête qualitative a été réalisée auprès des cibles précises et des méthodologies d'entretien semi structuré et de focus group dans chaque bras ont été utilisées.

Les tailles d'échantillon pour MAM et MAS ont été atteintes dans les trois bras de l'étude. Les estimations de couverture sont les suivantes:

	BRAS CONTROLE		BRAS 1		BRAS 2	
	**MAS**	**MAM**	**MAS**	**MAM**	**MAS**	**MAM**
**Couverture unique**	20,5%	9,8%	25,0%	13,9%	6,3%	12,1%
**Intervalle de confiance**	12,0%-28,9%	6,4%-13,2%	13,7%-36,3%	8,2%-19,5%	1,4%-11,2%	6,8%-17,3%

Les résultats de l'enquête montrent une couverture unique des MAS, MAM largement en dessous de 50% qui est le seuil recommandé par les normes sphères en zone rurale. Cette faible couverture est due à un manque des moyens financiers pour le déplacement, la rupture de stock et un faible dépistage actif. Cette couverture est en générale plus faible pour les MAM à l'exception du bras 2. Ce faible taux comparativement entre les MAM et MAS du bras 2 est à pondérer avec notamment la recrudescence de l'insécurité dans la zone limitant les déplacements de la communauté vers les CSCOMS et sa vulnérabilité géographique. De plus, les villages éloignés ou

[1] http://sampsize.sourceforge.net/iface/index.html#prev

qui se trouvent isolés sont les endroits où la couverture est plus faible à cause du fait que la plupart des villages où se trouvent 70% des MAS sont très éloignés(>15km) des CSCOM alors que ceux des MAM sont plus proches des CSCOM(<5km). La couverture est plus faible dans les bras 2 par rapport aux bras 1 et contrôle principalement à cause de l'éloignement de la plupart des villages des lieux des soins. Ces résultats sont représentatifs des bras contrôle, bras 1 et bras 2 et non des du district de Gao.

Toutefois, les résultats indiquent que, dans certaines zones des trois bras de l'étude :

Les activités communautaires (dépistage actif) sont limitées avec en moyenne 85,9% des MAM et MAS non couverts n'ont pas été dépistés le mois antérieur à l'enquête. En analysant les données des enfants MAM et MAS non couverts par rapport au critères d'admission versus critères de dépistage, on observe qu'une proportion assez importante des enfants MAM et MAS ne sont ni identifiés par le PB/œdème dans la communauté et encore moins avec le Poids-Taille dans le CSCOM. En somme, le système de dépistage communautaire et de triage au niveau des CSCOM ne permettent pas d'identifier l'ensemble de tous les enfants malnutris dans la communauté. De plus, les réunions sont irrégulières entre le CSCOM et les relais communautaires. De plus, le retard de l'arrivée des patients s'expliquerait aussi par le fait que la décision d'aller au CSCOM est prise par 79,9% des pères.

- Les ruptures de stock au niveau du CSCOM avec en moyenne 54,3% des cas MAM non couverts et 50,3 %des cas MAS non couverts ont évoqué le problème de rupture de stock
- La distance/moyen financier pour le déplacement est un obstacle non négligeable car au-delà de 8,5 km la plupart des patients non couverts n'allaient pas au CSCOM.

Ces barrières entravent la prestation des services de la PCIMA (prise en charge intégrée de la malnutrition aigüe) et seulement une faible partie des accompagnants préfèrent toujours se faire soigner dans les CSCOM.

En ce qui concerne d'autres raisons de la non-participation au traitement, il n'y avait pas de différences majeures entre les bras contrôle et d'intervention (bras1 et bras 2). On note aussi que dans les trois bras, en moyenne 45,3% des mères d'enfant ne connaissant pas la PCIMA. Ces raisons sont également des indicateurs d'un manque de compréhension des membres de la communauté à l'égard des la PCIMA, ce qui ajoute à la preuve du manque d'activités de proximité menées par les relais communautaires dans certaines communautés.

Les personnes qui s'occupent d'enfants malades semblent préférer pour 46,8% les traitement dans la communauté (tradipraticien et automedication) Le fait d'aller de se traiter dans la communauté serait probablement du au manque de moyen financier pour le déplacement et la recrudescence de l'insécurité dans la zone limitant les déplacements de la communauté vers les CSCOMS. Mais cela indique que même si les distances entre les communautés et les centres de santé communautaires les plus proches sont grandes, certains accompagnants semblent disposés à se rendre aux centres de santé communautaires sous la recommandation du relais communautaire. Par la suite, en cas de non amélioration de l'état de santé de l'enfant, les familles se rendent aux centres de santé communautaires pour 31% des accompagnants quelque soit le bras. Les personnels de santé ont confirmé qu'une très grande majorité des enfants malnutris a déjà reçu à domicile d'autres traitements avant leur admission dans le programme. Cette attitude crée un retard de traitement

Même si l'analyse des données quantitatives et qualitatives ont relevé plusieurs facteurs négatifs en rapport avec l'organisation de la PCIMA dans ces trois bras, des facteurs positifs ci-dessous permettent d'avoir les couvertures estimées. Il s'agît du fait que les infirmiers diagnostiques correctement la maladie de l'enfant et les encouragements des mamans par les relais communautaires d'aller au CSCOM. A tout ceci, la gratuité joue aussi un rôle non négligeable favorisant l'accessibilité aux soins. En moyenne 72,5% des accompagnants ont une perception de la PCIMA comme un endroit où on traite les enfants malnutris correctement. Les sensibilisations communautaires, la connaissance des maladies infantiles (85,7% des accompagnants) et la qualité des services ont joué un rôle non négligeable dans les couvertures uniques obtenues.

Recommandations

Suite aux résultats de l'évaluation de la couverture, présentés dans le présent rapport, les recommandations suivantes ont été élaborées afin d'adresser les principaux facteurs de blocage identifiés et ainsi améliorer la couverture du programme PCIMA dans le projet ICCM+.

- Faire un suivi des principales barrières durant le projet
- Mettre en place une stratégie communautaire (stratégie PB ménage, redynamisation des relais communautaires par village selon la densité de la population communautaire) pour mieux identifier les enfants malnutris et faire leur suivi
- Mettre en place une stratégie de sensibilisation et de mobilisation communautaire dans les différents villages des trois bras en impliquant les chefs de villages et les autorités religieuses
- Mettre en place une stratégie mobile et avancée dans les bras 1 et 2 afin d'atteindre les enfants situés à une grande distance des structures sanitaires et des villages
- Faire un plaidoyer pour un ravitaillement des intrants dans les CSCOM en tenant compte du stock tampon
- Trouver un moyen approprié pour le ravitaillement des structures sanitaires à partir du district

I. Contexte et justification

D'après les dernières estimations, 16,6 millions d'enfants de moins de cinq ans souffrent de malnutrition aigüe sévère (MAS) dans le monde, ce qui fait de la malnutrition un problème majeur de santé publiquei. Des changements significatifs sont survenus au cours des deux dernières décennies concernant la prise en charge de la MAS, passant d'un traitement hospitalier à un traitement ambulatoire grâce au développement des Aliments Thérapeutiques Prêts à l'Emploi (ATPE) et au protocole de Prise en charge Intégrée de la Malnutrition aigüe (PCIMA).
Les services de santé publique ont cherché à rendre les interventions clés pour la survie des enfants plus intégrées et plus accessibles. L'approche Prise en Charge intégrée des Maladies de l'enfant - Communautaires (PCIME-C) ou ICCM (Integrated Community Case Management) a été introduite afin d'améliorer le recours aux soins dans des zones où l'accessibilité aux structures sanitaire est difficile. Cette approche est basée sur la formation des agents de santé communautaire (ASC) afin de fournir des services de traitement spécifiques pour des maladies infectieuses entraînant une forte mortalité. La preuve de l'efficacité des ASC dans le traitement de la MAS a été synthétisée dans une revue récente publiée par Action Contre la Faim et ses partenaires. Cependant, il y a très peu des données relatives au traitement de la MAS par les ASC dans des situations d'urgence.
Le Nord du Mali est classé comme « crise oubliée » et au rang de « Risque élevé » dans l'indice de gestion des risques INFORM 2019 (16ème sur 191 pays). Cela s'explique par la détérioration de la situation sécuritaire, la difficulté d'accès aux services sociaux de base et l'exposition aux dangers climatiques. La situation humanitaire complexe, exacerbée par l'insécurité, la crise politique et les conflits intercommunautaires, contribue considérablement à la vulnérabilité de la population. En mars 2018, le Cadre intégré de classification par phases de la sécurité alimentaire (IPC) a estimé que 15 % de la population entrerait dans une phase d'insécurité alimentaire (Phase 2) et 8 % dans une phase de crise (Phase 3). En 2019, il était attendu une dégradation de cette situation. A Gao la prévalence de la malnutrition aigüe était la plus élevée au niveau du pays : 3,1 % [1,9-4,8] pour la MAS et 14,2 % [11,6-17,3] pour la Malnutrition Aigüe Globale (MAG). Ce taux de MAS dépasse le seuil d'urgence de l'OMS fixé à (2 %).

Partant de ce constat, Action Contre la Faim et ses partenaires ont élaboré un projet de recherche concernant le district sanitaire de Gao intitulé « Efficacité, coût-efficacité et couverture du traitement de la malnutrition aigüe sévère délivré par les agents de santé communautaire à travers le protocole modifié dans des contextes d'urgence au Mali ».
Cette recherche proposée est basée sur l'expérience préalable d'Action contre la Faim (ACF) en partenariat avec le Ministère de la Santé (MS), l'Institut National de Recherche et Santé Publique (INSP) du Mali et la Fondation Innocent dans la région de Kayes au sud (zone plus stable). Avant la mise en place de ce projet, une enquête de couverture du programme de PECMAS a été réalisée.

Les trois bras du projet ICCM+ ont été choisis selon les critères ci-dessous.

Paramètre	Bras contrôle	Bras 1	Bras 2
**Type de malnutrition**	MAS et MAM	MAS et MAM	MAS et MAM
**Niveau pyramide sanitaire**	Centre de santé communautaire	Centre de santé communautaire et site ASC	Centre de santé communautaire et site ASC
**Produit utilisé**	Plumpy nut pour les MAS et plumpy sup/farine enrichie pour les MAM	Plumpy nut pour les MAS et plumpy sup/farine enrichie pour les MAM	Plumpy nut pour les MAM et MAS
**Type de protocole**	Protocole standard	Protocole standard	Protocole simplifié
**Administration de la posologie**	En fonction du poids de l'enfant	En fonction du poids de l'enfant	Posologie fixe et ne tient pas compte du poids de l'enfant
**Critère d'admission et de sortie**	**Admission**<br>PT<-3z score ou PB<115mm<br>**Sortie**<br>PT>-1,5 z score et PB>125 mm	**Admission**<br>PT<-3z score ou PB<115mm<br>**Sortie**<br>PT>-1,5 z score et PB>125 mm	**Admission**<br>PB<115mm<br>**Sortie**<br>PB>125 mm

II. Objectifs

1.Objectif général

L’enquête de couverture détaillée dans le présent rapport a été réalisée en tant qu’enquête Baseline de couverture pour l’étude de recherche, le principal objectif étant d’évaluer la couverture des traitements MAS et MAM chez les enfants âgés de 6 à 59 mois dans les bras d’intervention (bras 1 et bras 2) et le bras contrôle de l’étude.

2. Objectifs spécifiques

La méthodologie de l’enquête visait également à évaluer la couverture de la MAS et MAM chez les enfants âgés de 6 à 59 mois. Par conséquent, les objectifs de l'enquête dans les bras d'intervention et de contrôle de l'étude dans les trois districts étaient :

- Estimer la couverture unique des MAM et MAS du programme des trois bras du projet;
- Identifier et analyser les facteurs de blocage à l’accès et la couverture des soins dans les trois bras
- Identifier et analyser les facteurs facilitant l’accès et la couverture dans les trois bras du projet ;

III. METHODOLOGIE

Dans cette partie relative à la méthodologie de l'enquête, nous présenterons dans un premier temps la procédure utilisée pour l'échantillonnage, puis nous aborderons les aspects relatifs à la collecte des données, incluant le questionnaire, les équipements, le personnel et l'organisation pratique, avant de terminer par le traitement et l'analyse des données.

1.Domaine couvert par l'enquête

Il s'agit d'une enquête transversale sur un échantillon représentatif des ménages dans le bras contrôle, bras 1 et bras 2.

2.Echantillonnage et plan de l'enquête

2.1. Population d'étude

L'enquête a concerné les ménages présents au moment de l'enquête et vivant dans le bras contrôle, bras 1 et bras 2. Dans les maisons, tous les enfants âgés de 6 à 59 mois ont été inclus dans l'enquête.

2.2. Base de sondage

La base de sondage utilisée pour l'échantillonnage était constituée à partir des listes de villages par aire de santé du bras contrôle, bras 1 et bras 2. Les chiffres de population sont ceux fournis par la région médicale pour les districts de Gao (population actualisée pour l'année 2020).

2.3. Taille de l'échantillon

Dans le cadre des enquêtes de couverture SLEAC et SQUEAC, les couvertures uniques sont calculées avec une petite taille d'échantillon. Pour pallier à ce problème de taille d'échantillon, un échantillon suffisamment grand des cas dans cette enquête de couverture est indiqué pour estimer une couverture dans une zone avec plus de précision[2]. Par conséquent, à la fois dans les bras contrôle et d'intervention (bras 1 et bras 2) ACF a mené 3 enquêtes distinctes à grande échelle portant sur les indicateurs précis afin d'identifier les niveaux des couvertures pour chaque bras. Les résultats de cette enquête ont ensuite été analysés pour estimer la couverture du traitement MAS et MAM avec un intervalle de confiance de 95%.

[2] Plus d'informations à la page 127 du manuel technique SQUEAC et SLEAC(36)

Le calculateur Sampsize indiqué pour ce type d'enquête a été utilisé pour calculer la taille d'échantillon requise pour les maladies dans la communauté[3]. Pour ce faire, les données suivantes doivent être ajoutées à la calculatrice:

- **Précision:**

La précision souhaitée de l'estimation finale. Une précision de 10 à 15% est acceptable pour les estimations de la couverture. Dans le cadre de cette enquête la précision de 10% a été utilisée dans la mesure que les zones d'intervention et contrôle sont moins étendues.

- **Prévalence**:

La couverture de traitement estimée de l'intervention (si cela n'est pas connu, il convient d'utiliser 50%). Pour cette enquête, 50% a été utilisé sur la base des estimations de la couverture de base.

- **Niveau:**

Le niveau souhaité de l'intervalle de confiance 95% est utilisé pour les enquêtes de couverture.

- **Population:**

L'estimation de la population souffrant de la malnutrition dans les zones d'enquête a été calculée selon les détails au tableau 2 ci-dessous

Les populations attendues pour la malnutrition aigüe ont été calculées sur la base des estimations de prévalence les plus récentes et les plus précises, ainsi que sur les populations d'enfants âgés de 6 à 59 mois pour la malnutrition aigüe (MAS, MAM) dans la zone de l'enquête. La formule suivante a été utilisée (n = population estimée de cas MAM/MAS dans chaque unité de prestation de services):

$$n = pop.moyenne\ par\ village_{tous\ les\ ages} \times \frac{\%\ de\ la\ population_{6-59\ mois}}{100} \times \frac{prévalence\ MAS}{100}$$

Tableau 1 Prévalence de la malnutrition à Gao, enquête SMART, 2019

Régions/ Départements	N	Z–score <–2 et/ou œdèmes (IC_{95})	<-2 Z-score et >=-3 Z-score pas d'oedèmes (IC_{95})	Z–score <–3 et/ou œdèmes (IC_{95})

[333] http://sampsize.sourceforge.net/iface/index.html#prev

**Gao**	1053	11,6[9,3-14,3]	9,8 [7,8-12,3]	1,8[1,1- 3,0]

- **Prévalence des MAM et MAS:**

La prévalence MAS, basée sur les estimations PT tirées de l'enquête SMART de juillet 2019 a été utilisé car la taille de l'échantillon serait acceptable pour permettre une mise en œuvre réaliste de l'enquête. L'enquête SMART a été faite en début de la période de soudure (juillet), période de l'année quand la prévalence devrait être la plus haute. Par contre, l'enquête de couverture s'est déroulée en mars 2020, à la fin de la période de d'abondance. Bien qu'il est bien connu que les enquêtes SMART dans les zones rurale sous-estiment la prévalence[4], on pense qu'une approximation plus conservative est plus appropriée. Les estimations ont donc été faites avec le niveau plus bas de l'intervalle de confiance **(MAS=1,1%)** et **MAM=7,8%)**. Ce choix a tenu compte de la logistique réaliste pour la taille de l'échantillon.

- ***Population moyenne par village*** **tous âges:**

$$Population\ moyenne\ par\ village = \frac{Population\ totale\ du\ DS}{Nombre\ total\ de\ villages\ et\ ou\ communs}$$

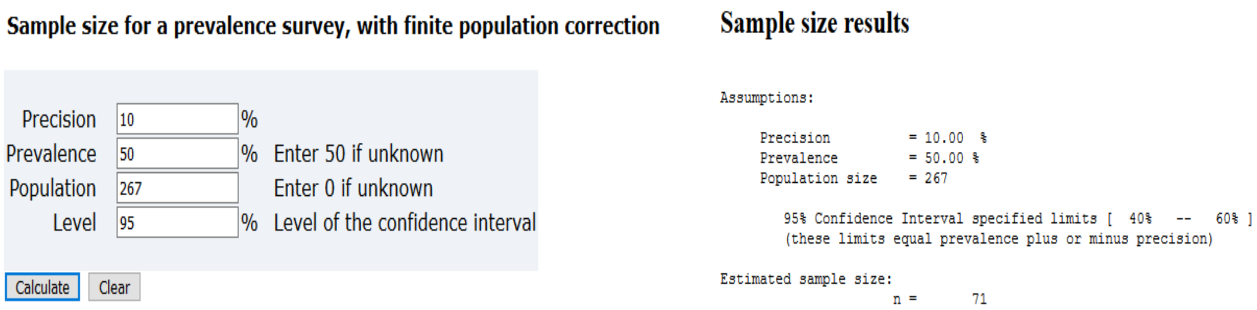

Figure 1 : Calculateur pour calculer la taille de l'échantillon dans les bras d'intervention et contrôle– ACF Gao mars 2020

[4] Crowe et al. (2014), Effect of nutrition survey 'cleaning criteria' on estimates of malnutrition prevalence and disease burden: secondary data analysis. PeerJ 2:e380; DOI 10.7717/peerj.380

Tableau 2 Calculs de la taille d'échantillon pour l'enquête sur la couverture des zones étendues, Gao, mars 2020

Paramètres	Control	Bras 1	Bras 2	Total
Population totale	81 057	45 708	55 884	182 649
Nombre de villages totale	62	33	60	155
Population moyenne par village (tous âges)	1 307	1 385	931	3 624
% d'enfants de moins de 5 ans	18,00%	18,00%	18,00%	
Population d'enfant âgé de 6-59m estimée	14 590	8 227	10 059	32 877
Prévalence par P/T MAG	9,30%			
Population MAG estimé	1 357	765	935	3 058
Taille d'échantillon pour estimer la couverture MAG	**90**	**86**	**88**	264
Nombre de village à visiter MAG	4	4	6	13
Village additionnel MAG	4	4	4	12
Nombre de village définitif à visiter MAG	8	8	10	25
Prévalence par P/T MAM	7,80%			
Population MAM estimé	1138	642	785	2 564
Taille d'échantillon pour estimer la couverture MAM	**89**	**84**	**86**	259
Nombre de village à visiter MAM	5	4	7	16
Village additionnel MAM	4	4	4	12
Nombre de village définitif à visiter MAM	9	8	11	28
Prévalence par P/T MAS	1,10%			
Population MAS estimé	160	91	111	362
Taille d'échantillon pour estimer la couverture	**61**	**47**	**52**	160
Nombre de village à visiter MAS	24	17	28	69
Village additionnel MAS	4	4	4	12
Nombre total de village définitif à visiter MAS	**28**	**21**	**32**	81

2.4. Sélection des grappes

➢ **Calcul du nombre de villages à visiter**

Sur la base des tailles d'échantillons requises, l'étape suivante consistait à calculer le nombre requis de villages ($n_{village}$) à visiter pour atteindre les tailles d'échantillons requises. Étant donné que les équipes se rendaient dans chaque village et effectuaient la recherche de cas, la taille de l'échantillon de la malnutrition la plus rare a été utilisée pour calculer le nombre de villages à visiter.
La taille de l'échantillon a été utilisée pour définir le nombre de villages ayant besoin d'en faire partie avec l'équation

$$n_{villages} = \left\lceil \frac{n}{population\ moyenne\ par\ village_{tous\ les\ âges} \times \frac{pourcentage\ de\ la\ population_{6-59\ mois}}{100} \times \frac{prévalence\ MAS}{100}} \right\rceil$$

Le tableau 2 montre les données utilisées pour calculer le nombre de village à visiter pour avoir une taille minimum de l'échantillon des cas MAM. Il indique qu'il serait nécessaire de visiter un nombre beaucoup plus réduit de village afin d'atteindre la taille minimale de l'échantillon pour les cas MAM. Cependant, la recherche des cas MAS et MAM seront identifiés dans le même village d'où le nombre de village à visiter sera basé sur l'échantillon de la MAS qui est la plus grande taille.

Au cours de la collecte de données dans le bras contrôle, 1 et 2, 4 villages de plus[5] ont été visités pour dépister des cas, combler l'insuffisance de la fiabilité des données démographiques, les villages vides ou inexistants et se donner le maximum de chance pour retrouver les différents cas.

- **Sélection des villages**

Pour la sélection des grappes, nous avons considéré les villages/quartier comme la plus petite unité administrative pouvant abriter les grappes. La sélection aléatoire des villages a été faite au moyen de la méthode d'échantillonnage aléatoire stratifié systématique pour assurer une représentativité spatiale en utilisant une liste de villages par poste de santé et case de santé. Cette méthode de tirage a été utilisée dans la mesure qu'il n'y a aucune carte détaillée/complète disponible. La liste des villages a été organisée par ordre alphabétique par aire de santé et par village et ils ont été numérotées. Un pas d´échantillonnage a été calculé et appliqué pour la sélection des villages respectivement pour la zone d'intervention (bras 1 et 2) et contrôle dans les CSCOM. Le pas de sondage peut être calculé avec la formule suivante :

$$Pas\ de\ sondage = \frac{Nombre\ total\ de\ villages\ et\ ou\ communs}{Nombre\ de\ villages\ à\ visiter}$$

Pour choisir le premier village, les équipes ont sélectionné un numéro aléatoire entre 1 et le pas de sondage en utilisant Excel. Ensuite elles sont passées au village suivant en fonction du pas de sondage. Par la suite, on a ajouté ainsi le pas de sondage jusqu'à la fin des listes des villages. Enfin, les villages ont été planifiés par les enquêteurs et superviseurs pour la recherche des cas dans les villages. La liste complète des villages est à l'annexe 2

2.5. Sélection des ménages

[5] Lors des enquêtes de couverture, il est conseillé d'ajouter des villages supplémentaires pour donner aux équipes d'enquête une meilleure chance d'atteindre la taille d'échantillon cible requise.

Seuls les ménages ordinaires ont été concernés pour cette enquête. Autrement dit, pour cette enquête, ont été exclus les couvents, les orphelinats, les hommes, des vieillards, les prisons et les permanences des mosquées. Une fois arrivée dans le village/quartier d'enquête, les enquêteurs cherchaient à enquêter tous les ménages dans le village/quartier.
Les équipes ont procédé à un échantillonnage porte à porte afin d'identifier les « cas». L'échantillonnage porte à porte est la méthode d'échantillonnage recommandée à utiliser pour tenter d'identifier les cas de MAM.

2.6. Sélection des sujets

- **Définition de cas:**

Dans le cadre de cette enquête, un cas inclut tout enfant de 6 à 59 mois de la population MAS ou MAM au moment de l'enquête et / ou tout enfant inscrit dans une structure sanitaire de traitement de la MAS ou MAM au moment de l'enquête.

- **Méthode d'identification des cibles :**

Une fois les villages sélectionnés, les équipes d'enquête se sont rendues dans chaque village pour dépistage exhaustif dans la communauté de tous les cas éligibles afin d'enregistrer leurs données anthropométriques et déterminer s'ils sont inscrits ou non au programme de traitement concerné.

Dans chaque ménage, tous les enfants âgés de 6 à 59 mois, s'y trouvant était tous inclus pour les mesures anthropométriques (mesure de poids, taille, œdèmes et périmètre brachial).
Si les occupants d'une maison n'étaient pas présents, les enquêteurs revenaient visiter la maison avant la fin de la journée.

2.7. Procédure d'échantillonnage pour la recherche des raisons pour les cas couverts et non couverts

Les données qualitatives collectées auprès des accompagnants des cas non couverts ou non couverts identifiés ont été analysées pour identifier et classer les raisons de la non-participation au traitement (obstacles). Cela a été fait en analysant les résultats dans la base de données «ODK Collect» de l'enquête.

3.Préparation de la collecte des données

3.1.Analyse qualitative

La collecte des données qualitatives a été organisée selon la « matrice d'échantillonnage qualitative » développée en collaboration avec les membres clés de l'équipe d''investigation. Pour chaque CSCOM, un village a été sélectionné prêt du CSCOM et un autre à plus de 10km du CSCOM. Sur le plan journalier, chaque équipe, composée d'un superviseur et de deux enquêteurs, a réalisé en moyenne deux entretiens semi-directifs et/ou discussions en groupe, arrivant au total de 105 entretiens (74 entretiens semi directs, 21 focus group et 10 études des cas) sur l'ensemble de la zone d'intervention et contrôle.

Les différentes personnes rencontrées et les différentes méthodes utilisées ont permis de collecter les informations concernant les barrières et les boosters relatifs à la PCIMA. Ces données ont été répertoriées de façon journalière avec l'équipe d'enquête en utilisant l´outil BBQ: Barrières, Boosters et Questions. Cet outil permet non seulement d´organiser l´information jour à jour pour continuer avec la recherche de façon interactive et dirigée, mais aussi pour assurer la triangulation des informations. Afin d´assurer l´exhaustivité du processus, la recherche d´information a continué jusqu'à la saturation (- jusqu´à ce que la même information revienne des différentes sources et par différentes méthodes).

Tableau 3*:* Personnel et entretiens complétés pour l'investigation et étude communautaire – Gao, mars 2020

			**Nombre d'Entretiens Complétés**			
**Code**	**Cible**	**Type d'Entretien**	**Bras contrôle**	**Bras 1**	**Bras 2**	**Total**
**F**	Femmes de la communauté	Entretiens semi-structurée	5	3	2	10
		Discussion de groupe	6	3	2	11
**H**	Hommes de la communauté	Entretiens semi-structurée	5	3	2	10
		Discussion de groupe	5	3	2	10
**A$^{MAS\ F}$**	Accompagnante MAS (femme)	Entretiens semi-structurée	5	3	3	11
**GT**	Guérisseur traditionnel/ Accoucheuse traditionnelle	Entretiens semi-structurée	4	3	2	9
**RC**	Relais communautaire	Entretiens semi-structurée	5	3	3	11
**IM**	IMAM	Entretiens semi-structurée	4	3	2	9
**CV**	Les leaders communautaires	Entretiens semi-structurée	8	5	2	15
**IT**	Infirmier titulaire	Entretiens semi-structurée	4	3	2	9

**Code**	**Méthode**
1	**FG=** Discussion de groupe semi-directive
2	**ESS=** Entretien semi-directif
3	**Quantitatif**

3.2.Données anthropométriques

- **Vérification de l'âge**:

Pour déterminer l'âge des enfants identifiés, les équipes de l'enquête ont d'abord demandé si l'accompagnant peut leur montrer un acte de naissance, une carte d'identité, une carte PEV ou une carte de traitement du programme de nutrition. Si l'accompagnant ne pouvait fournir aucun de ceux-ci et ne pouvait pas donner d'âge exact pour l'enfant, l'équipe d'enquête a utilisé un calendrier des événements clés locaux pour déterminer l'âge de l'enfant.

- **Identification des cas MAS et MAM**:

Les cas MAS et MAM ont été identifiés par MUAC, présence d'œdème et / ou par le Z score du poids pour la taille. Lors d'une évaluation de la couverture, les cas sont généralement identifiés par les équipes de collecte de données sur la base du protocole d'identification et de référence des cas de malnutrition au Mali.

Cependant, pour cette enquête de couverture nous avons décidé de calculer les Z scores de tous les enfants dont le PB était inférieur à 140 mm. Ce seuil a été déterminé à base de l'analyse des données mensuelles des enfants pris en charge dans le programme qui montrent que les cas MAS et MAM ont été identifiés avec des mesures de PB supérieures à 125 mm mais ces enfants ont été classés comme MAS ou MAM par Z-score. Le z-score a été calculé automatiquement par le logiciel NutriSurvey.ena delta version de janvier 2020 placé sur l'une des trois tablettes de chaque équipe. Par la suite, l'équipe d'enquête a classé chaque enfant dans la catégorie MAS, MAM ou bien nourris.

Les définitions de cas pour l'identification des cas MAS et MAM lors de l'évaluation de couverture sont résumées dans le tableau 3 ci-après.

Tableau 4: Définitions des cas MAS et MAM dans les zones d'intervention et contrôle, mars 2020

**Description**	**Périmètre brachial**	**Œdème**	**Z-score**
**Malnutrition aigüe sévère**	<115 mm	+, ++, +++	<-3
**Malnutrition aigüe modéré**	115-124mm	Pas d'œdème	-2 et -3

- **Confirmation de l'inscription dans un programme:**

Les enfants inscrits dans une structure sanitaire pour prise en charge de la MAM ou MAS devraient remplir les critères ci-dessous.

Tableau 5*:* Définitions des cas MAS et MAM dans les zones d'intervention et contrôle, mars 2020

<table>
<tr><th>Traitement MAS</th><th>Traitement MAM</th></tr>
<tr><td>Possède les plumpy nut
ou
confirmation par un relais communautaire</td><td>Carte d'identification
Ou
Plumpy sup ou farine enrichie
Ou
Confirmation par le relais communautaire</td></tr>
<tr><td colspan="2">S'il n'était pas possible de confirmer que l'enfant participait au programme concerné, alors il était considéré comme un cas non couvert.</td></tr>
</table>

- **Classification des cas identifiés pendant l'enquête**

Tableau 6*:* Définitions des cas couverts et non couverts dans les zones d'intervention et contrôle, mars 2020

	**Malnutrition aigüe (MAS/MAM)**
**Cas couvert** (C_{in})	il s'agit d'un enfant souffrant de malnutrition aiguë au moment de l'enquête, mais qui suit un traitement
**Cas non couvert**(C_{out})	il s'agit d'un enfant souffrant de malnutrition aiguë au moment de l'enquête et qui ne reçoit pas de traitement.
**Cas en voie de guérison**(R_{in})	il s'agit d'un enfant qui n'est pas malade au moment de l'enquête, mais qui continue de recevoir un traitement car il n'a pas encore atteint les critères de sortie.

3.3.Estimation de la couverture avec intervalle de confiance à 95%

Une fois l'enquête terminée dans les villages sélectionnés, les équipes d'enquête ont communiqué les totaux de chaque classification de cas à l'équipe de coordination de l'enquête. Lorsque la taille des échantillons était atteinte ou dépassée, il était alors possible d'estimer la couverture. Pour la couverture de MAM et MAS, l'estimateur de couverture

unique[6] est l'estimateur recommandé. En effet, l'estimateur de la couverture unique est un estimateur qui remplace les deux estimateurs précédents : couverture actuelle et période. L'estimateur unique est un estimateur de couverture développé depuis 2015 qui est conseillé pour tous les contextes

Pour la crédibilité et sa validité statistique, la couverture unique doit être calculée en ajoutant un nouvel élément: les cas en voie de guérison hors du programme, ou cas en voie de guérison spontanée. Cet élément ne peut pas être calculé lors des enquêtes. En revanche, une équation a été développée pour estimer cette valeur en fonction des données disponibles des cas MAS/MAM couverts, cas MAS/MAM non couverts et cas en voie de guérison dans le programme. Dans le cadre de la présente investigation. Le calcul de la couverture unique utilise les données de l'enquête selon la formule suivante :

$$\text{Couverture unique} = \frac{C_{in} + R_{in}}{C_{in} + R_{in} + C_{out} + R_{out}}$$

C_{in} : Nombre de cas MAS couvert dans le programme
C_{out} : Nombre de cas MAS non couvert hors programme
R_{in} : Nombre en voie de guérison dans le programme
R_{out} : Nombre en voie de guérison hors du programme

$$R_{out} \approx \frac{1}{k} \times \left(R_{in} \times \frac{C_{in} + C_{out} + 1}{C_{in} + 1} - R_{in}\right)$$

Ou le facteur de correction «K» est toujours 3. Ceci à cause de la relation entre la durée moyenne d'un épisode MAS guéri dans le programme (2,5 mois) et la durée moyenne d'une guérison spontanée (7,5 mois).

- **Calcul de l'intervalle de confiance à 95%**

L'intervalle de confiance de 95% pour chacune des estimations de couverture a été calculé à l'aide de la formule suivante:

$$95\%\ CI = Coverage \pm 1.96 \times \sqrt{\sum \frac{\frac{c}{n} \times (1 - \frac{c}{n})}{n}}$$

[6] Pour plus d'information: Myatt, M et al, (2015) *A single coverage estimator for use in SQUEAC, SLEAC, and other CMAM coverage assessments*, p.81 Field Exchange 49.

CI = intervalle de confiance
c = numérateur
n = dénominateur

- **Données sur les barrières et boosters**

Le questionnaire cas couvert et non couvert a été administré à tous les enfants à l'exception des enfants bien portant pour la malnutrition aigüe. Ces questionnaires ont permis de déterminer les raisons principales de non-fréquentation (pour les cas non couverts) et les principales raisons de participation au programme (pour les cas couverts).

Le questionnaire pour **enfants non couverts** a pour but de permettre à l'enquêteur de déterminer les raisons principales de non-participation au programme. Le questionnaire suivait la logique suivante:
La première question a été posée aux accompagnants: en relation avec le dépistage et la périodicité. Par la suite la question suivantes a été posée : pensez-vous que votre enfant est malade? Si la réponse à cette question était « Non», alors l'intervieweur a mis fin au questionnaire. C'est parce que si l'accompagnant n'était pas conscient que leur enfant était malade, puis posant davantage de questions sur les symptômes et la maladie de l'enfant n'apportait pas une valeur ajoutée. Si l'accompagnant savait que leur enfant était malade, l'intervieweur a ensuite posé la question suivante:

- Questions: « De quels symptômes souffre votre enfant?»; «Quelle maladie a causé ces symptômes?"; "Comment avez-vous essayé de traiter cette maladie ou comment allez-vous la traiter?" Et; "Qui a pris une décision concernant le choix du traitement? ". Pour chacune de ces questions, les accompagnants pourraient fournir des réponses multiples.
- On a ensuite demandé aux accompagnants: Savez-vous qu'il existe un service dans l'établissement de santé dédié au traitement de la malnutrition? S'ils ont répondu non à cette question, l'intervieweur a mis fin au questionnaire.
- Si les accompagnants savaient que leur enfant était malade et qu'ils connaissaient l'existence d'un programme où ils pouvaient recevoir un traitement contre la malnutrition, on leur a ensuite demandé: pourquoi n'avez-vous pas amené votre enfant au centre de santé pour le traitement? Les intervieweurs devaient choisir une raison parmi une liste de raisons communément citées, ou ils pourraient sélectionner « Autre» et préciser la raison si la raison fournie n'était pas sur la liste.
- On a également demandé aux personnes qui connaissaient l'existence du programme si leur enfant avait été précédemment inscrits au programme et comment ils avaient été libérés (en tant que cas guéri, non-répondant).

L’objectif du questionnaire pour les **cas couverts** était d’identifier ce qui avait influencé l’accompagnant d’aller au poste de santé pour chercher un traitement pour leur enfant malnutri.

- La première question concernait l’accompagnant par rapport au dépistage et sa périodicité. Par la suite, on n’a tenu compte de la précédente inscription de leur enfant dans le programme de traitement. Si l’enfant avait rechuté ou avait fait défaut, l’interviewer demanderait de suivre des questions pour essayer de comprendre pourquoi.
- La deuxième question demandait si l’accompagnant avait d'autres enfants inscrits dans les programmes de nutrition
- La troisième question était la suivante: pourquoi avez-vous décidé d’inscrire votre enfant au programme de nutrition? Les intervieweurs devaient choisir une raison parmi une liste de raisons communément citées, ou ils pourraient sélectionnez « Autre» et spécifiez le motif.

3.4.Ressources Humaines de l'enquête

Cette enquête a été préparée par l'équipe technique de Nutrition d'ACF. Pour la phase de collecte des données sur le terrain, 6 équipes ont été formées. Chaque équipe était composée d'un superviseur et de deux enquêteurs, soit au total 6 superviseurs d'équipe, 12 enquêteurs et un superviseur général. Les équipes ont été supervisées en continue, avec une rotation ayant permis une vision d'ensemble des équipes.

3.5.Formation des enquêteurs

La formation des enquêteurs a été réalisée pendant 5 jours et avait regroupé tous les 12 enquêteurs, les 6 superviseurs et un superviseur général. Elle a comporté des séances en salle où ont été abordées les questions relatives à l'attitude générale et au comportement des enquêteurs, aux principes de remplissage du questionnaire, à la compréhension du questionnaire ainsi qu'à la traduction du questionnaire en langue local. Cette formation en salle a également comporté la présentation des outils de mesures anthropométriques et la démonstration de leur utilisation.
Avant le démarrage de l'enquête proprement dite, une pré-enquête a été effectuée dans des conditions réelles. Ceci a donné l'occasion aux enquêteurs et aux superviseurs de travailler dans le cadre de leurs équipes respectives, pour mettre en pratique toutes les étapes depuis l'introduction dans le village, l'échantillonnage, les interviews et la prise des mesures anthropométriques.
Cette pré enquête a permis de :

- tester et se familiariser aux questionnaires.
- adopter une méthode de travail sur le terrain
- adapter la logistique aux impératifs de déplacements fréquents.

3.6.Standardisation des mesures

Dans le cadre de la préparation des enquêteurs aux mesures anthropométriques, des enfants de 6 à 59 mois ont été identifiés pour participer à l'opération. Les superviseurs ont servi de référence pour les mesures de poids, taille et œdèmes.
Deux tests de standardisation des mesures anthropométriques ont été organisés, parallèlement, suivant les recommandations de la méthodologie SMART. Les agents travaillaient en binômes pour mesurer chacun deux fois (poids, taille, PB) 6 enfants de moins de cinq ans, à tour de rôle.
Les mesures ont été saisies et analysées sous le logiciel ENA, et les résultats obtenus ont permis d'évaluer la précision (écart observé entre deux mesures d'un même mesureur) et l'exactitude des mesures (écart observé entre la mesure de l'enquêteur et celle du formateur) pour chaque enquêteur, et de sélectionner les 12 meilleurs mesureurs pour

l'enquête. Quant à la taille, la précision a été jugée acceptable, cela était lié beaucoup plus à la fatigue et l'inattention, mais pas à une méconnaissance de la méthode

3.7.Traitement et analyse des données

- **Vérification et nettoyage des fiches sur le terrain**

Les fiches de collecte de données ont été quotidiennement vérifiées par chaque superviseur avant d'être reçues par le coordonnateur. A ce niveau, la base de données dans ODK est téléchargée afin d'analyser les données et si possible demander à chaque superviseur des informations supplémentaires. Nous avons identifié des données manquantes qui ont pu être complétées. Dans l'ensemble, tous ces problèmes ont été considérablement réduits après les 2 premiers jours de terrain.

- **Saisi et analyse statistique des données**

Toutes les données ont été analysées à partir des logiciels ENA-delta version juillet 2015 et Excel. Les indices nutritionnels ont été calculés en utilisant la population de référence OMS (2006).
La première saisie des questionnaires enfants (6-59 mois) s'est réalisée au cours de la phase de collecte des données, sur le terrain, par les superviseurs, sur le logiciel ENA-delta version janvier 2020 pour les données anthropométriques des enfants. Les données anthropométriques ont été saisies sur le lieu de collecte de la grappe du jour, avant de quitter.
La saisie dans la grappe du jour permettait de voir les erreurs/flags, de les corriger avant de quitter la grappe, et de donner un retour aux mesureurs sur la qualité de leurs mesures et de l'évaluation de l'âge. Chaque superviseur sauvegardait par ailleurs (et en plus de l'ordinateur), sur une clef USB, les fichiers de la saisie du jour.
La qualité de la collecte de données du jour était analysée par le superviseur accompagné de la coordination (rapport de plausibilité), afin de faire un retour le soir même aux équipes. Les superviseurs sauvegardaient, chaque jour, tous les fichiers (ENA) de leur équipe et transmettaient les données quotidiennement à la coordination. Une double saisie s'est effectuée au retour de la collecte des données.

4.Organisation sur le Terrain

Les superviseurs ont assisté à toutes les activités d'investigation et d'analyse des données de routine assurant un travail d'équipe pendant la complétude d'investigation.
Avec un manque de cartes assez détaillées dans toutes les aires de sante, les équipes ont été responsabilisées pour discuter avec les personnes clés/informées pour clarifier les axes de travail pour visiter les villages échantillonnés. Ainsi la planification détaillée des équipes était sous la responsabilité des superviseurs.

Un manuel de terrain a été préparé et distribué aux superviseurs et enquêteurs pour clarifier les procédures sur le terrain. Cela a permis dans une certaine mesure, de s'assurer que la méthodologie a été suivie d'une manière uniforme malgré la distance du superviseur et/ou le manque de réseaux téléphonique. Toutes autres questions et clarifications ont été discutées avec l'équipe d'enquête et les superviseurs tout au long de l'enquête pour diriger la collecte de données de qualité.
Pendant l'enquête, les activités ont été supervisées par l'équipe d'enquête avec les superviseurs en s'assurant sur le terrain que chaque équipe ait été visitée lors de la collecte de données qualitatives et pendant les enquêtes d'étape 2 et 3. Un formulaire de supervision a été développé pour assister les superviseurs dans l'identification des points à améliorer.

5.Limites de l'enquête

- **Imprécision dans l'âge des enfants :**

La majorité des enfants n'ont pas de document officiel précisant leur date de naissance. Les mamans ou membres des familles n'ont qu'une connaissance très approximative de l'âge des enfants. De ce fait, malgré les efforts des équipes et l'utilisation systématique du calendrier des évènements, la détermination de l'âge des enfants exigeait des profondes investigations.

- **Limite de l'évaluation**

Les données anthropométriques sont les seules informations quantitatives donc non subjectives pour apprécier la situation nutritionnelle des populations. Pour les données qualitatives ou quantitatives obtenues par interview sur l'état de santé, leur utilisation et interprétation ne peuvent se faire qu'à titre indicatif.

- **Imprécision dans la base des données démographiques** :

Dans certains villages identifiés, il arrivait parfois que les données démographiques mises à disposition par la région médicale soient inférieures à la réalité.

6.Problèmes rencontrés

La réussite de l'enquête est due certainement à la double participation des enquêteurs qui ont perçu l'enjeu de cette étude et des populations qui ont bien compris ses objectifs. Les problèmes rencontrés mais sans incidence sur les résultats sont:

- L'accessibilité géographique difficile pour certains villages.

- L'insécurité dans la région

7.Considérations éthiques

L'évaluation a été réalisée en respectant les principes éthiques suivants :

- Respect de l'anonymat et de la confidentialité ;
- Principe de non jugement ;
- Libre expression des personnes ;
- Fidélité des témoignages et opinions exprimés.

Les enfants de moins de 5 ans éligibles à une prise en charge de la malnutrition aigüe ont été systématiquement orientés et référés vers les centres de prise en charge les plus proches en cas de dépistage positif de la malnutrition aigüe modérée ou sévère.

Les autorités de chaque district ont été contactées et dûment informées de la réalisation de l'évaluation dans leur zone. Les équipes de terrain ont eu aussi la responsabilité d'informer les responsables de chaque localité avant leur arrivée et solliciter leur appui, selon le besoin.

IV. RESULTAT

1.Description de l'échantillon

Tableau 7*:* Synthèse des données collectées, Gao, mars 2020

Paramètre	Contrôle	Bras 1	Bras 2	Total
**N° Villages visités**	20	18	25	63
**N° Enfants dépistés**	1387	1833	1609	4829
**Cas MAM**	168	123	139	430
**Cas MAS**	72	49	55	176
**Total MAM+MAS**	240	172	194	606

Le tableau 7 montre que dans les trois groupes, les équipes ont évalué 4829 enfants dont 606 atteints de malnutrition aiguë (MAM et MAS) dans 63 villages en utilisant les étapes décrites dans le formulaire d'enregistrement des enfants malnutris aigue pendant un mois de collecte de données. Cela représente environ 14,7% de la population totale d'enfants de moins de cinq ans (32877) dans les 3 bras (contrôle, bras 1 et bras 2) étudiées dans les districts de Gao.

Le tableau 7 présente un résumé de la collecte des données. Dans les villages échantillonnés 240, 172, 194 cas de MAS et MAM ont été trouvés respectivement pour le bras control, bras 1 et bras 2, ce qui représente environ 39,6%,28,4% et 32% de tous les enfants MAS et MAM identifiés respectivement pour le bras contrôle, bras 1 et bras 2. Le bras contrôle à la taille d'échantillon(MAM+MAS) des cas identifiés la plus élevée.

Tableau 8*:* Comparaison taille de l'échantillon attendue et atteinte dans les zones d'intervention et contrôle, mars 2020

Taille de l'échantillon	Contrôle			Bras 1			Bras [illegible]	
	**Taille échantillon attendue**	**Taille échantillon atteinte**	**%**	**Taille échantillon attendue**	**Taille échantillon atteinte**	**%**	**Taille échantillon attendue**	**Ta[illegible] écha[illegible] att[illegible]**
**MAS**	61	**72**	118%	47	**49**	104%	52	[illegible]
**MAM**	89	**168**	189%	84	**123**	146%	86	[illegible]

Pour pouvoir estimer la couverture de la MAM et MAS, il était nécessaire que les équipes d'enquête identifient les tailles d'échantillon cibles de chacune d'elles. Les tailles d'échantillon ont été calculées en fonction de la population attendue d'enfants de 6 à 59 mois concernée dans chacun des bras de l'étude.

Le tableau 8 montre les cas constatés à la fin de l'enquête dans les 63 villages sélectionnés. Les tailles d'échantillon ont été atteintes ou dépassées pour la MAS et MAM dans les trois

bras. Lorsque la taille des échantillons était atteinte ou dépassée, il était possible d'estimer la couverture du traitement de la malnutrition aigüe dans les différents bras

2.Estimation de la couverture

- **Bras contrôle**

Tableau 9: Nombre de cas couvert et non couvert pour le bras contrôle selon les critères d'admission, Gao mars 2020

Type de malnutrition selon la couverture	OEDEME	PB	PB et Œdème	PT	PT et PB	Couverture uniquement avec PB	Couverture uniquement avec P/T+PB+oedeme
MAM couvert		3			4	7	7
MAM en voie de guérison		4		18		4	22
MAM non couvert		56		40	43	99	139
MAS couvert	3	3	1		2	9	9
MAS en voie de guérison		4		1	4	8	9
MAS non couvert		14		24	16	30	54
**Total**	3	84	1	83	69	157	240

Le tableau 9 montre que pour les enfants couverts et en voie de guérison en tenant compte du critère (PB + PT + œdème) pour la MAS est 9 pour chacun. Pour la MAM couvert et en voie de guérison, on a 7 et 22 respectivement.
En outre, si un programme fonctionne correctement, dans les cas non couverts pour la MAS on a 55,6% qui devraient être détectés exclusivement par le PB/œdème et 44,4 % exclusivement par le PT. Pour la MAM non couvert on 71,2% qui devraient être détectés par PB et 28,8% exclusivement par le PT. Ces résultats montreraient une non fonctionnalité du système de surveillance nutritionnelle.

- **Bras 1**

Tableau 10: Nombre de cas couvert et non couvert pour le bras 1 selon les critères d'admission, Gao mars 2020

Type de malnutrition selon la couverture	OEDEME	PB	PT	PT et PB	Couverture uniquement avec PB	Couverture uniquement avec P/T+PB+oedeme
MAM couvert		6	2	4	10	12
MAM en voie de guérison		7		1	8	8
MAM non couvert		31	45	27	58	103
MAS en voie de guérison		2	1	3	5	6

MAS couvert		3	4	1	4	8
MAS non couvert	1	13	9	12	25	35
**Total**	1	62	61	48	110	172

Le tableau 10 montre que pour les enfants couverts et en voie de guérison en tenant compte du critère (PB + PT + œdème) pour la MAS est 6 et 8 respectivement. Pour la MAM couvert et en voie de guérison, on a 12 et 8 respectivement.
En outre, si un programme fonctionne correctement dans les cas non couverts pour la MAS on a 74,3% qui devraient être détectés par le PB/œdèmes et 25,7% exclusivement par le PT. Pour la MAM non couvert on a 56,3% qui devraient être détectés par PB, et 43,7% exclusivement par le PT. Ces résultats montreraient une non fonctionnalité du système de surveillance nutritionnelle.

- **Bras 2**

Tableau 11: Nombre de cas couvert et non couvert pour le bras 2 selon les critères d'admission, Gao mars 2020

**Type de malnutrition selon la couverture**	**Œdème**	**PB**	**PT**	**PT et PB**	**Couverture uniquement avec PB**	**Couverture uniquement avec P/T+PB+oedeme**
MAM couvert		2	6	6	8	14
MAM envoie de guérison		4			4	4
MAM non couvert		39	44	38	77	121
MAS en voie de guérison			1	4	4	5
MAS couvert				1	1	1
MAS non couvert	1	11	33	4	16	49
**Total**	1	56	84	53	110	194

Le tableau 11 montre que pour les enfants couverts et en voie de guérison en tenant compte du critère (PB + PT + œdème) pour la MAS est 1 et 5 respectivement. Pour la MAM couvert et en voie de guérison, on a 14 et 4 respectivement.
En outre, si un programme fonctionne correctement, dans les cas non couverts pour la MAS on a 32,7% qui devraient être détectés par le PB/œdèmes et 63,6% exclusivement par le PT. Pour la MAM non couvert on a 36,4% qui devraient être par PB, et 36,4% exclusivement par le PT. Ces résultats montreraient une non fonctionnalité du système de surveillance nutritionnelle.

- **Couverture de chaque bras**

Tableau 12: Couverture unique de la MAS et MAM pour chaque bras du projet, Gao, mars 2020

		Cas collectés				Dénominateur	Numérateur	Estimation de la couverture	Intervalle de confiance (IC) 95%				
						n	C	C/n	1-c/n	(c/n*1-c/n)/n	Intervalle		
		Cin (Nbre de Cas couvert)	Cout (Nbre de Cas non-couvert)	Rin (Nbre de Cas EVG)	Rout	Cin + Cout + Rin + Rout	Cin + Rin	Numérateur / dénominateur				Borne inferieure	Borne supérieure
Contrôle	MAS	9	54	9	16	88	18	**20,5%**	0,80	0,00184894	8,43%	**12,0%**	**28,9%**
	MAM	7	139	22	127	295	29	**9,8%**	0,90	0,00030048	3,40%	**6,4%**	**13,2%**
Bras 1	MAS	8	35	6	7	56	14	**25,0%**	0,75	0,00334821	11,34%	**13,7%**	**36,3%**
	MAM	12	103	8	21	144	20	**13,9%**	0,86	0,00083055	5,65%	**8,2%**	**19,5%**
Bras 2	MAS	1	49	5	40	95	6	**6,3%**	0,94	0,00062283	4,89%	**1,4%**	**11,2%**
	MAM	14	121	4	10	149	18	**12,1%**	0,88	0,00071283	5,23%	**6,8%**	**17,3%**

Les résultats de l'enquête dans le tableau 12 indiquent que la couverture du traitement de la MAS et MAM sont très faibles et en dessous des normes sphères en zone rurale qui est de 50%. Cette couverture est plus faible pour les MAM (9,8% pour le bras contrôle, 13,9% pour le bras 1). Par ailleurs, la couverture de la MAM dans le bras 2 est plus élevée que celle de la MAS. Les couvertures des MAS varient d'un bras à l'autre :20,5% pour le bras contrôle, 25,0% pour le bras 1 et 6,3% pour le bras 2.

- **Couverture par centre de santé communautaire**

Les résultats sont également résumés par CSCOM dans le tableau 13, 14 et 15 dans lesquelles les estimations de la couverture servent à indiquer l'estimation approximative de la couverture dans chaque CSCOM et ne doivent pas être utilisées comme

des estimations de la couverture individuelle dans la mesure qu'il ne s'agit pas d'une couverture unique et la taille de l'échantillon n'est pas appropriée. Ces résultats par CSCOM sont à utiliser à titre indicatif.

Tableau 13: Couverture de période pour le bras contrôle, Gao mars 2020

CSCOM	MAM_C	MAM_EVG	MAM_NC	MAS_C	MAS_EVG	MAS_NC	GAM couvert	GAM total	couverture période[7]
**Aljanabandia**	4	5	45	2	3	24	14	83	16,9%
**Djoulabougou Saneye**		5	20	1	0	9	6	35	17,1%
**Gadeye**		7	37	5	3	14	15	66	22,7%
**Kochakareye**	3	1	22	1	3	2	8	32	25,0%
**Tin Aouker**		4	15	0	0	5	4	24	16,7%

Les résultats du tableau 13 indiquent que dans le bras contrôle, toutes les couvertures seraient très faibles par CSCOM et en dessous de 50% et elle serait plus faible dans le CSCOM de Tin Aouker.

Tableau 14: Couverture de période pour le bras 1, Gao mars 2020

CSCOM	MAM_C	MAM_EVG	MAM_NC	MAS_C	MAS_EVG	MAS_NC	GAM couvert	GAM total	couverture période
**Forgho**	7	5	15	2	3	6	17	38	44,7%
**Lobou**	1	1	36	3	1	16	6	58	10,3%
**Zinda**	4	2	52	1	4	13	11	76	14,5%

Les résultats du tableau 14 indiquent que le bras 1, le CSCOM Forgho aurait la couverture la plus élevée (44,7%). Tous les deux autres CSCOM auraient des couvertures très faibles.

Tableau 15: Couverture de période pour le bras 2, Gao mars 2020

[7] Lors de l'estimation de la couverture de traitement MAG par poste de santé, il n'est pas approprié de calculer R_{out} et d'estimer la couverture à l'aide de «l'estimateur de couverture unique». Par conséquent, l'estimateur de couverture par période est utilisé ($GAM_{couvert}$ = Cin + Rin; GAM_{total} = Cin + Cout + Rin).

CSCOM	MAM_C	MAM_EVG	MAM_NC	MAS_C	MAS_EVG	MAS_NC	GAM couvert	GAM total	couverture période
**Bagnadji**	14	3	41	4	1	27	22	90	24,4%
**Magnadoue**	0	1	80	1	0	22	2	104	1,9%
**Wabaria**	0	0	0	0	0	0	0	0	0

Les résultats du tableau 15 indiquent que dans le bras 2, aucun CSCOM n'atteint pas la couverture de 50%.

3.Raison de la non couverture du programme

Quand un enfant était couvert ou non couvert, un questionnaire précis, selon sa couverture ou pas, lui était administré. Au total, 193,138,170 enfants se sont avérés être des cas non couverts respectivement pour le bras contrôle, bras 1 et bras 2.
Pour les enfants couverts, nous avons enregistré 47,34 et 24 cas respectivement pour le bras contrôle, bras 1 et bras 2. A partir des différentes réponses, il est possible de déterminer la principale raison de la non couverture ou couverture pour chaque cas.

La raison la plus souvent évoquée dans le bras contrôle, bras 1 et bras 2 (figure 2,3 et 4) pour les non couverts est le problème de rupture de stock dans les CSCOM qui apparait dans les trois bras. Le bras 2 montre que la rupture de stock est plus accentuée chez les MAS non couverts que chez les MAM non couverts. Dans les trois bras pour les enfants non couverts, on observe en moyenne 54,3% des cas MAM non couverts (bras contrôle=56,4%, bras 1=82,1%, bras 2=59,2%) et 50,3 %des cas MAS non couverts (bras contrôle=51,9%, bras 1=66,7%, bras 2=33,3%) ont évoqué le problème de rupture de stock.
Pour le Manque des moyens financiers pour le déplacement, il est plus accentué dans le bras 2 par rapport aux autres bras. La majorité des accompagnants qui ont cité le problème de manque des moyens financiers pour le déplacement est en moyenne de 8,1% pour les cas MAM non couverts (bras contrôle=5,5%, bras 1=7,1%, bras 2=11,8%) et 33,0% pour les cas MAS non couverts(bras contrôle=32,7%, bras 1=23,8%, bras 2=42,4%).

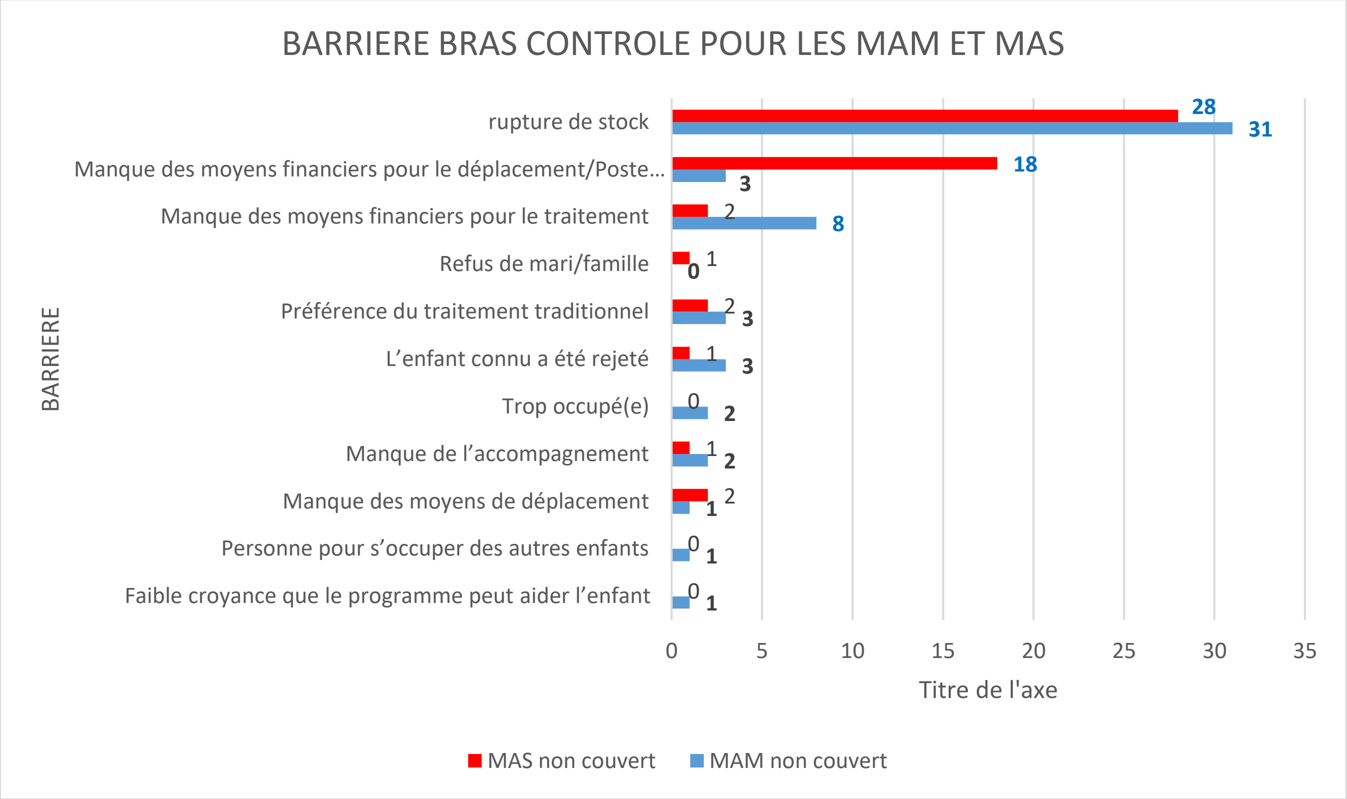

Figure 2 : Raison de non accès aux soins de prise en charge de la MAM et MAS bras contrôle, mars 2020

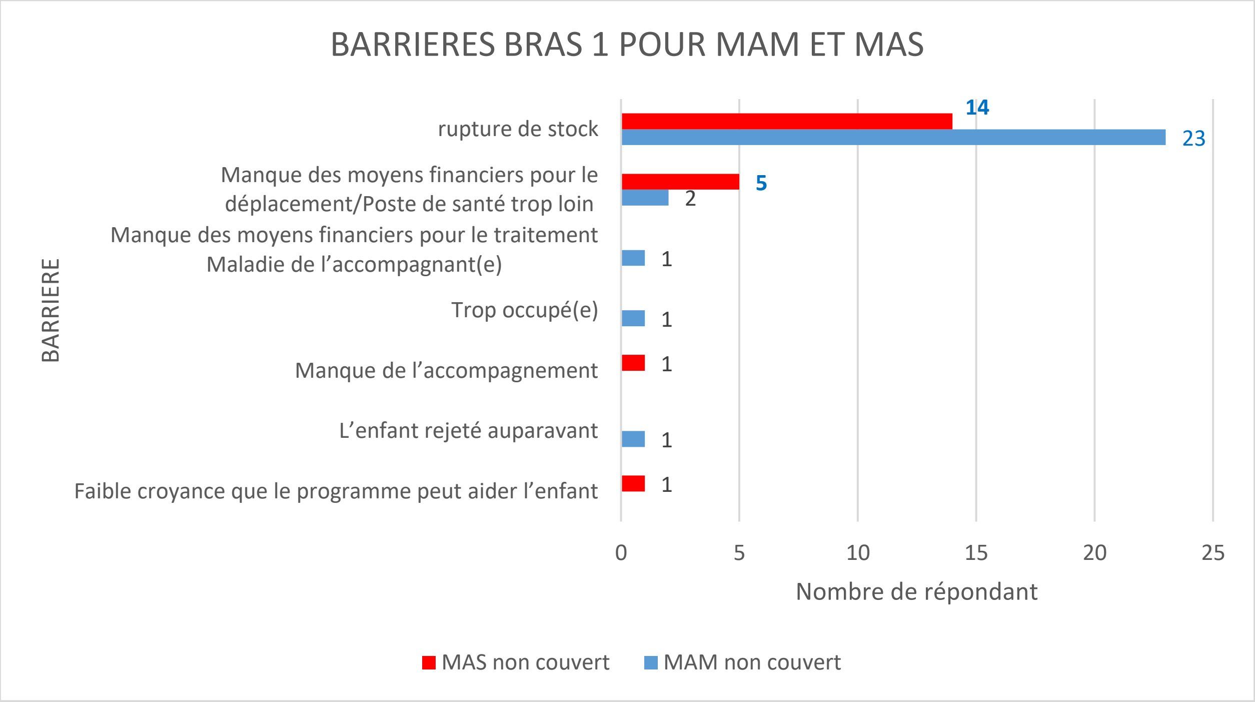

Figure 3 : Raison de non accès aux soins de prise en charge de la MAM et MAS bras1, mars 2020

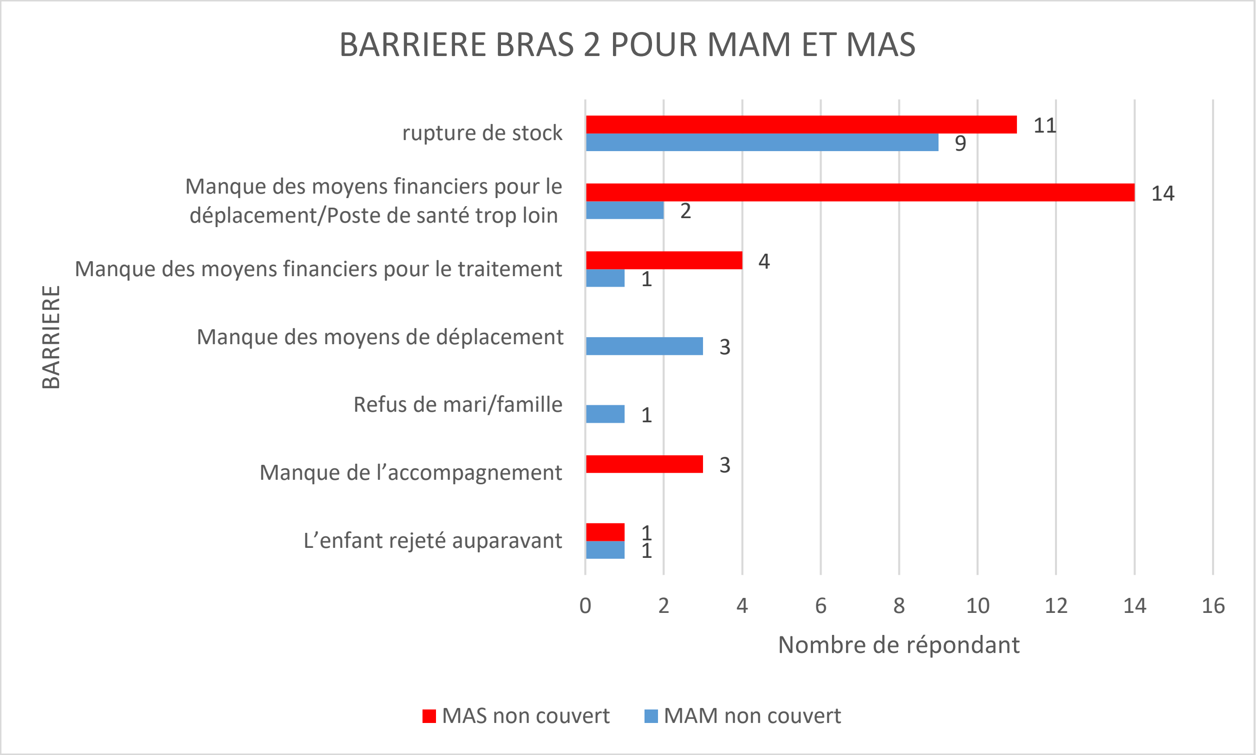

Figure 4 Raison de non accès aux soins de prise en charge de la MAM et MAS bras 2, mars 2020

➢ **Dépistage communautaire**

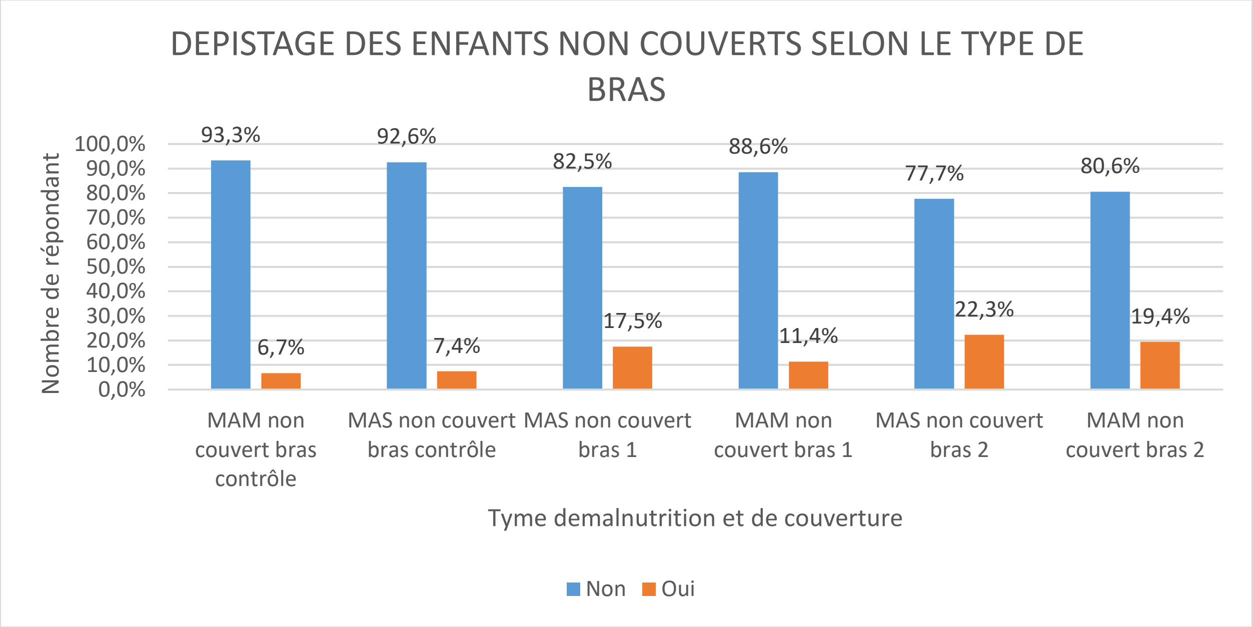

Figure 5 Raison de non accès aux soins de prise en charge de la MAM et MAS bras 2, mars 2020

La figure 5 indique que les relais communautaires n'effectuent pas régulièrement les dépistages porte-à-porte au niveau des enfants non couverts. Dans l'ensemble pour la MAS et MAM confondues, 85,9 % (bras contrôle=93,0%, bras 1=87,6%, bras 2=85,5%) des enfants n'ont pas été dépistés quelque soit le bras le mois précédent l'enquête. Par contre 14,1% (bras contrôle=7,0%, bras 1=12,4%, bras 2=14,5%) d'enfant ont été dépisté le mois précédent. Le dépistage s'effectuerait en général pendant les campagnes de vaccination ou par des dépistages de masse organisés par la croix rouge malienne.

➢ Lieu de traitement

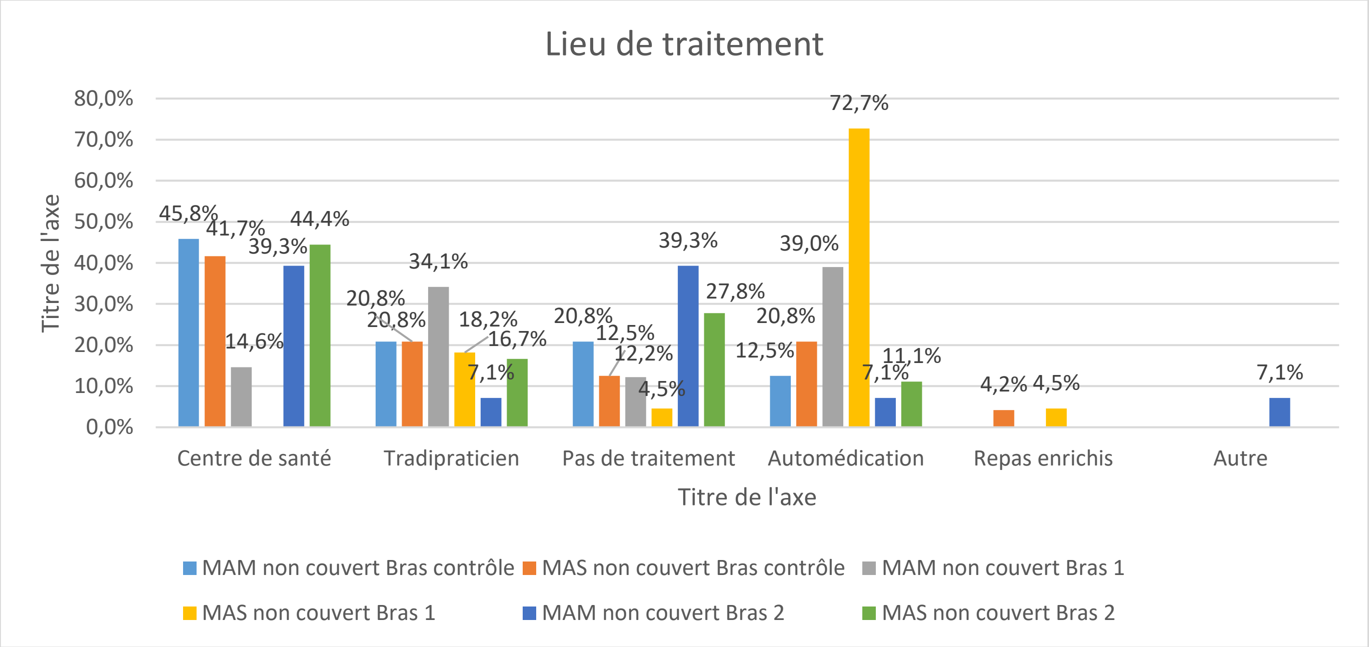

Figure 6 Lieu de traitement dans le bras contrôle, bras 1 et bras 2, mars 2020

Dans le bras contrôle, 1 et 2 (figure 6) en moyenne 31%(bras contrôle=43,8%, bras 1=7,3%, bras 2=41,9%) des accompagnants des MAM et MAS non couverts partent immédiatement au CSCOM quand l'enfant est malade. Par contre, 19,6%(bras contrôle=20,8%, bras 1=26,2%, bras 2=11,9%) des accompagnants partent en première intention chez les tradipraticiens. Par ailleurs, 27,2% (bras contrôle=16,7%, bras 1=55,9%, bras 2=9,1%) des accompagnants font de l'automédication en première intention quand l'enfant est malade.

➢ Connaissance PCIMA

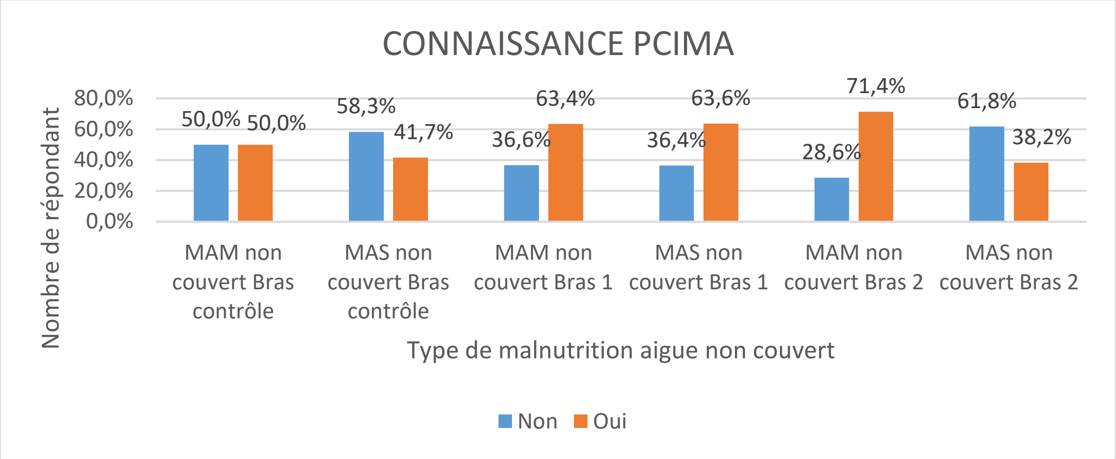

Figure 7 Connaissance de la PCIMA dans le bras contrôle, bras 1 et bras 2, mars 2020

La figure 7 montre que pour les MAM et MAS non couverts, 45,8%, 63,5%, et 54,8% connaissent la PCIMA respectivement pour le bras contrôle, bras 1 et bras 2. Ainsi en moyenne 54,7% des accompagnants connaissent la PCIMA.

En outre, 45,3% des accompagnants (bras contrôle=54,2%, bras 1=36,5%, bras 2=45,2%) ne connaissant pas la PCIMA. Cette non connaissance de la PCIMA est plus accentuée dans le bras contrôle et le bras 2 alors que la connaissance de la PCIMA est plus importante dans le bras 1.

- **Connaissance de la maladie**

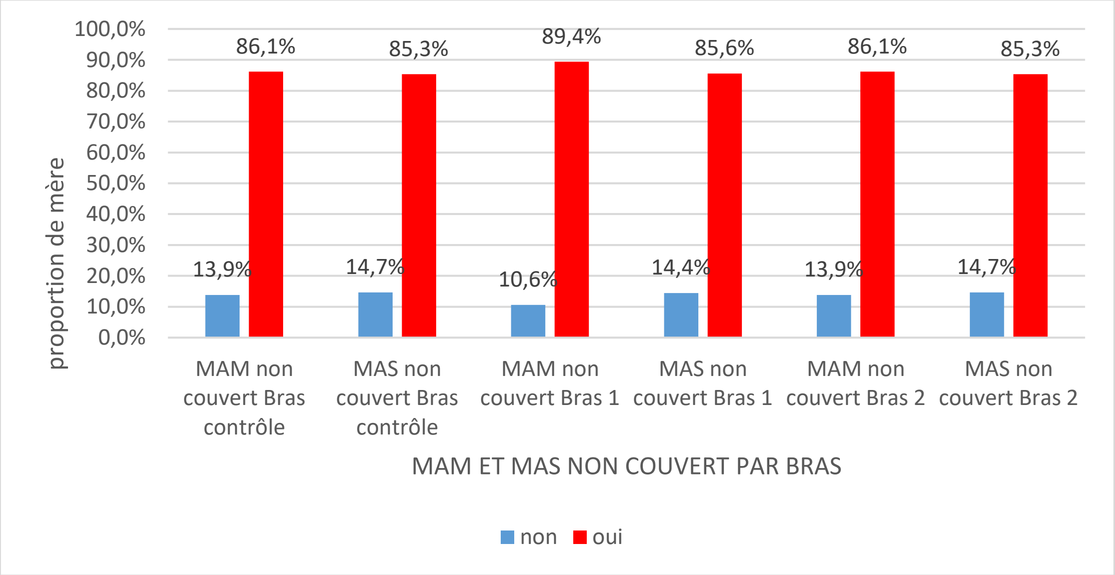

Figure 8 connaissance des maladies infantiles dans le bras contrôle, bras 1 et bras 2, mars 2020

La figure 8 montre qu'environ 85,7% des mères (bras contrôle=85,7%, bras 1=87,5%, bras 2=85,7%) connaissent les maladies infantiles et il n'y a aucune disparité entre les MAM et les MAS

➢ **Raison d'abandon du programme**

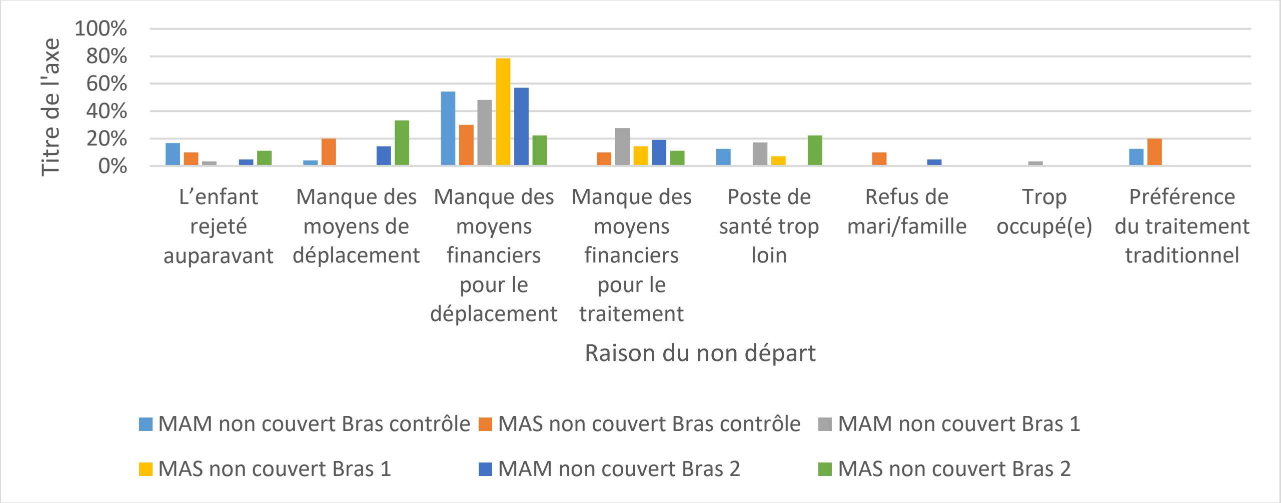

Figure 9 Raison d'abandon du programme dans le bras contrôle, bras 1 et bras 2, mars 2020

La figure 9 montre que la grande raison d'abandon du programme est en moyenne le manque de moyen financier pour le déplacement pour 46 % pour les 3 bras

➢ **Perception de la PCIMA**

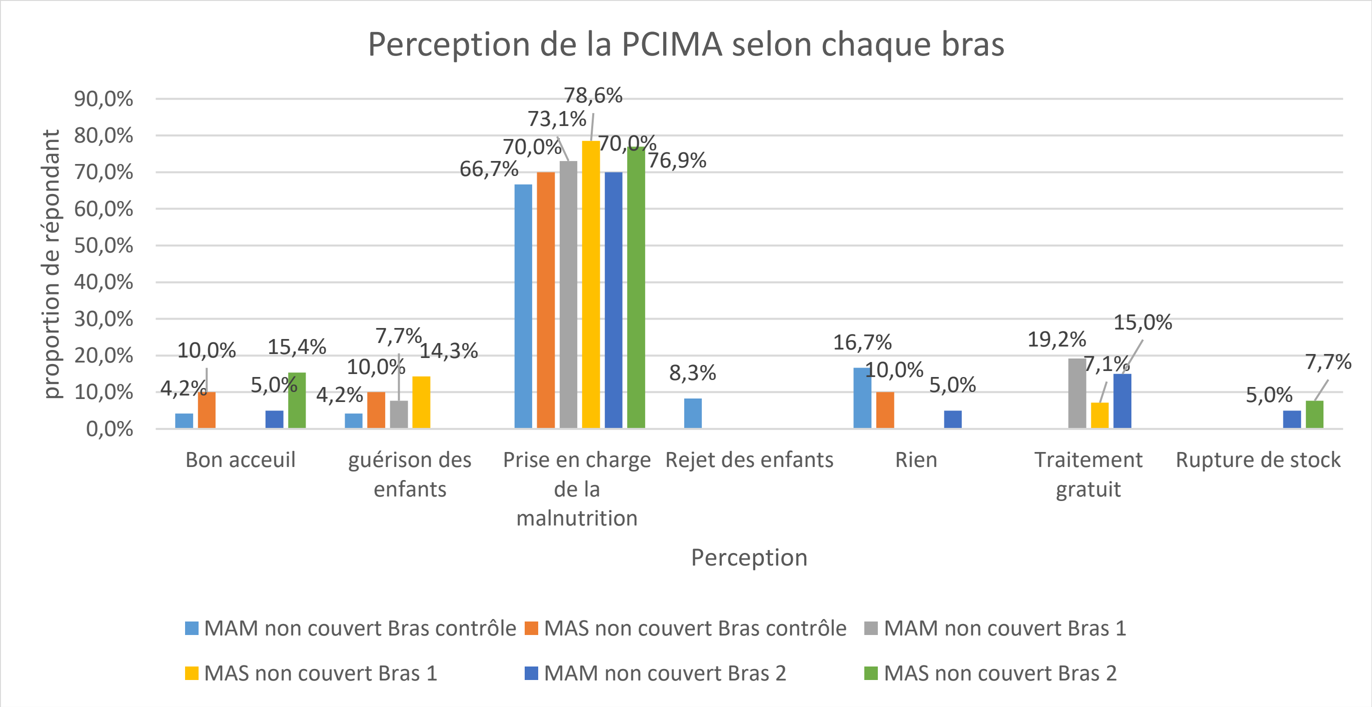

Figure 10 Perception de la PCIMA dans le bras contrôle, bras 1 et bras 2, mars 2020

La figure 10 montre qu'en moyenne 72,5% des accompagnants ont une bonne perception de la PCIMA comme un endroit où on traite les enfants malnutris correctement.

- **Prise de décision**

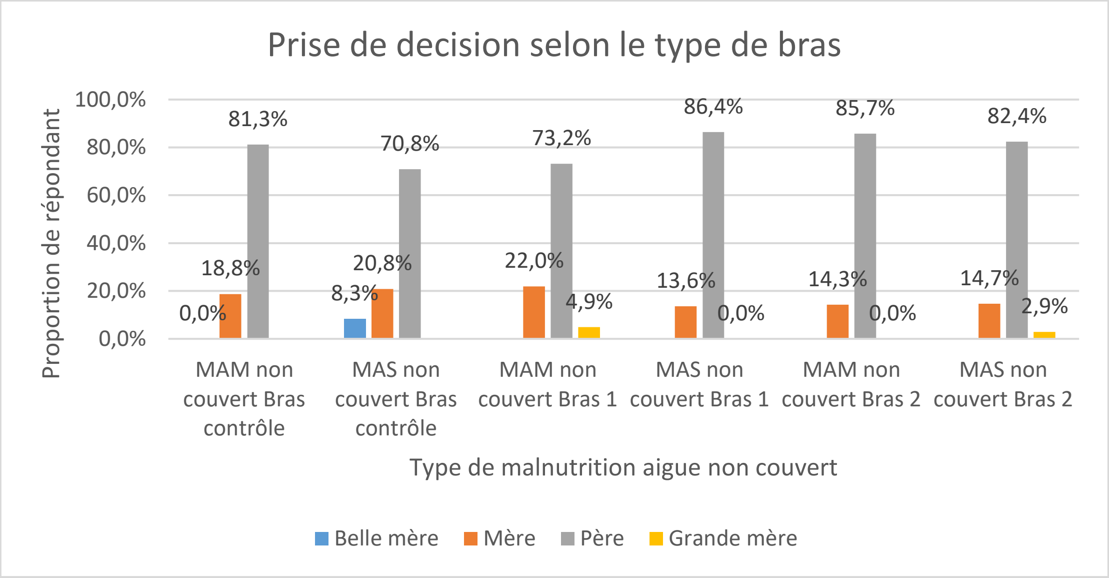

Figure 11 Prise de décision pour aller dans les structures de santé dans le bras contrôle, bras 1 et bras 2, mars 2020

La figure 11 montre qu'en moyenne 79,9% des pères (bras contrôle=76,0%, bras 1=79,8%, bras 2=84,0%) prennent la décision pour amener l'enfant au poste de santé quel que soit le bras. Néanmoins, cette proportion est plus élevée dans le bras 2. Par contre, 17,4% des mères ont leur mot à dire avant le départ de l'enfant au poste de santé.

4. Raison de la couverture du programme

➢ **Dépistage communautaire**

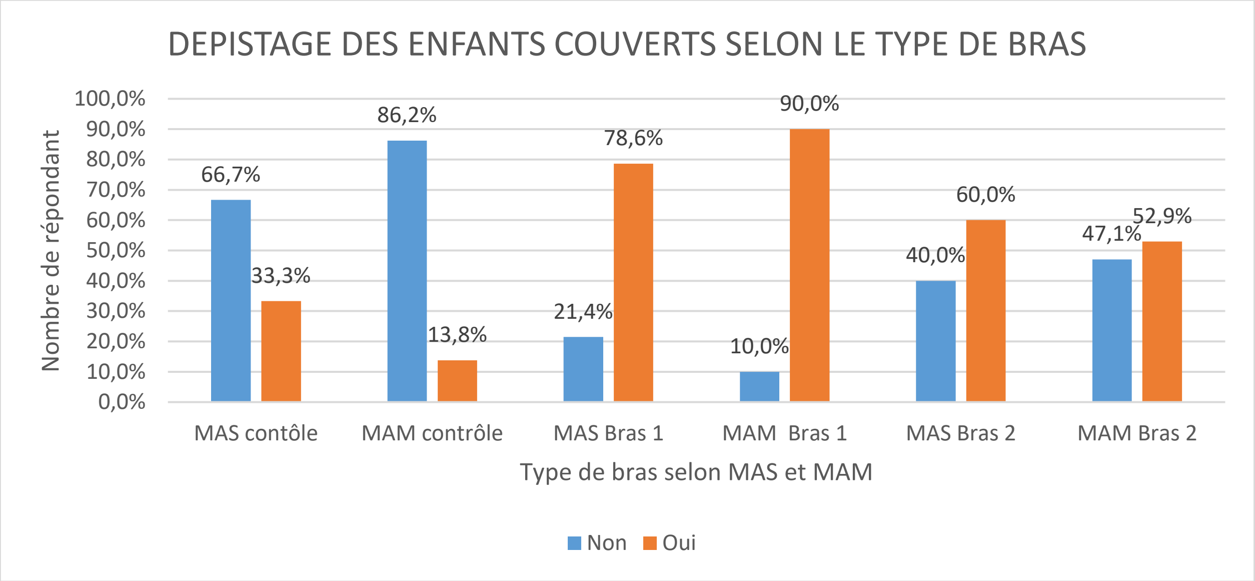

Figure 12 Dépistage des enfants MAM et MAS, bras contrôle, 1 et 2, mars 2020

La figure 12 montre qu'en moyenne 54,8% des enfants couverts ont été identifiés par dépistage contre 45,2% qui n'ont pas été dépistés. Les enfants non dépistés et couverts se seraient rendus volontairement au centre de santé. En analysant par bras, on constate que 84,3% ont été dépistés dans le bras 1 suivi du bras 2 (56,5%) et enfin 23,6% pour le bras contrôle.

➢ **Booster**

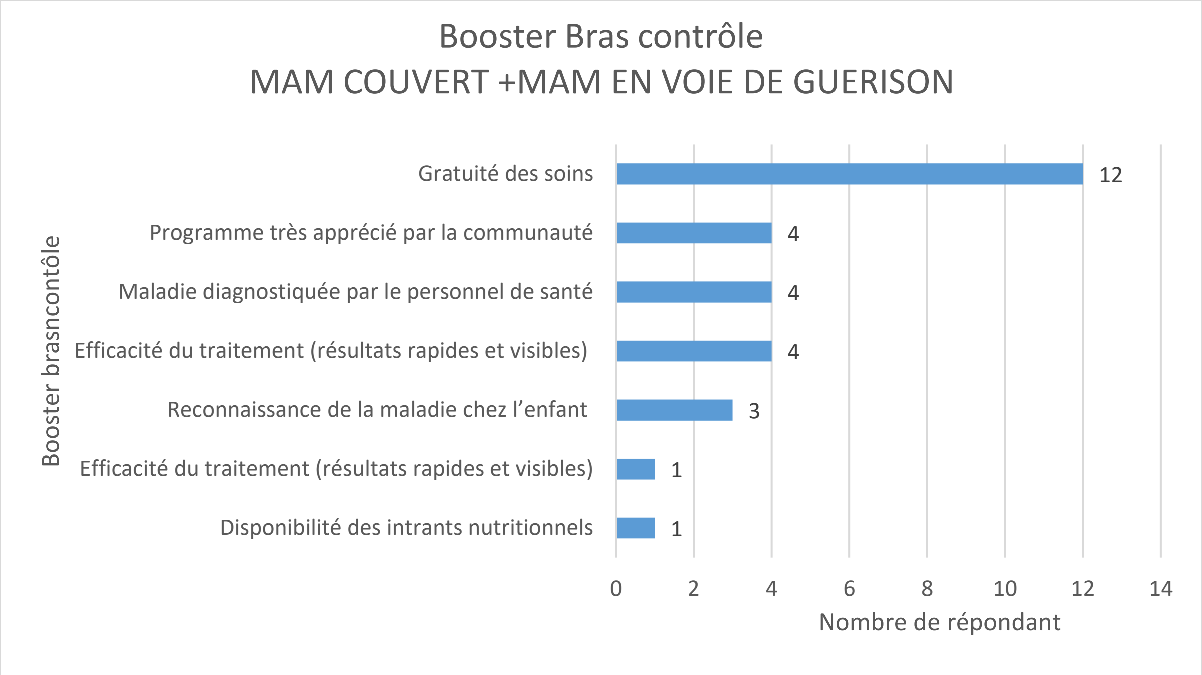
Booster Bras contrôle
MAM COUVERT +MAM EN VOIE DE GUERISON
Booster brasncontôle
Gratuité des soins
Programme très apprécié par la communauté
Maladie diagnostiquée par le personnel de santé
Efficacité du traitement (résultats rapides et visibles)
Reconnaissance de la maladie chez l'enfant
Efficacité du traitement (résultats rapides et visibles)
Disponibilité des intrants nutritionnels
12
4
4
4
3
1
1
0
2
4
6
8
10
12
14
Nombre de répondant

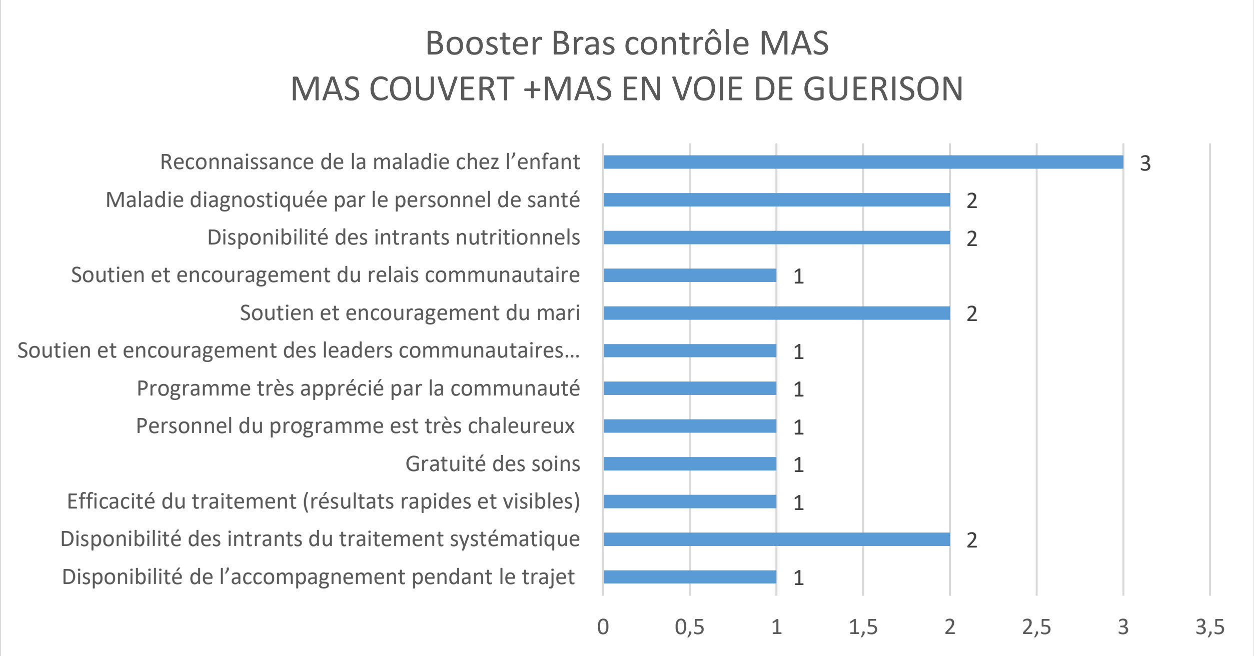
Booster Bras contrôle MAS
MAS COUVERT +MAS EN VOIE DE GUERISON
Reconnaissance de la maladie chez l'enfant
Maladie diagnostiquée par le personnel de santé
Disponibilité des intrants nutritionnels
Soutien et encouragement du relais communautaire
Soutien et encouragement du mari
Soutien et encouragement des leaders communautaires…
Programme très apprécié par la communauté
Personnel du programme est très chaleureux
Gratuité des soins
Efficacité du traitement (résultats rapides et visibles)
Disponibilité des intrants du traitement systématique
Disponibilité de l'accompagnement pendant le trajet
3
2
2
1
2
1
1
1
1
1
2
1
0
0,5
1
1,5
2
2,5
3
3,5

Figure 13 Booster dans le bras contrôle, mars 2020

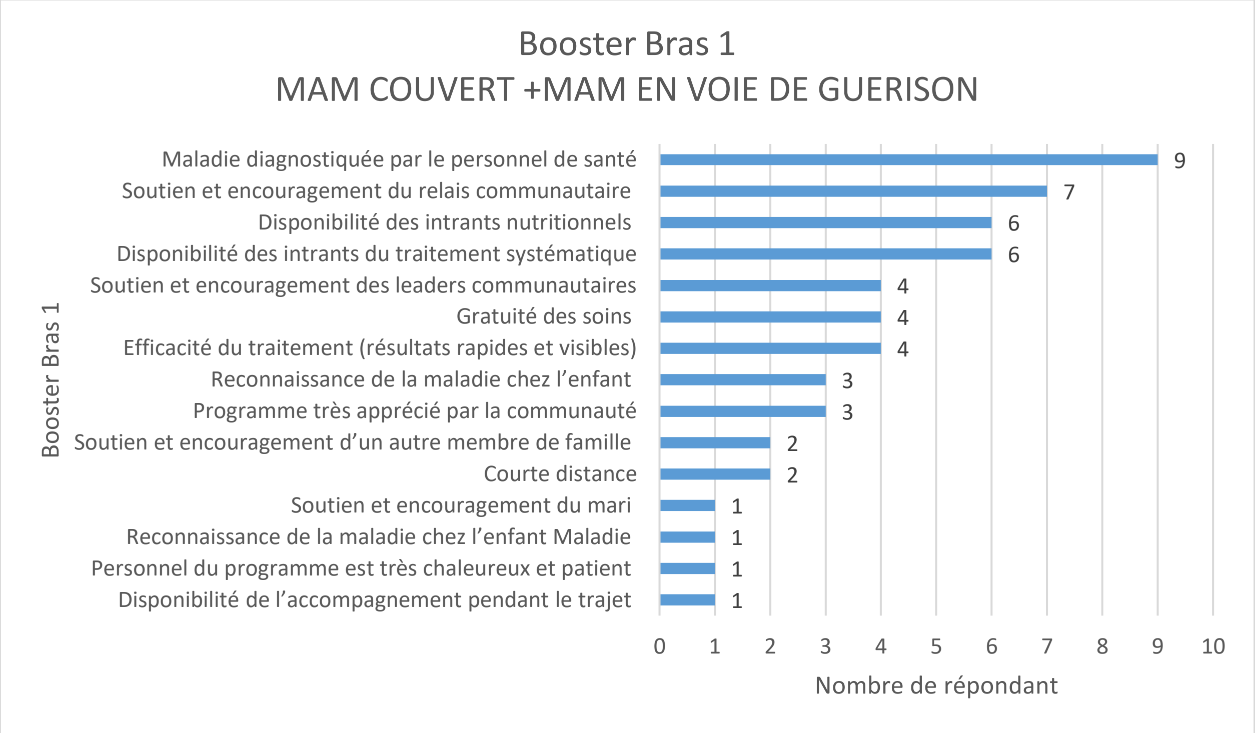

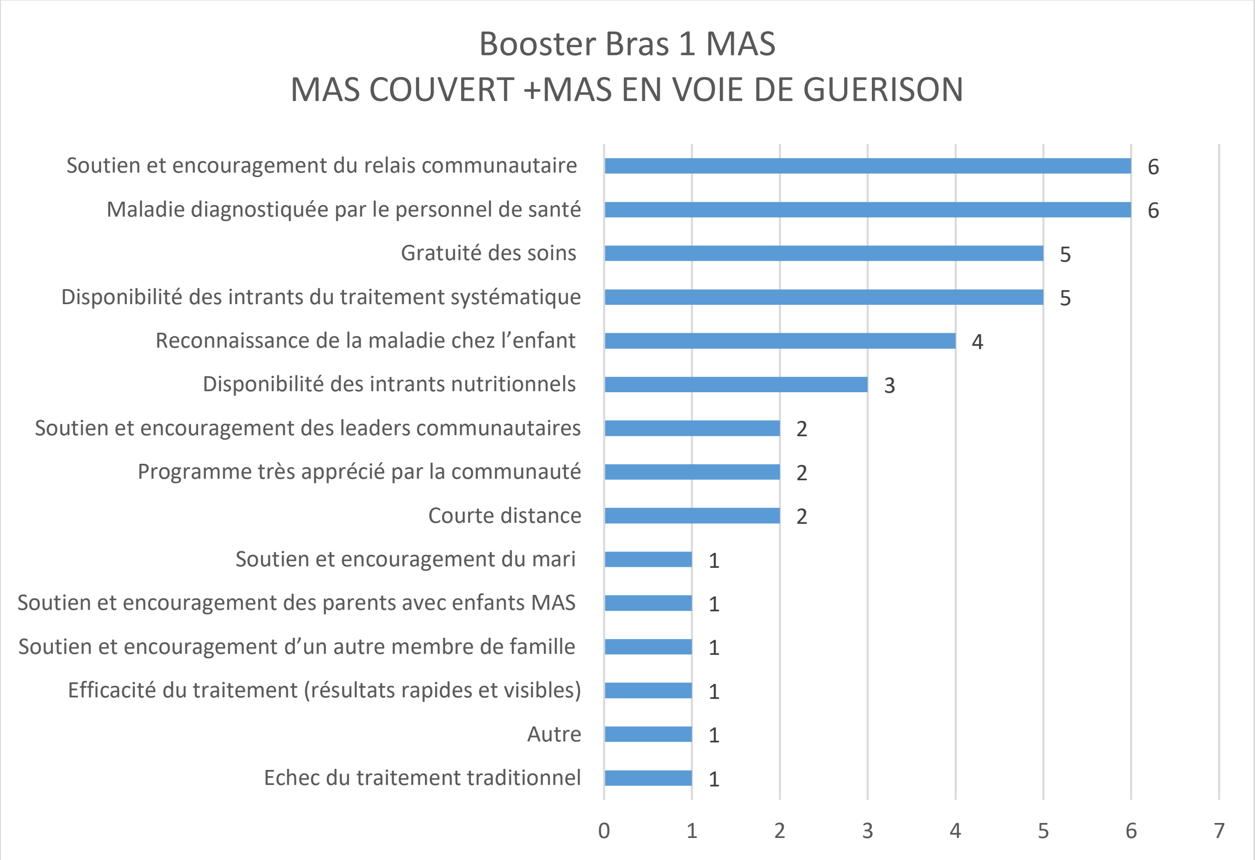

Figure 14 Booster dans le bras 1 , mars 2020

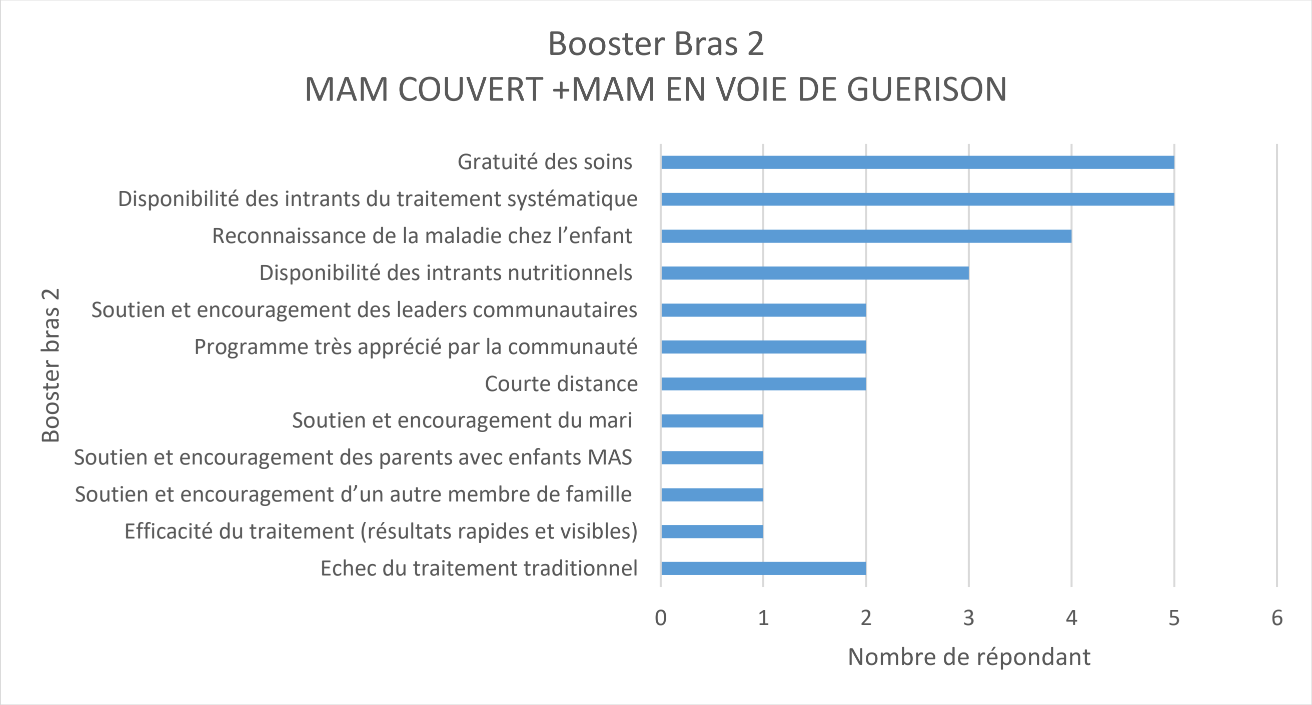

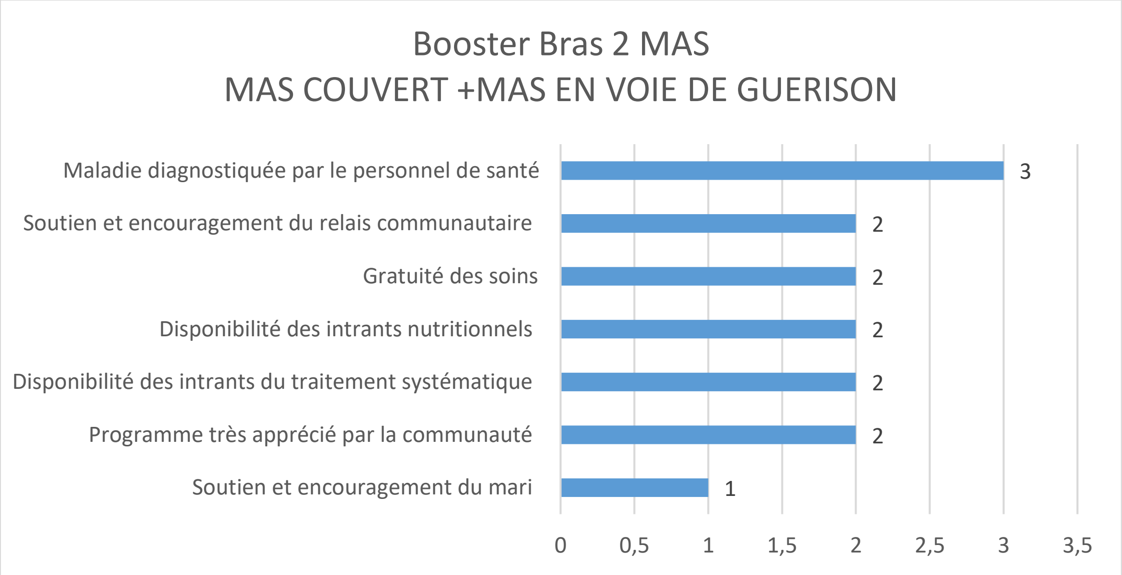

Figure 15 Booster dans le bras 2, Gao mars 2020

Les figures 13,14 et 15 montrent que les principaux booster dans les trois bras sont : le diagnostic de la maladie par le personnel de santé, suivi de la reconnaissance de la maladie de l'enfant par l'accompagnant et enfin le soutien et l'encouragement des relais communautaires pour que les mères puissent suivre le programme PCIMA jusqu'à la guérison de leur enfant.

4.Analyse qualitative

➢ Description des boosters

Code	Méthode
1	**FG=** Discussion de groupe semi-directive
2	**ESS=** Entretien semi-directif
3	**Quantitatif**

Code	Cible
**F**	Femmes de la communauté
**H**	Hommes de la communauté
**A$^{MAS\ F}$**	Accompagnante MAS (femme)
**GT**	Guérisseur traditionnel/ Accoucheuse traditionnelle
**RC**	Relais communautaire
**IM**	IMAM
**CV**	Les leaders communautaires
**IT**	Infirmier titulaire

Tableau 16: Description des boosters, bras Contrôle: Gao mars 2020

BOOSTERS	Arguments	Source	Méthode
Connaissance des Maladies infantiles	Les maladies infantiles sont la diarrhée, le paludisme, l'IRA, rougeole, fièvre jaune pendant la saison sèche et froide	IT,IT,F,LE,F,F,A,RC,RC,RC,LE,RC,H,H,H	2,2,2,2,1,1,2,2,2,2,2,2,1,1,1,3
Connaissance de la malnutrition	Le terme local de la malnutrition est "Zanka Yalafautey" qui se manifeste par l'amaigrissement, œdème, la diarrhée, vomissement, la déshydratation et est causé par la pauvreté et le sevrage précoce. La malnutrition est une maladie par carence. Un IMAM dit que la malnutrition est plus présente pendant la période de soudure	IT,IT,F,LE,F,F,A,A,A,RC,RC,RC,RC,H,H,H	2,2,2,2,1,1,2,2,2,2,2,2,2,1,1,1
Sensibilisation communautaire et dans les CSCOM	Les relais font la sensibilisation auprès des femmes dans la communauté sur la démonstration culinaire, la malnutrition, lavage des mains, ANJE, vaccination, CPN, la planification familiale auprès des mères. La sensibilisation se fait en général au cours des campagnes de vaccination ou lors des visites des patients dans les CSCOMS. Dans certaines localités, la sensibilisation est réalisée lors des campagnes de vaccination (Djoulabougou). Les hommes sont moins sensibilisés que les femmes	IT,IT,A,RC,RC,RC,RC,LE	2,2,1,2,2,2,2,2
Qualité du service PCIMA	les mères sont contentes de l'accueil, de la prise en charge des enfants et surtout de la gratuité des soins, Tous les outils sont disponibles pour la prise en charge de la malnutrition	IT,F,A,A,RC	2,1,2,2,2
Perception des services PCIMA	La PCIMA aide la communauté à avoir une bonne santé. La PCIMA est une grande aide pour notre communauté car il y a guérison rapide des enfants malades	IT,F,A,RC,LE	2,1,2,2,2,3
Formation	Les agents du CSCOM ont été formés par ACF, IntraHealth et MDM sur la PCIMA	IT,F,RC	2,1,2
connaissance des services PCIMA	Les services PCIMA sont connus à causes des RC qui font la vulgarisation dans les villages, La malnutrition se traite avec du plumpy nut. Le service est bien dans la mesure qu'on prend en charge gratuitement les enfants	IT,F,LE,F,F,A,RC,H,LE	2,2,1,1,2,2,1,2,3

Tableau 17: Description des boosters, Bras 1: Gao mars 2020

BOOSTERS	Arguments	Source	Méthode
Perception des services PCIMA	C'est un bon programme de lutte contre la malnutrition, Ce programme diminue le risque de décès chez les enfants de 6 à 59 mois. Il y a la disponibilité des intrants qui permettent de prendre en charge les enfants sans interruption	IT,F,A,H	2,1,2,1,3
Sensibilisation communautaire et dans les CSCOM	le RC fait la sensibilisation chaque jour et à chaque fois quand il y 'a la campagne de de masse de vaccination, Les sujets abordés sont; le sevrage, maladie infantile, les IRA, Selon les DTC, la sensibilisation se fait dans les CSCOM	IT,IT,F,F,F,RC	2,2,1,2,1,2
Connaissance de la malnutrition	Les symptômes de la malnutrition sont les yeux enfoncés, ballonnement du ventre, perte du poids, cheveux roux, sevrage précoce, le nom local de la malnutrition est "zankayalafanta" en Sorhain ou "yalafou"	IT,F,F,A,RC,H	2,2,1,2,2,1,3
Connaissance maladie infantile	Le paludisme sévit pendant la saison pluvieuse, la diarrhée et la malnutrition, Ces pathologies sévissent pendant la période de pluie et froide	IT,F,F,F,F,RC,H,H	2,1,2,1,2,2,1,1,3
Formation	selon les DTC, ils ont reçu 3 formations en PCIMA dont 2 fois avec ACF et une fois avec MDM, Ces formations ont apporté une valeur ajoutée dans le traitement de la malnutrition	IT,RC	2,2
Connaissance des services PCIMA	Les enfants sont pris en charge gratuitement avec du plumpy nut, lait F100, F75, Ces médicaments sont efficaces car permettent à l'enfant de guérir rapidement	F,F,F,F,A,A,A,RC,H	1,2,1,2,2,2, 2,2,1,3

Tableau 18: Description des boosters, Bras 2: Gao mars 2020

**BOOSTERS**	**Arguments**	**Source**	**Méthode**
Formation	Le Relais a reçu 5 formations PCIMA par ACF et MDM et il est très satisfait de cette formation qui l'aide à bien faire son travail	RC,IT,F	2,2,1
Sensibilisation communautaire et dans les CSCOM	Les relais font les séances de sensibilisation dans la communauté et dans le CSCOM, La sensibilisation s'effectue lors des campagne de vaccination sur les thèmes: paludisme, hygiène de l'eau, assainissement, nutrition et le lavage des mains,	RC,IT,F,IT,F,F,f,H,H,RC,LE,LE,le,LE	2,2,2,2,1,2,1,2,1,2,1,2,2,1,3
Connaissance maladie infantile	Les maladies suivantes ont été citées: le paludisme en saison pluvieuse, la rougeole en saison sèche et la malnutrition de façon permanente	RC,H,IT,F,F,F; H,H	2,1,2,1,1,1,2,2,3
connaissance de la malnutrition	La malnutrition est appelée « Hounaygazanta » en « Sonrhai » et est caractérisée par l'amaigrissement, cheveux gris, œdème, le vomissement, l'alimentation non équilibrée qui provoque le décès de l'enfant, "zanka mazagneye"	RC,H,A,F,F,F,F,H,le?LE,le,A	2,1,2,2,1,1,1; 3 2,1,2,2,2
Connaissance du service PCIMA	Le relais est celui qui a informé la population à plusieurs reprise sur la prises en charge de la MAS dans les CSCOM, les hommes de la communauté ont cité : l'ATP, l'amoxicilline comme le traitement de la MAS	H,A,F,F,H,LE,LE	1,2,2,1,2,1,2,3
Perception service PCIMA	La communauté est ravie du service PCIMA car cela permet de traiter les enfants gratuitement,	H,IT,IT,F,H	1;2,2,1,2,3
Dépistage passif	Tous les enfants qui arrivent au centre de santé voient leur données anthropométriques mesurés	IT,LE,LE	2,1,2
Qualité des services	Les mères d'enfants sont satisfaits de l'accueil, le temps d'attente, salle d'attente,	A,LE,A	1,2,2,3

- **Barrières à l'accessibilité**

Le tableau ci-dessous montre la liste de principales barrières à la couverture qui ont été identifiés à la fin de la collecte après triangulation :

Tableau 19 : Description des barrières, bras Contrôle ACF Gao,mars 2020

BARRIÈRES	Arguments	Source	Méthode
Collaboration Cs réf-CSCOM-Communautaire	Pas de supervision réalisée par le DTC car il fait confiance à l'agent de nutrition, pas de communication entre le DTC et le RC lors des références. Les supervisions par le niveau supérieur est faible et il y a peu de temps concentré à la nutrition; Il y a aussi une non communication entre le CSCOM et le CSREF lors des références. Les références sont réalisées par le partenaire MDM et non par la structure étatique, La supervision est réalisée de façon irrégulière avec le superviseur Nut	IT,F,RC,H	2,1,2,1
Rupture de stock	La rupture des intrants au niveau du CSCOM dissuade les parents de revenir au CSCOM, Il y a principalement les intrants MAM et MAS qui font défaut. Dans certains villages (Gadaye) on parle de rupture de plus d'un mois. Le plus gros obstacle de la PCIMA est la rupture des intrants MAM et MAS. La rupture de stock crée les abandons	IT,F,A,RC,RC,H	2,1,2,2, 2,1,3
Distance	Les longues distances plus de 10km empêchent certains personnes de se rendre au CSCOM, les familles partent en général à pied au CSCOM et n'ont pas de moyen financier pour le transport	IT,F,A,RC,LE	1,2,2, 2,2,3
Rejet	Les enfants sont rejetés à cause du non-respect des critères d'admission ou de rupture de stock alors qu'ils sont à au moins 15km. Dans certaines situations, l'enfant est rejeté à cause du manque d'argent pour payer les soins (Gadeye Njawo), Il n'y a pas suffisamment de chaises assisses pendant l'attente.	IT,F,F,A,RC, RC,LE, LE,H	1,2,1,2, 2,2,2,1,1
Pouvoir décisionnel	c'est le père qui prend la décision pour le déplacement de son enfant et la mère est encouragée par le RC	A,A,A,RC,RC, RC,LE,LE,H	2,2,2,2, 2,2,1,2,2,3
Abandon	Les RC ne sont pas au courant des abandons, Il n'ont jamais fait le suivi des abandons. Le manque de moyen financier pour le transport oblige certains parents à abandonner.	RC,H,LE,H	2,1,2,1
dépistage actif	Les RC identifient les enfants tous les 3 mois lors des activités de compagne de vaccination ou de dépistage de masse. Le dépistage de routine mensuellement est inexistant. La croix rouge malienne organise des dépistages massifs tous les 3 mois	IT,F,F,A,A, RC,RC,H	2,2,1,2, 2,2,2,1,3

Itinéraire thérapeutique	Certaines cibles ont parlé du traitement traditionnel à base du lait de chameaux et de l'argile sur la tête à cause de la longue distance de 20km . Une proportion de personne proche des CSCOM y parte dès qu'ils sont malades dans l'optique d'avoir un traitement rapide,	IT,F,F,F,A,A, A,RC,LE,RC,H	2,2,1,1,2, 2,2,2,2,2,1,3

Tableau 20 : Description des barrières, Bras 1, ACF Gao,mars 2020

BARRIÈRES	Arguments	Source	Méthode
Collaboration DTC-Communautaire	Les réunions mensuelles ne se réalisent pas car ce sont les superviseurs des ONG qui organisaient la réunion mensuelle. Il n y a pas de communication fluide entre le CSCOM et le CSREF ou entre le CSCOM et les RC	IT,F,A,RC,LE	2,1,2,2,1
Abandon	Il y a des abandons qui sont dus à l'enclavement car les patients doivent traverser les fleuves. Parfois il y a des déplacements de la population.	IT,F,F,H,LE	2,1,2,1,2
Accueil	Le mauvais accueil des patients et l'absence des hangars ou salle d'attente occasionnent la longue attente avant la prise en charge	IT,F,A,LE	2,1,2,2
rejet	Plusieurs rejets ont été observés sans connaissance de la raison, parfois cela est dû au rupture de stock	F,F,H,Le	2,1,1,2
Itinéraire thérapeutique	Certains mères essayent les herbes à cause des longues distances dès que l'enfant est malade, ou l'automédication et l'amène à l'hôpital quand l'enfant ne guéri pas	IT,IT,F,F,F,A	2,2,2,1,2,2,3
Distance	La distance(>5km) empêche certaines personnes d'aller directement au CSCOM quand ils sont malades d'où ils utilisent les décoctions traditionnels (herbe+piment+ gingembre) pour se soigner. Le manque de moyen financier pour le transport est aussi un élément crucial	F,F,F,A, LE	1,2,1,2,2,3
pouvoir décisionnel	C'est le père qui est la seule personne capable de prendre des décisions et encouragé parfois par le RC. La décision peut revenir à mon premier fils car c'est lui qui prend en charge la famille	F,A,A,RC,LE	1,2,2,2,1,3
Dépistage actif	Les RC, font le dépistage par trimestre en prenant le PB et en cherchant les œdèmes	IT,F,F,F,RC,H	2,2,1,1, 2,1,3

Tableau 21 : Description des barrières, Bras 2 ACF Gao,mars 2020

BARRIÈRES	Arguments	Source	Méthode
Pouvoir décisionnel	Il n y a que le père qui puisse prendre la décision dans une famille car il est le responsable de sa famille,	RC,A,LE,A	2.2,1,1,3
Distance	Les longues distances empêchent les parents d'arriver au CSCOM malgré qu'ils soient référés par les relais. Les dunes de sable sont aussi une des causes	RC,F,LE	2,1,2,3
Abandon	Les enfants MAM ont abandonné à cause de la rupture de stock et la distance. Parfois les abandons sont dus au déplacement de la population, et l'enclavement	H,F,LE	2,1,2
Rupture de stock	Il y a une rupture des intrants qui empêche les enfants d'aller dans les structures de santé et de même que le dysfonctionnement des balances	H,IT,LE	2,2,1,3
Itinéraire thérapeutique maladie infantile	Les malades font soit l'automédication ou partent chez les tradipraticiens en première intention. Après l'échec ils se rendent au CSCOM	RC,H,A,F,F,F,F,LE,le,le	2,1,2,2,1,1,1,2,2,2,3
Dépistage	Le dépistage actif se réalise par trimestre chez les enfants de 6-59 mois,	RC,IT,F,IT,f,F,F, H,H,RC,LE,LE,le	2,2,2,2,1,2, 1,2,2?1,1,1,1,3
Collaboration CSCOM-Communauté	Le relais est supervisé par le responsable de nutrition du CSCOM une fois par semaine. Chaque le relais communautaire assiste irrégulièrement à une réunion de monitorage une fois par mois pour compiler les données et identifier les insuffisances. Pour les DTC ils sont supervisés irrégulièrement	RC,IT,LE	2,2,1

V. DISCUSSION

Le projet ICCM+ dans les 3 bras a pour but d'intégrer les traitements MAS au niveau communautaire (ASC) dans le bras 1 et le bras 2 à l'aide du protocole standard et simplifié de la PCIMA. L'enquête de couverture de mars 2020 dans le bras contrôle, bras 1 et bras 2 visaient à estimer la couverture baseline des traitements MAS et MAM et l'identification des barrières et booster. La triangulation de toutes les donnes qualitatives et quantitatives dans l'enquête, nous donne un résultat avec un reflet très fiable de la situation dans le district de Gao.
Les résultats de l'évaluation de la couverture dans les 3 bras (contrôle, bras 1 et bras 2) aboutissent à une estimation la couverture unique qui est ainsi estimée :

	BRAS CONTROLE		BRAS 1		BRAS 2	
	MAS	MAM	MAS	MAM	MAS	MAM
**Couverture unique**	20,5%	9,8%	25,0%	13,9%	6,3%	12,1%
**Intervalle de confiance**	12,0%-28,9%	6,4%-13,2%	13,7%-36,3%	8,2%-19,5%	1,4%-11,2%	6,8%-17,3%

Les résultats de l'enquête montrent une couverture unique des MAS, MAM largement en dessous de 50% qui est le seuil recommandé par les normes sphères en zone rurale. Cette faible couverture est due à un manque des moyens financiers pour le déplacement, la rupture de stock et un faible dépistage actif. Cette couverture est en générale plus faible pour les MAM à l'exception du bras 2. Ce faible taux comparativement entre les MAM et MAS du bras 2 est à pondérer avec la recrudescence de l'insécurité dans la zone limitant les déplacements de la communauté vers les CSCOMS et sa vulnérabilité géographique. De plus, les villages éloignés ou qui se trouvent isolés sont les endroits où la couverture est plus faible. La cause est due au fait que 70% des MAS se trouvent dans la plupart des villages très éloignés(>15km) des CSCOM ou les villages au bord du fleuve alors que ceux des MAM sont plus proches des CSCOM(<5km). La couverture est plus faible dans les bras 2 par rapport aux bras 1 et contrôle principalement à cause de l'éloignement de la plupart des villages des lieux des soins.

En analysant les données du dépistage des enfants MAM et MAS, on constate qu'en moyenne 85,9% des non couverts n'ont pas été dépistés le mois antérieur à l'enquête.

En faisant une analyse approfondie des données du dépistage des enfants MAM et MAS non couverts par rapport aux critères d'admission versus critères de dépistage dans un programme de PCIMA fonctionnant correctement, on obtient des résultats très intéressants. On observe dans le bras contrôle pour la MAS que 55,6% devraient être détectés par le PB/œdème et 44,4% exclusivement par le PT. Pour la MAM non couvert, 71,2% devraient être détectés par PB et 28,8% par le PT exclusivement. Or tous ces enfants n'ont pas été dépistés le mois antérieur à l'enquête et ont été identifiés pendant l'enquête.
Dans le bras 1 parmi les non couverts dans un programme de PCIMA fonctionnant correctement, on observe pour la MAS que 74,3% devraient être détectés par le PB/œdèmes et 25,7% exclusivement par le PT. Pour la MAM non couvert on a 56,3% qui devraient être détectés par PB et 43,7% exclusivement par le PT.
Dans le bras 2 pour les cas non couverts MAS si le programme PCIMA fonctionnent correctement, 32,7% devraient être détectés par le PB/œdèmes et 63,6% exclusivement par le PT. Pour la MAM non couvert on a 36,4% qui devraient être par PB et 36,4% exclusivement par le PT.
L'ensemble de ces résultats pour les patients non couverts et malnutris montrent qu'on n'arrive pas à identifier tous les enfants malnutris au niveau communautaire en tenant compte du fait que le PB/œdème est souvent utilisé par les relais pour faire le dépistage. De plus, le PT est utilisé pour le triage des enfants au niveau des CSCOM, le critère PT au niveau des différents bras montre que les triages ne sont pas totalement efficaces dans la mesure que les enfants n'arrivent pas au CSCOM et qu'en général on devrait envoyer les enfants supposés MAM et MAS au CSCOM pour un triage. Le non dépistage des enfants couverts dans la communauté montre un non fonctionnement de la surveillance nutritionnelle. En somme, le système de dépistage communautaire mensuel est presque non fonctionnel. Par ailleurs, les dépistages de masse seraient réalisés trimestriellement lors des campagnes de vaccination ou par la croix rouge malienne de façon irrégulière. Le dépistage actif n'est pas fait par tous les relais communautaires et certains ne sont plus motivés. Les relais communautaires sont en général actifs dans les zones semi urbaines. Le dépistage actif déficient, l´insuffisance de moyens de travail des Recos et leur manque de motivation fait que la faiblesse des activités communautaires en général sont un des facteurs qui empêchent l'augmentation de la couverture. Il est aussi important de rappeler que le volet communautaire constitue la pierre angulaire d'un programme PCIMA. Ainsi dit, la réussite d'un programme PCIMA passe par une bonne performance des agents de santé communautaires. La motivation de ces derniers doit faire partir d'une préoccupation prioritaire si l'on s'attend à une bonne couverture du programme dans les deux bras expérimentaux. L'idéal serait de susciter au sein de l'équipe des relais communautaires l'esprit de volontariat ou de fierté de servir leur propre communauté et pas toujours dépendre de la prime qu'ils reçoivent. Le travail mené par les relais communautaires nécessite un suivi rapproché bien qu'il y ait nécessité d'augmenter leur nombre, il faut d'abord encadrer ceux qui existent déjà afin d'améliorer leur performance dans le travail.

Au niveau des formations sanitaires, la détection des cas souffre d'insuffisances car on a des pourcentages élevés de détection par PT chez les MAM et MAS non couverts. Cet aspect du dysfonctionnement du dépistage passif a été constaté lors de l'enquête qualitatives et confirmé par plusieurs cibles et sources. Le dysfonctionnement du dépistage actif est accentué par un non suivi de cette activité par le CSCOM et se caractérisant par une supervision et réunion irrégulières.

Les ruptures de stock de produits nutritionnels et de médicaments au niveau du CSCOM ont également occupé une place prépondérante dans les motifs de non-fréquentation évoqués par les accompagnants des cas non couverts. Dans les trois bras pour les enfants non couverts, on observe en moyenne 54,3% des cas MAM non couverts (bras contrôle=56,4%, bras 1=82,1%, bras 2=59,2%) et 50,3 %des cas MAS non couverts (bras contrôle=51,9%, bras 1=66,7%, bras 2=33,3%) ont évoqué le problème de rupture de stock. Cette rupture de stock est plus prononcée dans le bras 1. Elle se caractéris par le rejet des enfants référés pendant l'enquête. Ceci explique la difficulté d'avoir accès au CSCOM et d'avoir un traitement approprié. Le renvoi des bénéficiaires à cause des ruptures de stock a un effet déstabilisateur très important sur la couverture et des conséquences graves pour la santé de l'enfant. Parmi les raisons d'abandon du projet on observe lors de l'enquête qualitative que le rejet des enfants à cause de la rupture de stock est ressorti dans les 3 bras. Sans les produits de base nécessaires, il est impossible pour un CSCOM de dispenser des soins. Par conséquent, les ruptures de stock au niveau des CSCOM et non du district ont probablement affecté les résultats de l'étude.

La majorité des accompagnants qui ont cité le problème de manque des moyens financiers pour le déplacement dans les 3 bras confondus était 8,1% pour les cas MAM non couverts (bras contrôle=5,5%, bras 1=7,1%, bras 2=11,8%) et 33,0% pour les cas MAS non couverts (bras contrôle=32,7%, bras 1=23,8%, bras 2=42,4%). Un manque de moyens financiers pour le déplacement prouve qu'il y a un problème de distance et d'insécurité de telle manière que les accompagnants ne peuvent pas aller à pied au CSCOM (>8,5 km). Ainsi, le manque des moyens financiers pour le déplacement/distance limite les déplacements vers les CSCOM. Bien que la prise en charge soit gratuite et que la majorité de la population a conscience du système de gratuité des soins dans le DS de Gao, les mères des villages éloignés de plus de 8,5 km ou vivant dans les zones proches du fleuve (zones inondables) ne peuvent payer le prix d'un transport. La longue distance se caractérise avec une moyenne de 8,5 km pour les trois bras. Ainsi, le manque des moyens financiers pour le déplacement/distance empêcherait certains enfants d'avoir accès aux soins nutritionnels appropriés.

Les résultats de l'enquête montrent qu'environ 45,3% des accompagnants (bras contrôle=54,2%, bras 1=36,5%, bras 2=45,2%) ne connaissant pas la PCIMA. Cette non connaissance de la PCIMA est plus accentuée dans le bras contrôle et le bras 2. Cette raison souligne l'importance du travail de proximité avec la communauté et indiquent que celle-ci n'a pas lieu aussi régulièrement qu'elle le devrait. Cette raison est également un indicateur d'un manque de compréhension des membres de la communauté à l'égard de la PCIMA, ce qui montre un manque d'activités de proximité menées par les relais communautaires dans certaines communautés. Le manque de connaissance est souvent la cause la plus importante de la faible couverture du programme de prise en charge nutritionnelle. Cette non connaissance de la PCIMA peut diminuer les possibilités de référencement spontané en dehors des séances de dépistage.

Les personnes qui s'occupent d'enfants malades semblent préférer pour 19,6%(bras contrôle=20,8%, bras 1=26,2%, bras 2=11,9%) des accompagnants aller en première intention chez les tradipraticiens. Par ailleurs, 27,2% (bras contrôle=16,7%, bras 1=55,9%, bras 2=9,1%) des accompagnants font de l'automédication en première intention quand l'enfant est malade. Ces médicaments en provenance de vendeurs ambulants sont également utilisés en première intention en raison de sa disponibilité (vendeurs se déplacent dans les villages) et de son caractère peu onéreux. Le fait d'aller chez le tradipraticien serait probablement du au manque de moyen financier pour le déplacement et la recrudescence de l'insécurité dans la zone limitant les déplacements de la communauté vers les CSCOMS. Mais cela indique que même si les distances entre les communautés et les centres de santé communautaires les plus proches sont grandes, certains accompagnants semblent disposés à se rendre aux centres de santé communautaires sous la recommandation du relais communautaire. En effet, le recourt aux tradi-thérapeutes est très recherché pour le traitement traditionnel ainsi que des moyens de protection des maladies (talisman et lavage avec des décoctions). Les tradi-thérapeutes font partie de la communauté, sont reconnus et ont la confiance de la population. Le prix des consultations est souvent modique et adapté aux ressources du foyer. Par la suite, en cas de non amélioration de l'état de santé de l'enfant, les familles se rendent aux centres de santé communautaires pour 31% des accompagnants quelque soit le bras. Les personnels de santé ont confirmé qu'une très grande majorité des enfants malnutris a déjà reçu à domicile d'autres traitements avant leur admission dans le programme. Cette attitude crée un retard de traitement qui est aussi du au fait que 79,9% des pères doivent prendre la décision pour que la mère puisse aller au CSCOM. En absence du père, il serait parfois difficile pour la mère d'aller au CSCOM avec l'enfant

Même si l'analyse des données quantitatives et qualitatives ont relevé plusieurs facteurs négatifs en rapport avec l'organisation de la PCIMA dans ces trois bras, des facteurs positifs ci-dessous permettent d'avoir les couvertures estimées. Il s'agît du fait que les infirmiers diagnostiques correctement la maladie de l'enfant et les encouragements des relais communautaires vis-à-vis des mères d'aller dans les CSCOM et de suivre le traitement jusqu'à la guérison. A tout ceci, la gratuité joue aussi un rôle non négligeable favorisant l'accessibilité aux soins. En moyenne 72,5% des accompagnants ont une perception de la PCIMA comme un endroit où on traite les enfants malnutris correctement. Les sensibilisations communautaires, la connaissance des maladies infantiles (85,7% des accompagnants) et la qualité des services ont joué un rôle non négligeable dans les couvertures uniques obtenues.

VI.RECOMMANDATIONS

L'évaluation de la couverture dans les trois bras a permis d'identifier des blocages dans l'exécution du programme de PCIMA. Après discussion avec les différents partenaires étatiques les recommandations ci-dessous et les activités appropriées pour résoudre les problèmes soulevés par l'évaluation sont élaborées. La liste des recommandations est incluse dans le tableau ci-après.

Thème	Défi	Recommandation	Responsable	délais
Dépistage communautaire et référence	On constate qu'en moyenne 85,9% respectivement des non couverts n'ont pas été dépistés le mois antérieur à l'enquête.	• Mettre en place dans tous les villages des trois bras la stratégie PB ménage<br>• Veiller à ce que les relais communautaires puissent être actif dans tous les villages des trois bras<br>• Elaborer une data base de dépistage en tenant compte de l'aspect géographique (prendre en compte l'ensemble de tous les villages dans les bras et faire un suivi mensuel du dépistage.<br>• Organiser mensuellement les réunions entre les relais communautaires et les CSCOM par trimestre et entre les RC et les ASC par mois<br>• Renforcer le dépistage passif dans les CSCOM	Chef de projet et superviseur	Début opérationnel du projet
	Les accompagnants ne peuvent pas se rendre au CSCOM à plus de 8,5 km	• Améliorer la mobilisation et la sensibilisation dans la communauté.<br>• Décentraliser les soins au niveau communautaire<br>• Encourager les autres membres de la famille à accompagner les mères dans les postes de santé<br>• Mettre en place une stratégie mobile et avancée dans les bras 1 et 2 afin d'atteindre les enfants situés à une grande distance des structures sanitaires et des villages<br>• Payer le transport des accompagnants avec malnutrition aigüe sévère avec complications médicales		
Connaissance de la malnutrition	Compréhension limitée de la malnutrition	• Utilisez des groupes de soutien pour vulgariser les bonnes pratiques et la connaissance de la malnutrition<br>• Comprendre et utiliser les termes locaux pour désigner la malnutrition	Chef de projet et superviseur	Début opérationnel du projet

	aiguë, ses symptômes et implications des accompagnants	• Sensibilisation accrue des mères et pères sur les bonnes pratiques en nutrition<br>• Faire la sensibilisation sur le fait que les ATP sont des médicaments et ne doivent pas être partager<br>• Impliquer les chefs de villages et dignitaires religieux dans la sensibilisation		
Gestion du stock	La rupture de stock est permanente au niveau des CSCOM	• Faire un plaidoyer auprès d'Unicef, PAM et la région<br>• Passer les commandes un mois avant la fin du stock actuel,<br>• Aider à transporter les intrants dans les CSCOM	Chef de projet et superviseur	Début opérationnel du projet

ANNEXES

Annexe 1

Bras contrôle

ENQUETE COUVERTURE GAO 2020				MAM						Enfants MAS							
**VILLAGE**	**BEN**	**MAM**	**MAS**	**PB**	**PT**	**PT et PB**	**MAM couvert**	**MAM en voie de guerison**	**MAM non couvert**	**OEDEME**	**PB**	**PB et Oedem**	**PT**	**PT et PB**	**MAS couvert**	**MAS en voie de guerison**	**MAS non couvert**
Alfalikana	77	19	7	5	8	6	1	4	14		1		4	2		2	5
Aljanabandia Centre	93	15	4	7	5	3	3		12			1	2	1	1	1	2
Koulsouk	43	1	4		1				1		1		2	1			4
Thianboukona		16	14	7	1	8			16		5		3	6	1		13
Zaba	21	3			3			1	2								
Djoula Bouguey diando	21	3	1		3			2	1					1			1
Djoula Issabero	39	7	2	4	3			2	5	1				1	1		1
Saney Ecole	41	3	1	1	2			1	2		1						1
Saneye B		12	6	5		7			12		1		1	4			6
Gadeye	212	33	16	11	12	10		3	30	1	7		5	3	3	2	11
N'jawa	84	3	1	1	2			2	1				1				1
N'kondo	7																
Tandagari	12	4	1		3	1		2	2				1				1
Thionboukona	101	4	4	1	2	1			4	1	1			2	2	1	1
Karamigna	117	26	6	17	3	6	3	1	22		4		1	1	1	3	2
Infardane	29	5		2	1	2			5								
Intagate	103	4		1	2	1		1	3								
Kel Tondibi	17	1		1					1								
Makara	76	9	4		7	2		3	6				4				4
Tin Aouker	54		1										1				1
	**1147**	**168**	**72**	**63**	**58**	**47**	**7**	**22**	**139**	**3**	**21**	**1**	**25**	**22**	**9**	**9**	**54**

Bras 1

				MAM						Enfants MAS						
**VILLAGE**	**BEN**	**MAM**	**MAS**	**PB**	**PT**	**PT et PB**	**MAM couvert**	**MAM en voie de guerison**	**MAM non couvert**	**OEDEME**	**PB**	**PT**	**PT et PB**	**MAS en voie de guerison**	**MAS couvert**	**MAS non couvert**
Akalahondia Ile	78	4	2	3		1		1	3		1		1	1		1
Gabame Gourma	65	3	1	2	1		1	1	1				1			1
Garbame	196	12	3	7	1	4	4	3	5		1	1	1	1	2	
Jamey kounssoume	80	8	2	3	3	2	2		6		2				1	1
Korgahaye Ile	39		3								3					3
Gorom Gorom	101	6	2	1	4	1			6	1		1		1		1
Koïma	61	5	13	2		3			5		6	1	6			13
Lobou	232	16	2	8	5	3	1	1	14				2	1		1
Sidibe	126	11	3		8	3			11		1		2	1	1	1
Banikane	68	6	4	5		1			6		1	1	2			4
Borno	44	13	2	3	6	4			13		1	1				2
Dongome	45	3				3			3							
Kardjime	66	1	1		1				1			1			1	
Koissa I	38	2		1		1			2							
Marga	75	9	2	5	2	2	3	2	4		1	1		1	1	
Tianame	174	18	6	3	13	2			18		1	4	1		2	4
Traore	108	4	2		2	2			4			2				2
Zinda	65	2	1	1	1		1		1			1				1
	**1661**	**123**	**49**	**44**	**47**	**32**	**12**	**8**	**103**	**1**	**18**	**14**	**16**	**6**	**8**	**35**

Bras 2

				MAM						Enfants MAS						
**VILLAGE**	**BEN**	**MAM**	**MAS**	**PB**	**PT**	**PT et PB**	**MAM couvert**	**MAM envoie de guerison**	**MAM non couvert**	**Oedeme**	**PB**	**PT**	**PT et PB**	**MAS en voie de guérison**	**MAS couvert**	**MAS non couvert**
Bazi	114	11	7	3	3	5	3	1	7			4	3	4		3
Bolongo	64	3	1		2	1			3			1				1
Doussougou	74	12	6	3	6	3			12		2	4				6
Doye	40	3			3				3							
Goudele Baria	85	12	9	4	4	4	6	2	4		3	6				9
Koigourame	10	5			5		2		3							
Oura Migno	112	5	5	2	2	1			5			3	2	1	1	3
Silwali	70	5	2	1	1	3	2		3			2				2
Woybotolia	31	3	3	1	1	1	1	1	1			3				3
Adoulanbo	47	1		1					1							
Bandiakara	63	15	2	5	2	8			15		1		1			2
Barguey	33															
Baringouma	61	12	1	4	4	4			12		1					1
Djidal	42	4			2	2			4							
Kokorom	32	5	2	4		1			5			2				2
Magnadoue Bero	95	4	4	1	1	2			4		2	2				4
Mologouria	76	3	1	2		1			3				1			1
Sabeye Boba	2		3									3				3
Saya koira	21															
Seyna Haoussa	111	7	1		6	1			7			1				1
Tanal	56	4		3	1				4							
Thiarembou jena	67	18	5	9	6	3			18	1		3	1			5
Tondeyga	16															
Toutoulberi	55	6	2	1	1	4			6		2					2
Troumé	38	1	1	1					1				1			1

	**1415**	**139**	**55**	**45**	**50**	**44**	**14**	**4**	**121**	**1**	**11**	**34**	**9**	**5**	**1**	**49**

1 RÉFÉRENCES

[i] UNICEF, WHO & WBG. (2019). Levels and trends in child malnutrition. Key findings of the 2019 edition. Disponible sur : https://www.who.int/nutgrowthdb/estimates2018/en/
