# Supplementary material for: Effectiveness of decentralizing outpatient acute malnutrition treatment with community health workers and a simplified combined protocol: a cluster randomized controlled trial in emergency settings of Mali
Source: Front Public Health. 2024 Feb 21;12:1283148. doi: 10.3389/fpubh.2024.1283148 (PMC10915236; doi:10.3389/fpubh.2024.1283148)
Supplement: Supplementary file 3 [file Presentation_2.pdf]

ENQUETE DE COUVERTURE

NOM DU PROJET : « Efficacité, coût-efficacité et couverture du traitement de la malnutrition aigüe sévère délivré par les agents de santé communautaire à travers le protocole modifié dans des contextes d'urgence au Mali »

LOCALISATION : Région de Gao-Mali DATE

D'ENQUÊTE : Mai-Juin 2021

AUTEURS : Abdias Ogobara Dougnon, Cisse Boiré et Hugh Lort-Phillips.

TYPE D'ENQUÊTE : Couverture à grande échelle

TYPE DE PROGRAMME : MAS et MAM dans les CSCOM et dans la communauté

ORGANISATION EXECUTANTE : Action Contre la Faim

BAILLEUR : ELRHA

TABLE DES MATIERES

LISTE DES FIGURES ... ii
LISTE DES TABLEAUX ... iii
ACRONYMES ... iv
REMERCIEMENTS ... v
RESUME ... vi
I. CONTEXTE ET JUSTIFICATION ... 2
II. OBJECTIFS ... 5
1. Objectif général ... 5
2. Objectifs spécifiques ... 5
III. METHODOLOGIE ... 5
1. Domaine couvert par l'enquête ... 5
2. Echantillonnage et plan de l'enquête ... 5
2.1. Population d'étude ... 5
2.2. Base de sondage ... 6
2.3. Taille de l'échantillon ... 6
2.4. Sélection des grappes ... 7
2.5. Sélection des ménages ... 8
2.6. Sélection des sujets ... 9
2.7. Procédure d'échantillonnage pour la recherche des raisons pour les cas couverts et non couverts ... 9
3. Préparation de la collecte des données ... 9
3.1. Données anthropométriques ... 9
3.2. Estimation de la couverture avec intervalle de confiance à 95% ... 11
3.3. Ressources Humaines de l'enquête ... 13
3.4. Formation des enquêteurs ... 14
3.5. Standardisation des mesures ... 14
3.6. Traitement et analyse des données ... 15
4. Organisation sur le Terrain ... 15
5. Limites de l'enquête ... 16
6. Problèmes rencontrés ... 16
7. Considérations éthiques ... 16
IV. RESULTAT ... 17
1. Description de l'échantillon ... 17
2. Estimation de la couverture ... 18
V. DISCUSSION ... 29
VI. CONCLUSION ... 31

LISTE DES FIGURES

Figure 1: Réponse à la question: Votre enfant a-t-il été dépisté à la maison par un ruban PB avant ? (« n » est égal au nombre de répondants dans chaque bras de l'étude) 23
Figure 2: Pour les accompagnants qui ont confirmé que leur enfant a été dépisté par un PB précédemment, la période depuis de la mesure 23
Figure 3: Zone contrôle : Les raisons principales pour lesquelles les accompagnants des cas MAM et MAS couverts ont inscrit leur enfant dans le programme PCIMA 24
Figure 4: Bras 1 : Les raisons principales pour lesquelles les accompagnants des cas MAM et MAS couverts ont inscrit leur enfant dans le programme PCIMA 24
Figure 5: Les raisons principales pour lesquelles les accompagnants des cas MAM et MAS couverts ont inscrit leur enfant dans le programme PCIMA 25
Figure 6: Zone contrôle : Les raisons principales pour lesquelles les accompagnants des cas MAM et MAS n'ont pas amené leur enfant au CSCOM 26
Figure 7: Bras 1 : Les raisons principales pour lesquelles les accompagnants des cas MAM et MAS n'ont pas amené leur enfant au CSCOM et/ou site ASC 26
Figure 8: Bras 2: Les raisons principales pour lesquelles les accompagnants des cas MAM et MAS n'ont pas amené leur enfant au CSCOM et ou site ASC 27

LISTE DES TABLEAUX

Tableau 1: Les critères de traitement dans les trois bras du projet ICCM+ sont inclut dans le tableau ci-dessous ... 3
Tableau 2: Prévalence de la malnutrition aigüe sévère à Gao, enquête SMART, Décembre 2020 .7
Tableau 3: Calculs de la taille d'échantillon pour les cas MAS pour chaque bras de l'étude 7
Tableau 4: Définitions des cas MAS et MAM dans les zones d'intervention et contrôle, Mai 2021 ...10
Tableau 5: Définitions des cas MAS et MAM dans les zones d'intervention et contrôle, Mai 2021 ...10
Tableau 6: Définitions des cas couverts et non couverts dans les zones d'intervention et contrôle, Mai 2021 ... 11
Tableau 7: Synthèse des données collectées pendant l'enquête a grande échelle dans Gao, mai 2021... 17
Tableau 8: Comparaison taille de l'échantillon attendue et atteinte dans les zones d'intervention et contrôle, mai 2021 ... 17
Tableau 9: Cas MAS identifiés selon les critères d'admission... 18
Tableau 10: Cas MAM identifiés selon les critères d'admission ... 18
Tableau 11: Estimations de couverture du programme de traitement MAS dans les trois bras de l'étude ...18
Tableau 12: Estimations de couverture du programme de traitement MAM dans les trois bras de l'étude ...19
Tableau 13: Classification de traitement MAM et MAS par aire de santé dans le bras Contrôle (classification selon la couverture de période)... 19
Tableau 14: Classification de traitement MAM et MAS par aire de santé dans le Bras 1 (classification selon la couverture de période)... 20
Tableau 15: Classification de traitement MAM et MAS par aire de santé dans le Bras 2 (classification selon la couverture de période)... 20
Tableau 16: Estimations de couverture pour les cas MAS selon le Critère 1 (Par PB uniquement ou par PB+P/T)... 21
Tableau 17: Estimations de couverture pour les cas MAS selon le Critère 2 (Par P/T uniquement) ...21
Tableau 18: Estimations de couverture pour les cas MAM selon le Critère 1 (Par PB uniquement ou par PB+P/T)... 22
Tableau 19: Estimations de couverture pour les cas MAM selon le Critère 2 (Par P/T uniquement) ... 22

ACRONYMES

**ACF**	Action Contre la Faim
**ASC**	Agent de Santé Communautaire
**ATPE**	Aliment Thérapeutique Prêt à l'Emploi
**CSCOM**	Centre de Santé Communautaire
**DTC**	Directeur Technique du Centre
**ERGg**	Essai randomisé contrôlé par grappes
**ICCM**	Integrated Community Case Management
**INSP**	l'Institut National Santé Publique
**MAG**	Malnutrition Aigue Globale
**MAM**	Malnutrition Aigue Modérée
**MAM C**	Malnutrition Aigue Modérée Couvert
**MAM EVDG**	Malnutrition Aigue Modérée en Voie de Guérison
**MAM NC**	Malnutrition Aigue Modérée Nouveau Cas
**MAS**	Malnutrition Aigue Sévère
**MAS C**	Malnutrition Aigue Sévère Couvert
**MAS EVDG**	Malnutrition Aigue Sévère en Voie de Guérison
**MAS NC**	Malnutrition Aigue Sévère Nouveau Cas
**MEAL**	Suivi, évaluation, redevabilité et apprentissage
**ODK**	Open Datta Kit
**OFDA**	Office for US Foreign Disaster Assistance
**OMS**	Organisation Mondiale de la Santé
**PB**	Périmètre Brachial
**PCIMA**	Prise Charge Intégrée de la Malnutrition Aigue
**PCIME-C**	Prise en Charge Intégrée des Maladies de l'Enfant ay niveau Communautaire
**PT**	Poids/Taille
**SLEAC**	Evaluation de l'Accessibilité et de la Couverture à base de LQAS (Lot Quality Assurance Sampling) simplifié
**SMART**	Standardized Monitoring and assessment of Relief and Transition
**UK**	Royaume-Uni
**UNICEF**	Fonds des Nations Unies pour l'enfance

REMERCIEMENTS

Nos remerciements sont adressés à toutes les personnes qui ont rendu possible la réalisation de cette évaluation : aux autorités administratives et sanitaires, au personnel des structures de santé, ainsi qu'aux communautés visitées pour leur collaboration et participation active.
Merci également à toute l'équipe d'Action Contre la Faim qui a rendu cette évaluation possible grâce à l'appui financier, aux Districts pour leur participation active, constructive et enthousiaste tout au long de l'évaluation, ainsi qu'aux enquêteurs, enquêtrices et superviseurs pour la qualité de leur travail et leur motivation, sans qui ce travail n'aurait pu être réalisé.

L'évaluation a été menée par l'équipe d'Action Contre la Faim Mali

RESUME

Cette évaluation de la couverture a été menée dans le bras contrôle (protocole standard au centre de santé), bras 1 (protocole standard au centre de santé et site ASC) et bras 2 (protocole simplifié au centre de santé et site ASC) dans le district de Gao au Mali. Ces 3 bras ont été tirés par randomisation parmi les villages de l'ensemble du district dans le cadre de l'évaluation de base « End line » du projet ICCM+ (Integrated Community Case Management) financé par OFDA et ELRHA. Ce projet a pour but de décentraliser la prise en charge de la malnutrition aigüe au niveau de la communauté en s'appuyant sur les agents de santé communautaire (ASC) et de voir s'il y'a une augmentation de la couverture, l'efficacité et le cout-efficacité de la prise en charge des MAS dans le bras 1 et bras 2 durant la période de juillet 2020 à juin 2021. Cette enquête de couverture a été réalisée du 25 Mai au 11 Juin 2021 et a pour objectif d'évaluer la couverture finale des traitements MAS et MAM chez les enfants âgés de 6 à 59 mois dans les bras d'intervention (bras 1 et bras 2) et le bras contrôle de l'étude dans le district sanitaire de Gao.

L'enquête de couverture a été réalisée dans le bras contrôle et le bras d'intervention (bras 1 et bras 2) en utilisant une méthodologie adaptée « d'enquête de couverture sur une grande zone ». Les tailles de l'échantillon des cas dans les trois bras de l'étude ont été calculée à partir du calculateur Sampsize[1] indiqué pour ce type d'enquête. Les villages ont été sélectionnés par la méthode de tirage aléatoire systématique pour chaque bras. Ensuite les cas ont été identifié selon leurs mesures anthropométriques. Selon les totaux des cas identifiés dans chaque bras de l'étude, il a été possible d'estimer la couverture de traitement de la malnutrition aigüe sévère (MAS) et de la malnutrition aigüe modéré (MAM) chez les enfants âgés de 6 à 59 mois en utilisant « l'estimateur de couverture unique ».

Les tailles d'échantillon pour MAM et MAS ont été atteintes dans les trois bras de l'étude. Les estimations de couverture sont les suivantes :

	Bras contrôle				Bras 1				Bras 2			
	Mars-20		Mai-21		Mars-20		Mai-21		Mars-20		Mai-21	
	MAS	MAM	MAS	MAM	MAS	MAM	MAS	MAM	MAS	MAM	MAS	MAM
Couverture Unique	20,50%	9,80%	18.9%	16.1%	25,00%	13,90%	38.5%	23.4%	6,30%	12,10%	48.8%	25.9%
Intervalle de Confiance	12,0%-28,9%	6,4%-13,2%	11.6% - 26.2%	11.7%-20.5%	13,7%-36,3%	8,2%-19,5%	31.1%-45.8%	19% - 27.9%	1,4%-11,2%	6,8%-17,3%	33.5% - 64.1%	17.9% - 33.8%

[1] http://sampsize.sourceforge.net/iface/index.html#prev

Les résultats de l'enquête montrent une couverture unique du traitement de MAS et MAM en dessous de 50% qui est le seuil recommandé par les normes sphères en zone rurale. Les estimations de couverture du programme MAS indiquent une augmentation de la couverture dans les Bras 1 et Bras 2 comparativement à l'enquête Baseline de mars 2020. La couverture de traitement de la MAS dans le bras « Contrôle » n'a pas évoluée pendant la mise en place l'étude. Mais les couvertures du traitement de la MAS dans Bras 1 et 2 peuvent être classifiées comme « Modérées » (entre 20 et 50%). Les estimations de couverture du programme MAM montrent également une augmentation de la couverture dans les bras d'intervention (Bras 1 et Bras 2) comparativement à l'enquête Baseline de mars 2020 mais nous n'avons eu aucun changement dans le bras Contrôle entre l'enquête Baseline et End-line. Les estimations de couverture pour les Bras 1 et 2 se trouvent dans la classification « modérée » parce qu'elles dépassent les 20%. Mais dans ces bras de l'étude, seulement un quart des enfants MAM ont été couverts par le traitement.

Dans les bras Contrôle et Bras 2, moins de 40% des accompagnant.e.s des cas de MAS et de MAM (dans le programme ou non) ont dit que leur enfant a été dépiste chez eux précédemment avant l'enquête. Ces informations indiquent que les activités communautaires sont limitées c'est-à- dire que les relais communautaires n'ont pas réalisé un dépistage actif (dépistage porte-à- porte) dans la plupart des villages des aires de santé de ces deux bras d'étude. Dans le Bras 1, 58% des accompagnant.e.s ont confirmé que leur enfant a été dépisté dans le ménage ce qui indique une mobilisation communautaire plus forte dans les aires de sante de ce bras d'étude. Pour les accompagnant.e.s qui ont confirmé le dépistage dans le ménage avec l'enquête, dans tous les bras (bras contrôle, bras 1 et 2) de l'étude la plupart ont dit que le relais communautaire a dépisté leur enfant 30 jours avant l'enquête de couverture. Ainsi, nous pouvons confirmer que dans plusieurs villages des différents bras d'étude, le dépistage des enfants parte-à porte n'est pas effectif. Mais dans les villages où le dépistage est effectif, il y a un dépistage régulier par les relais communautaires.

I. CONTEXTE ET JUSTIFICATION

Selon les estimations conjointes de l'UNICEF, de l'OMS et de la Banque mondiale sur la malnutrition (édition 2021), l'émaciation continuait à menacer la vie d'environ 6,7 % des enfants de moins de 5 ans dans le monde, soit 45,4 millions, plus d'un quart en Afrique 12,1 millions de enfants de moins de 5 ans (27%) et 6,4 millions d'enfants souffraient d'émaciation (sévère et modérée) en Afrique de l'Ouest et du Centre en 2020[2].

Des changements significatifs sont survenus au cours des deux dernières décennies concernant la prise en charge de la MAS, passant d'un traitement hospitalier à un traitement ambulatoire grâce au développement des Aliments Thérapeutiques Prêts à l'Emploi (ATPE) et au protocole de Prise en charge Intégrée de la Malnutrition aigüe (PCIMA).
Les services de santé publique ont cherché à rendre les interventions clés pour la survie des enfants plus intégrées et plus accessibles. L'approche Prise en Charge intégrée des Maladies de l'enfant - Communautaires (PCIME-C) ou ICCM (Integrated Community Case Management) a été introduite afin d'améliorer le recours aux soins dans des zones où l'accessibilité aux structures sanitaire est difficile. Cette approche est basée sur la formation des agents de santé communautaire (ASC) afin de fournir des services de traitement spécifiques pour des maladies infectieuses entraînant une forte mortalité. La preuve de l'efficacité des ASC dans le traitement de la MAS a été synthétisée dans une revue récente publiée par Action Contre la Faim et ses partenaires[3].
Cependant, il y a très peu des données relatives au traitement de la MAS par les ASC dans des situations d'urgence.

Le Nord du Mali est classé comme « crise oubliée » et au rang de « Risque élevé » dans l'indice de gestion des risques INFORM 2019 (16ème sur 191 pays). Cela s'explique par la détérioration de la situation sécuritaire, la difficulté d'accès aux services sociaux de base et l'exposition aux dangers climatiques. La situation humanitaire complexe, exacerbée par l'insécurité, la crise politique et les conflits intercommunautaires, contribue considérablement à la vulnérabilité de la population. Le Cadre Harmonisé de novembre 2020 estime à 46 171 le nombre de personnes actuellement en situation d'insécurité alimentaire et le projette à 105 509 durant la période de soudure (juin – août 2021). Dans le cercle de Gao, 4 des 7 communes sont projetées en Difficultés Economiques Sévères et les 3 autres en Difficultés Economiques Légères, la région de Gao étant la 2ème plus à risque dans Mali après celle de Mopti. Selon les résultats de la SMART de décembre 2020 les enfants de 6 à 59 mois dans la

[2] UNICEF-WHO-The World Bank : Joint child malnutrition estimates – levels and trends – 2021 edition [Internet]. UNICEF DATA. 2021 [cité 6 mai 2021]. Disponible sur : https://data.unicef.org/resources/jme-report-2021/

[3] López-Ejeda et al. (2018). Can Community Health Workers manage uncomplicated severe acute malnutrition? A review of operational experiences in delivering SAM treatment through community health platforms. Maternal and Child Nutrition, in press. DOI: 130.1111/mcn.12719

région de Gao connaissent une prévalence importante de la malnutrition avec une prévalence de la malnutrition aigüe globale (MAG) à 7,2 % [5.2- 9.9], la malnutrition aigüe sévère (MAS) à 0,9 [0.3- 2.3] et la malnutrition aigüe modérée (MAM) à 6,3 % [4,4- 9,0].[4]

Action Contre la Faim et ses partenaires ont élaboré un projet de recherche concernant le district sanitaire de Gao intitulé « Efficacité, coût-efficacité et couverture du traitement de la malnutrition aigüe sévère délivré par les agents de santé communautaire à travers le protocole modifié dans des contextes d'urgence au Mali » qui a été implémenté de juillet 2020 à Juin 2021.
Cette recherche proposée est basée sur l'expérience préalable d'Action contre la Faim (ACF) en partenariat avec le Ministère de la Santé et de développement social, l'Institut National Santé Publique (INSP) du Mali et la Fondation Innocent dans la région de Kayes au sud (zone plus stable).

Une évaluation réalisée en du 20 février au 19 mars 2020 (Baseline) de la couverture du programme nutritionnel dans les 3 bras du Projet a estimé la couverture unique au niveau de la zone contrôle pour la MAS à 20,5% [12,0%-28,9%] et la MAM à 9,8% [6,4%-13,2%], au niveau du bras 1 pour la MAS à 25% [13,7%-36,3%] et la MAM à 13,9% [8,2%-19,5%] et au niveau du bras 2 pour la MAS à 6,3% [1,4%-11,2%] et la MAM à 12,1% [6,8%-17,3%].

Tableau 1: Les critères de traitement dans les trois bras du projet ICCM+ sont inclus dans le tableau ci-dessous.

Paramètre	Bras contrôle	Bras 1	Bras 2
Type de malnutrition	MAS et MAM	MAS et MAM	MAS et MAM
Niveau pyramide sanitaire	Centre de santé communautaire	Centre de santé communautaire et site ASC	Centre de santé communautaire et site ASC
Produit utilisé	Plumpy nut pour les MAS et plumpy sup/farine enrichie pour les MAM	Plumpy nut pour les MAS et plumpy sup/farine enrichie pour les MAM	Plumpy nut pour les MAM et MAS
Type de protocole	Protocole standard	Protocole standard	Protocole simplifié
Administration de la posologie	En fonction du poids de l'enfant	En fonction du poids de l'enfant	Posologie fixe et ne tient pas compte du poids de l'enfant

[4] 1. Rapport_Final_SMART_2020_Mali_Decembre2020.pdf.

Critère d'admission et de sortie	Admission<br>PT<-3z score ou PB<115mm<br>Sortie<br>PT>-1,5 z score et PB>125 mm	Admission<br>PT<-3z score ou PB<115mm<br>Sortie<br>PT>-1,5 z score et PB>125 mm	Admission<br>PB<115mm<br>Sortie<br>PB>125 mm

Un Essai randomisé contrôlé par grappes (ERCg) a été créé selon une conception de non-infériorité pour comparer trois protocoles différents de traitement de la MAS (différents diagnostics et différents dosages d'ATPE), comme indiqué dans le tableau 1. La conception ERC a été retenue, celle-ci étant considérée comme une méthodologie « de référence » pour évaluer les interventions de soins médicaux. Une approche par grappe est appliquée étant donné l'impossibilité de randomisation individuelle.

II. OBJECTIFS

1. Objectif général

L'enquête de couverture détaillée dans le présent rapport a été réalisée en tant qu'enquête End line de couverture pour l'étude de recherche, le principal objectif étant d'évaluer la couverture finale du traitement de MAS et MAM chez les enfants âgés de 6 à 59 mois dans les bras d'intervention (bras 1 et bras 2) et le bras contrôle de l'étude dans le district sanitaire de Gao.

2. Objectifs spécifiques

Les objectifs spécifiques de l'enquête étaient :

- Estimer la couverture unique du traitement de MAM et MAS dans le bras contrôle du projet après un an d'implémentation du projet ;
- Estimer la couverture unique du traitement de MAM et MAS dans le bras 1 après un an d'implémentation du projet ;
- Estimer la couverture unique du traitement de MAM et MAS dans le bras 2 après un an d'implémentation du projet ;
- Dans chaque bras de l'étude, analyser les facteurs négatifs (barrières) et facteurs positifs (boosters) basés sur les réponses fournies par les accompagnantes des enfants MAS et MAM identifiés pendant l'enquête

III. METHODOLOGIE

Dans cette partie relative à la méthodologie de l'enquête, nous présenterons dans un premier temps la procédure utilisée pour le calcul des tailles d'échantillon et l'échantillonnage des villages, puis nous aborderons les aspects relatifs à la collecte des données sur le terrain, incluant le questionnaire, les équipements, le personnel et l'organisation pratique, avant de terminer par le traitement et l'analyse des données.

1. Domaine couvert par l'enquête

II s'agit d'une enquête transversale sur un échantillon représentatif des ménages dans le bras contrôle, bras 1 et bras 2.

2. Echantillonnage et plan de l'enquête

2.1. Population d'étude

L'enquête a concerné les ménages présents au moment de l'enquête et vivant dans le bras contrôle, bras 1 et bras 2. Dans les maisons, tous les enfants âgés de 6 à 59 mois ont été inclus dans l'enquête.

2.2. Base de sondage

La base de sondage utilisée pour l'échantillonnage était constituée à partir des listes de villages par aire de santé du bras contrôle, bras 1 et bras 2. Les chiffres de population sont ceux fournis par la région médicale pour le district sanitaire de Gao (population actualisée pour l'année 2021).

2.3. Taille de l'échantillon

Dans le cadre des enquêtes de couverture pour les programmes PCIMA, tant que les enfants qui souffrent de la malnutrition aigüe sont assez rares dans la communauté (surtout ceux qui souffrent de la MAS), les estimations de couverture de traitement peuvent être calculées basées sur les petites tailles d'échantillon avec une précision acceptable. Si le nombre d'enfant MAS tombe en-dessous de 500 dans une zone d'intervention, il est possible de réduire la taille d'échantillon[5].

Afin de réaliser les objectifs de cette enquête, l'équipe Action Contre la Faim a mené 3 enquêtes distinctes à grande échelle portant sur les indicateurs précis. Trois tailles d'échantillons des cas MAS et trois tailles d'échantillon des cas MAM ont été calculées (une taille d'échantillon pour les MAS et une pour les MAM dans chaque bras de l'étude). Les résultats de chaque enquête ont ensuite été analysés pour estimer la couverture du traitement MAS et MAM avec un intervalle de confiance de 95%.

Les tailles d'échantillon ont été calculées en utilisant la calculatrice Sampsize[6]. Pour ce faire, les données suivantes ont été ajoutées à la calculatrice. L'échantillonnage était basé sur la population des cas MAS dans les zones de l'étude en considérant que la MAS est la maladie le plus rare en comparaison avec la MAM :

Précision :

La précision souhaitée de l'estimation finale. Une précision de 10 à 15% est acceptable pour les estimations de la couverture. Dans le cadre de cette enquête, une précision de 12% a été utilisée pour l'estimation de la couverture de traitement de la MAS.

Prévalence :

La couverture de traitement estimée de 30% a été utilisée sur la base des estimations de la couverture de base pour cette enquête End line.

Niveau :

Le niveau souhaité de l'intervalle de confiance 95% est utilisé pour les enquêtes de couverture.

Population des enfants MAS :

L'estimation du nombre d'enfants souffrant de la MAS dans les zones d'enquête a été calculée selon les détails dans le tableau 3.

[5] Plus d'informations à la page 127 du manuel technique SQUEAC et SLEAC(36)

[666] http://sampsize.sourceforge.net/iface/index.html#prev

Les populations attendues pour la malnutrition aigüe ont été calculées sur la base des estimations de prévalence les plus récentes et les plus précises, ainsi que sur les populations d'enfants âgés de 6 à 59 mois. La formule suivante a été utilisée (n = population estimée de cas MAS dans chaque unité de prestation de services) :

n=

$$\left| pop.moyenne\ par\ village_{tous\ les\ ages} \times \frac{\%\ de\ la\ population_{6-59\ mois}}{100} \times \frac{prévalence\ MAS}{100} \right|$$ Prévalence

de la MAS :
La prévalence MAS, basée sur les estimations PT tirées de l'enquête SMART de décembre 2020, a été utilisé car la taille de l'échantillon serait acceptable pour permettre une mise en œuvre réaliste de l'enquête (Tableau 2).

Tableau 2: Prévalence de la malnutrition aigüe sévère à Gao, enquête SMART, Décembre 2020

Régions/ Départements	Z−score <−2 et/ou œdèmes (IC_{95})	<-2 Z-score et >=-3 Z-score pas d'œdèmes (IC_{95})	Z−score <−3 et/ou œdèmes (IC_{95})
Gao	7,2 [5,2- 9,9]	6,3 [4,4- 9,0]	0,9 [0,3- 2,3]

Cependant, vue que l'enquête SMART 2020 a été effectuée en décembre, et que l'enquête de couverture a été réalisée en mai-juin (période de soudure), on s'attendra à avoir des prévalences de la malnutrition plus élevées. L'enquête de couverture Baseline de 2020 a été réalisée en février-mars 2020. Basé sur les résultats de 2020, la prévalence MAS « proxie » a été environ de 3,6%. Par la suite, une prévalence MAS de 2% a été utilisée pour l'enquête de 2021 (pour être sûr que les tailles d'échantillon seront atteintes).

Population moyenne par village tous âges :
En utilisant les données de population par village et les tailles d'échantillon calculées pour chaque bras de l'étude, il est possible de calculer le nombre de villages à visiter pour atteindre les tailles d'échantillon des cas MAS (Tableau 3).

Tableau 3: Calculs de la taille d'échantillon pour les cas MAS pour chaque bras de l'étude

Paramètres	Contrôle	Bras 1	Bras 2	Totale
Population totale	89 855	44 942	53 427	188 224
Nombre de villages	62	28	60	150
Population moyenne par village	1 449	1605	890	
% d'enfants de 6 à 59m	18%	18%	18%	
Population d'enfants de 6 à 59m	16 174	8 090	9 617	33 880
Prévalence MAS par poids / taille	2%	2%	2%	
Nombre d'enfants qui sont MAS	323	162	192	678

Taille d'échantillon des cas MAS	48	42	44	48
Nombre de villages à visiter	23	20	21	64

2.4. Sélection des grappes

➢ Calcul du nombre de villages à visiter

Sur la base des tailles d'échantillons requises, l'étape suivante consistait à calculer le nombre requis de villages ($n_{village}$) à visiter pour atteindre les tailles d'échantillons

$$n_{villages} = \left\lceil \frac{n}{population\ moyenne\ par\ village_{tous\ les\ âges} \times \frac{pourcentage\ de\ la\ population_{6-59\ mois}}{100} \times \frac{prévalence\ MAS}{100}} \right\rceil$$

requises des cas MAS (le nombre de villages calculé est disponible dans le Tableau 3).

➢ Sélection des villages

Pour la sélection des grappes, nous avons considéré les villages/quartier comme la plus petite unité administrative pouvant abriter les grappes. La sélection aléatoire des villages a été faite au moyen de la méthode d'échantillonnage aléatoire stratifié systématique pour assurer une représentativité spatiale en utilisant une liste de villages organisée par CSCOM et site ASC. Cette méthode de tirage a été utilisée dans la mesure qu'il n'y a aucune carte détaillée/complète disponible. La liste des villages a été organisée par ordre alphabétique par aire de santé et par village et ils ont été numérotées. Un pas d´échantillonnage a été calculé et appliqué pour la sélection des villages respectivement pour la zone d'intervention (bras 1 et 2) et contrôle dans les CSCOM. Le pas de sondage peut être calculé avec la formule suivante :

$$Pas\ de\ sondage = \frac{Nombre\ total\ de\ villages\ et\ ou\ communs}{Nombre\ de\ villages\ à\ visiter}$$

Pour choisir le premier village, nous avons sélectionné un numéro aléatoire entre 1 et le pas de sondage en utilisant Excel. Ensuite elles sont passées au village suivant en fonction du pas de sondage. Par la suite, on a ajouté ainsi le pas de sondage jusqu'à la fin des listes des villages. Enfin, les villages ont été planifiés par les enquêteurs et superviseurs pour la recherche des cas dans les villages.

2.5. Sélection des ménages

Seuls les ménages ordinaires ont été concernés pour cette enquête. Autrement dit, pour cette enquête, ont été exclus les couvents, les orphelinats, les hommes, des vieillards, les prisons et les permanences des mosquées. Une fois arrivée dans le village/quartier d'enquête, les enquêteurs cherchaient à enquêter tous les ménages dans le village/quartier.

Les équipes ont procédé à un échantillonnage porte à porte afin d'identifier les « cas ». L'échantillonnage porte à porte est la méthode d'échantillonnage recommandée à utiliser pour tenter d'identifier tous les cas de MAM dans les villages.

2.6. Sélection des sujets

- Définition de cas :

Dans le cadre de cette enquête, un cas inclut tout enfant de 6 à 59 mois de la population MAS ou MAM au moment de l'enquête et / ou tout enfant inscrit dans une structure sanitaire de traitement de la MAS ou MAM au moment de l'enquête.

- Méthode d'identification des cibles :

Une fois les villages sélectionnés, les équipes d'enquête se sont rendues dans chaque village pour dépistage exhaustif dans la communauté de tous les cas éligibles afin d'enregistrer leurs données anthropométriques et déterminer s'ils sont inscrits ou non au programme de traitement concerné.

Dans chaque ménage, tous les enfants âgés de 6 à 59 mois, s'y trouvant était tous inclus pour les mesures anthropométriques (mesure de poids, taille, œdèmes et périmètre brachial).

Si les occupants d'une maison n'étaient pas présents, les enquêteurs revenaient visiter la maison avant la fin de la journée.

2.7. Procédure d'échantillonnage pour la recherche des raisons pour les cas couverts et non couverts

Les données qualitatives collectées auprès des accompagnants des cas non couverts ou non couverts identifiés ont été analysées pour identifier et classer les raisons de la non-participation au traitement (obstacles). Cela a été fait en analysant les résultats dans la base de données « ODK Collect» de l'enquête.

3. Préparation de la collecte des données

3.1. Données anthropométriques

- Vérification de l'âge :

Pour déterminer l'âge des enfants identifiés, les équipes de l'enquête ont d'abord demandé si l'accompagnant peut leur montrer un acte de naissance et/ou une carte de santé ou carnet de vaccination. Si l'accompagnant ne pouvait fournir aucun de ceux-ci et ne pouvait pas donner d'âge exact pour l'enfant, l'équipe d'enquête a utilisé un calendrier des événements clés locaux pour déterminer l'âge de l'enfant.

- Identification des cas MAS et MAM :

Les cas MAS et MAM ont été identifiés par PB, présence d'œdème et / ou par le Z score du poids pour la taille. Lors d'une évaluation de la couverture, les cas sont généralement identifiés par les équipes de collecte de données sur la base du protocole d'identification

et de référence des cas de malnutrition au Mali. Cependant, pour cette enquête de couverture nous avons décidé de calculer les Z scores de tous les enfants dont le PB était

inférieur à 140 mm. Ce seuil a été déterminé à base de l'analyse des données mensuelles des enfants pris en charge dans le programme qui montrent que les cas MAS et MAM ont été identifiés avec des mesures de PB supérieures à 125 mm mais ces enfants ont été classés comme MAS ou MAM par Z-score. Le z-score a été calculé automatiquement par le logiciel « NutriSurvey.ena delta » version de janvier 2020.

Les définitions de cas pour l'identification des cas MAS et MAM lors de l'évaluation de couverture sont résumées dans le tableau 4 ci-après.

Tableau 4: Définitions des cas MAS et MAM dans les zones d'intervention et contrôle, Mai 2021

Description	Périmètre brachial	Œdème	Z-score
Malnutrition aigüe sévère	<115 mm	+, ++, +++	<-3
Malnutrition aigüe modéré	115-124mm	Pas d'œdème	-2 et -3

- Confirmation de l'inscription dans un programme :

Les enfants inscrits dans une structure sanitaire pour prise en charge de la MAM ou MAS devraient remplir les critères ci-dessous.

Tableau 5: Définitions des cas MAS et MAM dans les zones d'intervention et contrôle, Mai 2021

Traitement MAS	Traitement MAM
Possède les plumpy nut Ou Confirmation par un relais communautaire	Carte d'identification Ou Plumpy sup ou farine enrichie Ou Confirmation par le relais communautaire
S'il n'était pas possible de confirmer que l'enfant participait au programme concerné, alors il était considéré comme un cas non couvert.	

- Classification des cas identifiés pendant l'enquête

Tableau 6: Définitions des cas couverts et non couverts dans les zones d'intervention et contrôle, Mai 2021

Cas	Définitions
MAS C	Enfant est MAS et reçois actuellement traitement pour MAS
MAS NC	Enfant est MAS et ne reçois pas traitement pour MAS
MAS EVDG	Enfant n'est plus MAS mais reçois encore traitement pour MAS
MAM C	Enfant est MAM et reçois actuellement traitement pour MAM
MAM NC	Enfant est MAM et ne reçois pas traitement pour MAM
MAM EVDG	Enfant n'est plus MAM mais reçois traitement pour MAM

3.2 Estimation de la couverture avec intervalle de confiance à 95%

Une fois l'enquête terminée dans les villages sélectionnés, les équipes d'enquête ont communiqué les totaux de chaque classification de cas à l'équipe de coordination de l'enquête. Lorsque tous villages sélectionnés ont été visités par les équipes et les tailles des échantillons étaient atteintes ou dépassées, il était alors possible d'estimer la couverture. Pour la couverture de MAM et MAS, l'estimateur de couverture unique[7] est l'estimateur recommandé. En effet, l'estimateur de la couverture unique est un estimateur qui remplace les deux estimateurs précédents : couverture actuelle et période. L'estimateur unique est un estimateur de couverture développé depuis 2015 qui est conseillé pour tous les contextes

Pour la crédibilité et sa validité statistique, la couverture unique doit être calculée en ajoutant un nouvel élément : les cas en voie de guérison hors du programme, ou cas en voie de guérison spontanée. Cet élément ne peut pas être calculé lors des enquêtes. En revanche, une équation a été développée pour estimer cette valeur en fonction des données disponibles des cas MAS/MAM couverts, cas MAS/MAM non couverts et cas en voie de guérison dans le programme. Dans le cadre de la présente investigation. Le calcul de la couverture unique utilise les données de l'enquête selon la formule suivante :

[7] Pour plus d'information: Myatt, M et al, (2015) *A single coverage estimator for use in SQUEAC, SLEAC, and other CMAM coverage assessments*, p.81 Field Exchange 49.

$$\text{Couverture unique} = \frac{C_{in} + R_{in}}{C_{in} + R_{in} + C_{out} + R_{out}}$$

C_{in} : Nombre de cas MAS couvert dans le programme
C_{out} : Nombre de cas MAS non couvert hors
programme R_{in} : Nombre en voie de guérison dans le
programme R_{out} : Nombre en voie de guérison hors du
programme

$$R_{out} \approx \frac{1}{k} \times \left(R_{in} \times \frac{C_{in} + C_{out} + 1}{C_{in} + 1} - R_{in}\right)$$

Ou le facteur de correction «K» est toujours 3. Ceci à cause de la relation entre la durée moyenne d'un épisode MAS guéri dans le programme (2,5 mois) et la durée moyenne d'une guérison spontanée (7,5 mois).

- Calcul de l'intervalle de confiance à 95%

L'intervalle de confiance de 95% pour chacune des estimations de couverture a été calculé à l'aide de la formule suivante :

$$95\%\ CI = Coverage\ \pm 1.96 \times \sqrt{\sum \frac{\frac{c}{n} \times (1 - \frac{c}{n})}{n}}$$

CI = intervalle de confiance
c = numérateur
n = dénominateur

- Données sur les barrières et boosters

Le questionnaire pour les cas couverts et non couverts a été administré à tous les cas identifies. Ces questionnaires ont permis de déterminer les raisons principales de non-fréquentation (pour les cas non couverts) et les principales raisons de participation au programme (pour les cas couverts).

Le questionnaire pour enfants non couverts a pour but de permettre à l'enquêteur de déterminer les raisons principales de non-participation au programme. Le questionnaire

suivait la logique suivante :
La première question a été posée aux accompagnants en relation avec le dépistage et la périodicité. Par la suite la question suivante a été posée : pensez-vous que votre enfant

est malade ? Si la réponse à cette question était « Non », alors l'intervieweur a mis fin au questionnaire. C'est parce que si l'accompagnant n'était pas conscient que leur enfant fût malade, puis posant davantage de questions sur les symptômes et la maladie de l'enfant n'apportait pas une valeur ajoutée. Si l'accompagnant savait que leur enfant était malade, l'intervieweur a ensuite posé la question suivante :

- Questions : « De quels symptômes souffre votre enfant ?» ; « Quelle maladie a causé ces symptômes ?» ; "Comment avez-vous essayé de traiter cette maladie ou comment allez-vous la traiter ?" Et ; "Qui a pris une décision concernant le choix du traitement ? ". Pour chacune de ces questions, les accompagnants pourraient fournir des réponses multiples.
- On a ensuite demandé aux accompagnants : Savez-vous qu'il existe un service dans l'établissement de santé dédié au traitement de la malnutrition ? S'ils ont répondu non à cette question, l'intervieweur a mis fin au questionnaire.
- Si les accompagnants savaient que leur enfant était malade et qu'ils connaissaient l'existence d'un programme où ils pouvaient recevoir un traitement contre la malnutrition, on leur a ensuite demandé : pourquoi n'avez-vous pas amené votre enfant au centre de santé pour le traitement ? Les intervieweurs devaient choisir une raison parmi une liste de raisons communément citées, ou ils pourraient sélectionner « Autre » et préciser la raison si la raison fournie n'était pas sur la liste.
- On a également demandé aux personnes qui connaissaient l'existence du programme si leur enfant avait été précédemment inscrits au programme et comment ils avaient été libérés (en tant que cas guéri, non-répondant).

L'objectif du questionnaire pour les cas couverts était d'identifier ce qui avait influencé l'accompagnant d'aller au poste de santé pour chercher un traitement pour leur enfant malnutri.

- La première question concernait l'accompagnant par rapport au dépistage et sa périodicité. Par la suite, on n'a tenu compte de la précédente inscription de leur enfant dans le programme de traitement. Si l'enfant avait rechuté ou avait fait défaut, l'interviewer demanderait de suivre des questions pour essayer de comprendre pourquoi.
- La deuxième question demandait si l'accompagnant avait d'autres enfants inscrits dans les programmes de nutrition
- La troisième question était la suivante : pourquoi avez-vous décidé d'inscrire votre enfant au programme de nutrition ? Les intervieweurs devaient choisir une raison parmi une liste de raisons communément citées, ou ils pourraient sélectionnez « Autre » et spécifiez le motif ;

3.3. Ressources Humaines de l'enquête

Cette enquête a été préparée par l'équipe technique de Nutrition d'ACF du Mali avec l'appui du bureau régional Afrique de l'Ouest et du Centre d'ACF et d'ACF UK. Pour la phase de collecte des données sur le terrain, 6 équipes seront formées. Chaque équipe

était composée d'un superviseur et de deux enquêteurs (SLEAC), soit au total 6 superviseurs d'équipe, 12 enquêteurs et un superviseur général. Les équipes ont été supervisées en continue avec une rotation permettant une vision d'ensemble des équipes. Elles ont été appuyées sur le terrain par le Chef de projet iCCM et le Coordinateur régional iCCM.

3.4. Formation des enquêteurs

La formation des enquêteurs a été réalisée en 3 jours (2 jours en salle et 1 jour pré-enquête) et à regrouper tous les 12 enquêteurs, les 6 superviseurs, l'équipe de la supervision générale (MEAL, le Chef de projet iCCM et le Coordinateur régional iCCM). Elle a comporté des séances en salle où ont été abordées les questions relatives à l'attitude générale et au comportement des enquêteurs, aux principes de remplissage du questionnaire, à la compréhension du questionnaire ainsi qu'à la traduction du questionnaire en langues locale. Cette formation en salle a également comporté la présentation des outils de mesures anthropométriques et la démonstration de leur utilisation.

Avant le démarrage de l'enquête proprement dite, une pré-enquête a été effectuée dans des conditions réelles. Ceci a donné l'occasion aux enquêteurs et aux superviseurs de travailler dans le cadre de leurs équipes respectives, pour mettre en pratique toutes les étapes depuis l'introduction dans le village, l'échantillonnage, les interviews et la prise des mesures anthropométriques.

Cette pré enquête a permis de :

- Tester et se familiariser aux questionnaires.
- Adopter une méthode de travail sur le terrain
- Adapter la logistique aux impératifs de déplacements fréquents.

3.5. Standardisation des mesures

Dans le cadre de la préparation des enquêteurs aux mesures anthropométriques, des enfants de 6 à 59 mois ont été identifiés pour participer à l'opération. Les superviseurs ont servi de référence pour les mesures de poids, taille et œdèmes.

Deux tests de standardisation des mesures anthropométriques ont été organisés, parallèlement, suivant les recommandations de la méthodologie SMART. Les agents travaillaient en binômes pour mesurer chacun deux fois (poids, taille, PB) 6 enfants de moins de cinq ans, à tour de rôle.

Les mesures ont été saisies et analysées sous le logiciel ENA, et les résultats obtenus ont permis d'évaluer la précision (écart observé entre deux mesures d'un même mesureur) et l'exactitude des mesures (écart observé entre la mesure de l'enquêteur et celle du formateur) pour chaque enquêteur, et de sélectionner les 12 meilleurs mesureurs pour l'enquête. Quant à la taille, la précision a été jugée acceptable, cela était lié beaucoup plus à la fatigue et l'inattention, mais pas à une méconnaissance de la méthode.

3.6. Traitement et analyse des données

- Vérification et nettoyage des fiches sur le terrain

Les fiches de collecte de données ont été quotidiennement vérifiées par chaque superviseur avant d'être reçues par le coordonnateur. A ce niveau, la base de données dans ODK est téléchargée afin d'analyser les données et si possible demander à chaque superviseur des informations supplémentaires. Nous avons identifié des données manquantes qui ont pu être complétées. Dans l'ensemble, tous ces problèmes ont été considérablement réduits après les 3 premiers jours de terrain.

- Saisi et analyse statistique des données

Toutes les données ont été analysées à partir des logiciels ENA-delta version janvier 2020 et Excel. Les indices nutritionnels ont été calculés en utilisant la population de référence OMS (2006).

La première saisie des questionnaires enfants (6-59 mois) s'est réalisée au cours de la phase de collecte des données, sur le terrain, par les superviseurs, sur le logiciel ENA-delta version janvier 2020 pour les données anthropométriques des enfants. Les données anthropométriques ont été saisies sur le lieu de collecte de la grappe du jour, avant de quitter.

La saisie dans la grappe du jour permettait de voir les erreurs/flags, de les corriger avant de quitter la grappe, et de donner un retour aux mergERCesureurs sur la qualité de leurs mesures et de l'évaluation de l'âge. Chaque superviseur sauvegardait par ailleurs (et en plus de l'ordinateur), sur une clef USB, les fichiers de la saisie du jour.

La qualité de la collecte de données du jour était analysée par le superviseur accompagné de la coordination (rapport de plausibilité), afin de faire un retour le soir même aux équipes. Les superviseurs sauvegardaient, chaque jour, tous les fichiers (ENA) de leur équipe et transmettaient les données quotidiennement à la coordination. Une double saisie s'est effectuée au retour de la collecte des données.

4. Organisation sur le Terrain

Les superviseurs ont assisté à toutes les activités de préparation et de planification de l'étude assurant un travail d'équipe.

Avec un manque de cartes assez détaillées dans toutes les aires de santé, les équipes ont été responsabilisées pour discuter avec les personnes clés/informées pour clarifier les axes de travail pour visiter les villages échantillonnés. Ainsi la planification détaillée des équipes était sous la responsabilité des superviseurs.

Un manuel de terrain a été préparé et distribué aux superviseurs et enquêteurs pour clarifier les procédures sur le terrain. Cela a permis dans une certaine mesure, de s'assurer que la méthodologie a été suivie d'une manière uniforme malgré la distance du superviseur et/ou le manque de réseaux téléphonique. Toutes autres questions et clarifications ont été discutées avec l'équipe d'enquête et les superviseurs tout au long de l'enquête pour diriger la collecte de données de qualité.

Pendant l’enquête, les activités ont été supervisées par l’équipe d’enquête avec les superviseurs en s’assurant sur le terrain que chaque équipe ait été visitée lors de la collecte

de données quantitatives. Un formulaire de supervision a été développé pour assister les superviseurs dans l'identification des points à améliorer.

5. Limites de l'enquête

➢ Imprécision dans l'âge des enfants :

La majorité des enfants n'ont pas de document officiel précisant leur date de naissance. Les mamans ou membres des familles n'ont qu'une connaissance très approximative de l'âge des enfants. De ce fait, malgré les efforts des équipes et l'utilisation systématique du calendrier des évènements, la détermination de l'âge des enfants exigeait des profondes investigations.

➢ Limite de l'évaluation

Les données anthropométriques sont les seules informations quantitatives donc non subjectives pour apprécier la situation nutritionnelle des populations. Pour les données quantitatives obtenues par interview sur l'état de santé, leur utilisation et interprétation ne peuvent se faire qu'à titre indicatif.

➢ Imprécision dans la base des données démographiques :

Dans certains villages identifiés, il arrivait parfois que les données démographiques mises à disposition par la région médicale soient inférieures à la réalité.

6. Problèmes rencontrés

La réussite de l'enquête est due certainement à la double participation des enquêteurs qui ont perçu l'enjeu de cette étude et des populations qui ont bien compris ses objectifs. Les problèmes rencontrés mais sans incidence sur les résultats sont :

- L'accessibilité géographique difficile pour certains villages avec le début de la période hivernale.
- L'insécurité dans le district sanitaire de Gao

7. Considérations éthiques

L'évaluation a été réalisée en respectant les principes éthiques suivants :

- Respect de l'anonymat et de la confidentialité ;
- Principe de non-jugement ;
- Libre expression des personnes ;
- Fidélité des témoignages et opinions exprimés.

Les enfants de moins de 5 ans éligibles à une prise en charge de la malnutrition aigüe ont été systématiquement orientés et référés vers les centres de prise en charge les plus proches en cas de dépistage positif de la malnutrition aigüe modérée ou sévère.

Les autorités de chaque district ont été contactées et dûment informées de la réalisation de l'évaluation dans leur zone. Les équipes de terrain ont eu aussi la responsabilité d'informer les responsables de chaque localité (Chef de village, collectivité, DTC) avant

leur arrivée et solliciter leur appui, selon le besoin.

IV. RESULTAT

1. Description de l'échantillon

Tableau 7: Synthèse des données collectées pendant l'enquête a grande échelle dans Gao, mai 2021

	Contrôle	Bras 1	Bras 2	Total
N° Villages visités	23	21	21	65
N° Enfants dépistés	2448	2412	1759	6619
Cas MAM	255	329	111	696
Cas MAS	102	120	36	258
Total MAM+MAS	358	449	147	954

Le tableau 7 montre que tous les villages sélectionnés ont été visités par les équipes. Un total de 6619 enfants ont été mesurés par le PB par les équipes. Parmi ces enfants, ce sont 1 056 enfants dont les poids et tailles ont été mesurés afin de calculer le z-score des enfants. Tous les enfants avec un PB de moins de 140 mm ont été mesurés pour le rapport P/T. Dans les villages échantillonnés 358, 449, 147 cas de MAS et MAM ont été trouvés respectivement pour le bras contrôle, bras 1 et bras 2, ce qui représente environ 37,52%, 47,06% et 14,40% de tous les enfants. Le bras 1 a eu la taille d'échantillon (MAM+MAS) des cas identifiés la plus élevée.

Tableau 8: Comparaison taille de l'échantillon attendue et atteinte dans les zones d'intervention et contrôle, mai 2021

	Contrôle		Bras 1		Bras 2	
	MAS	MAM	MAS	MAM	MAS	MAM
Attendue	48	96	42	96	44	96
Atteinte	105	258	150	318	40	110
	218%	268%	357%	331%	91%	114%

Pour pouvoir estimer la couverture de la MAM et MAS, il était nécessaire que les équipes d'enquête identifient les tailles d'échantillon cibles de chacune d'elles. Les tailles d'échantillon ont été calculées en fonction de la population attendue d'enfants MAS de 6 à 59 mois concernée dans chacun des bras de l'étude.
Le tableau 8 montre les cas constatés à la fin de l'enquête dans les 65 villages visitées. Les tailles d'échantillon pour les cas MAS ont été atteintes dans le bras Contrôle et dans le Bras 1. Dans le Bras 2, 40 des 44 cas de MAS ont été identifiés. Cette taille d'échantillon est suffisante d'estimer la couverture avec une précision acceptable.

Tableau 9: Cas MAS identifiés selon les critères d'admission

	Od	Od+PB	Od+PT	PB	PB+PT	PT	Total
Contrôle	0	1	1	25	33	42	102
Bras 1	2	0	0	21	27	70	120,
Bras 2	0	0	0	3	10	23	36
Total	2	1	1	49	70	135	258
	0.8%	0.4%	0.4%	19.0%	27.1%	52.3%	

Tableau 10: Cas MAM identifiés selon les critères d'admission

	PB	PB+PT	PT	Total
Contrôle	61	83	111	255
Bras 1	76	80	173	329
Bras 2	23	48	40	111
Total	160	211	324	695
	23%	30%	47%	

Le tableau 9 et 10 nous montrent que les cas de MAS et MAM identifiés selon les critères d'admission. Ainsi, 52,3% des cas de MAS ont été identifiés uniquement par le critère P/T. Parmi les cas MAM identifiés, 47% ont été MAM uniquement par le rapport P/T. Ces résultats reflètent les résultats de l'enquête SMART la plus récente réalisée en décembre 2020. Dans le bras « Contrôle », la proportion des cas qui ont été MAM et MAS par le PB et le PB+PT a été plus élevée que la proportion des cas qui ont été MAM et MAS uniquement par le rapport PT. Ce qui indique que la recherche des cas actifs dans la communauté est plus faible dans le bras Contrôle. Ces informations ont été triangulées par les données du Figure 1.

2. Estimation de la couverture

Tableau 11: Estimations de couverture du programme de traitement MAS dans les trois bras de l'étude

	Cas trouvés				Dénominateur	Numérateur	Estimation de couverture	Intervalles de confiance	
	Cin	Cout	Rin	Rout	Cin+Cout + Rin+Rout	Cin+Rin	Numerator/ Denominator	Lower CI	Upper CI
Contrôle	17	84	4	6	111	21	18.9%	11.6%	26.2%
Bras 1	38	85	27	19	169	65	38.5%	31.1%	45.8%

Bras 2	17	20	3	1	41	20	48.8%	33.5%	64.1%

Les estimations de couverture du programme MAS indiquent une augmentation de la couverture dans le Bras 1 et le Bras 2 comparativement à l'enquête de mars 2020. La couverture du programme MAS dans le Bras 2 a augmenté significativement (aucun chevauchement des intervalles de confiance entre 2020 et 2021). Dans le Bras 1, la couverture a augmenté mais pas d'une manière significative. Il y a un petit chevauchement des intervalles de confiance entre 2020 et 2021. La couverture de traitement dans le bras « Contrôle » n'a pas évolué pendant la mise en place l'étude. Toutes les estimations tombent au-dessous du standard Sphère dans les zones rurales (50%). Mais les couvertures dans Bras 1 et 2 peuvent être classifie comme « Modérée » (entre 20 et 50%).

Tableau 12: Estimations de couverture du programme de traitement MAM dans les trois bras de l'étude

	Cas trouvés				Dénominateur	Numérateur	Estimation de couverture	Intervalles de confiance	
	Cin	Cout	Rin	Rout	Cin+Cout + Rin+Rout	Cin+Rin	Numerator/ Denominator	Lower CI	Upper CI
Contrôle	38	215	5	9	267	43	16.1%	11.7%	20.5%
Bras 1	58	236	24	32	350	82	23.4%	19%	27.9%
Bras 2	24	80	6	6	116	30	25.9%	17.9%	33.8%

Les estimations de couverture du programme MAM également indiquent une augmentation de la couverture dans le Bras 1 et le Bras 2 comparativement à l'enquête de mars 2020 mais aucun changement dans le bras Contrôle. Les estimations de couverture pour les Bras 1 et 2 se trouvent dans la classification « modérée » parce qu'elles dépassent les 20%. Mais même dans ces bras de l'étude, seulement un quart des enfant MAM ont été couvert par le traitement.

Tableau 13: Classification de traitement MAM et MAS par aire de santé dans le bras Contrôle (classification selon la couverture de période)

Contrôle	Classification MAM	Classification MAS
Aljanabandja	≤20%	20 a 50%
Dioulabougou	≤20%	≤20%
Gadeye	≤20%	≤20%
Kochakareye	>20 a 50%	>50%
Tacharane	≤20%	≤20%
Tin Aouker	≤20%	≤20%

Tableau 14: Classification de traitement MAM et MAS par aire de santé dans le Bras 1 (classification selon la couverture de période)

Bras 1	Classification MAM	Classification MAS
Forgho	>20 a 50%	>20 a 50%
Lobou	>20 a 50%	>20 a 50%
Zinda	>20 a 50%	>20 a 50%

Tableau 15: Classification de traitement MAM et MAS par aire de santé dans le Bras 2 (classification selon la couverture de période)

Bras 2	Classification MAM	Classification MAS
Bagnandji	≤20%	>20 a 50%
Magnadoue	>20 a 50%	>50%
Wabaria	>20 a 50%	>50%

Les tableaux 13, 14 et 15 nous indiquent la classification de traitement MAM et MAS par aire de santé dans les 3 bras d'étude (classification selon la couverture de période). Avec ses résultats, il a été possible de classifier la couverture de traitement MAS et MAM comme « basse », « modérée » et « haute » basée sur les cas trouvés.
Dans le bras « Contrôle », dans la plupart des aires de santé les couvertures ont été « basses » pour le traitement MAS et MAM. Mais dans l'aire de santé de Kochakareye la couverture a été modérée pour le traitement de la MAM et élevée pour le traitement de la MAS. Dans le Bras 1, la couverture dans toutes les aires de sante a été modérée. Dans le Bras 2, on peut constater des classifications « élevées » pour le traitement de la MAS au-dessus de 50% dans deux aires de santé. Mais dans l'aire de santé de Bagnadji, les résultats ont indiqué une couverture moins élevée. Globalement, la couverture a été la plus élevée dans le Bras 2 pour le traitement de la MAM et de la MAS.

- Estimations de couverture selon les critères d'admission

Basé sur les données collectées par les questionnaires Kobo, il est possible de désagréger les résultats par deux principaux critères d'admission :

- Critère 1 : Par PB uniquement ou par PB+P/T
- Critère 2 : Par P/T uniquement

Si le nombre de cas trouvé tombe très bas, il n'est pas possible d'estimer la couverture. Cependant on peut classifier la couverture sur une échelle à trois niveaux. Les résultats sont estimés en utilisant la *couverture actuelle* au lieu de la *couverture unique*.
Les résultats pour les deux critères (pour les cas MAS et MAM) sont résumés dans les Tableaux ci-dessous.

Tableau 16: Estimations de couverture pour les cas MAS selon le Critère 1 (Par PB uniquement ou par PB+P/T)

	Cas trouvés		Dénominateur	Numérateur	Estimation de couverture	Intervalles de confiance	
	Cin	Cout	Cin+Cout	Cin	Numerator/ Denominator	Lower CI	Upper CI
Contrôle	10	48	58	10	17.2%	7.5%	27.0%
Bras 1	20	28	48	20	41.7%	27.7%	55.6%
Bras 2	9	6	15	9	Elevée		

Tableau 17: Estimations de couverture pour les cas MAS selon le Critère 2 (Par P/T uniquement)

	Cas trouvés		Dénominateur	Numérateur	Estimation de couverture	Intervalles de confiance	
	Cin	Cout	Cin+Cout	Cin	Numerator/ Denominator	Lower CI	Upper CI
Contrôle	6	35	41	6	14.6%	3.8%	25.5%
Bras 1	18	56	74	18	24.3%	14.5%	34.1%
Bras 2	8	14	22	8	Modérée		

Les tableaux 16 et 17 indiquent les estimations de couverture pour les cas MAS selon le Critère 1 (Par PB uniquement ou par PB+P/T) et le critère 2 (Par P/T uniquement).
Pour les cas MAS :

- Dans tous les bras de l'étude, la couverture de traitement des enfants MAS selon le critère 1 a été plus élevée que la couverture de traitement des enfants MAS selon le critère 2. Simplement dit, dans tous les bras de l'étude, si un enfant est MAS par PB, il est plus probable qu'il ou elle sera couvert.e par le traitement qu'un enfant qui est MAS uniquement par P/T.
- Dans les Bras 1 et 2, pour les deux critères, la couverture de traitement est plus élevée en comparaison au bras Contrôle. Mais on peut constater que la couverture de traitement selon le critère 1 est plus élevée en comparaison au critère 2.
- Dans le Bras 2, puisque les dénominateurs pour les critères 1 et 2 sont assez petits, il n'est pas possible d'estimer la couverture avec une précision acceptable. Cependant, dans ce Bras, la couverture de traitement peut être classifiée comme « Elevée » pour le critère 1 et « Modérée » pour le critère 2.

Tableau 18: Estimations de couverture pour les cas MAM selon le Critère 1 (Par PB uniquement ou par PB+P/T)

	Cas trouvés		Dénominateur	Numérateur	Estimation de couverture	Intervalles de confiance	
	Cin	Cout	Cin+Cout	Cin	Numerator/ Denominator	Lower CI	Upper CI
Contrôle	20	53	73	20	27.4%	17.2%	37.6%
Bras 1	33	106	139	33	23.7%	16.7%	30.8%
Bras 2	17	47	64	17	26.6%	15.7%	37.4%

Tableau 19: Estimations de couverture pour les cas MAM selon le Critère 2 (Par P/T uniquement)

	Cas trouvés		Dénominateur	Numérateur	Estimation de couverture	Intervalles de confiance	
	Cin	Cout	Cin+Cout	Cin	Numerator/ Denominator	Lower CI	Upper CI
Contrôle	18	91	109	18	16.5%	9.5%	23.5%
Bras 1	25	131	156	25	16%	10.3%	21.8%
Bras 2	7	33	40	7	17.5%	5.7%	29.3%

Les tableaux 18 et 19 indiquent les estimations de couverture pour les cas MAM selon le Critère 1 (Par PB uniquement ou par PB+P/T) et le critère 2 (Par P/T uniquement).

Pour les cas MAM, globalement, les estimations de couverture de traitement pour la MAM dans les trois bras de l'étude indiquent que la couverture est plus élevée le critère 1 que pour le critère 2. Comme pour les résultats des cas MAS, il est plus probable qu'un enfant qui est MAM par PB serait couvert par le programme en comparaison à un enfant qui est MAM uniquement par le P/T. Cependant, il n'y a pas de différences significatives des estimations de couverture entre les trois bras.

1. Le dépistage précédent

Les figures 1 et 2 nous montrent que dans les bras Contrôle et Bras 2, moins de 40% des accompagnants des cas MAS et MAM (dans le programme et pas dans le programme) ont dit que leur enfant a été dépisté chez eux précédemment avant l'enquête. Ces informations indiquent que les relais communautaires n'ont pas réalisé un dépistage effectif de porte à porte dans la plupart des villages des aires de santé des deux bras

d'étude. Dans le Bras 1, 58% ont confirmé avoir effectué un dépistage précédemment chez eux avant l'enquête. Ce qui indique une mobilisation communautaire plus forte dans les aires de santé dans ce bras d'étude. Pour les accompagnants qui ont confirmé un dépistage

précédent dans le ménage, dans tous les bras de l'étude la plupart ont dit que le relais communautaire a effectué le dépistage de leur enfant pendant les 30 jours avant l'enquête de couverture. On peut donc conclure que dans beaucoup de villages des 3 bras d'étude, le dépistage porte à porte des enfants n'est pas effectif. Mais dans les villages où il est effectif, il y a un dépistage régulier.

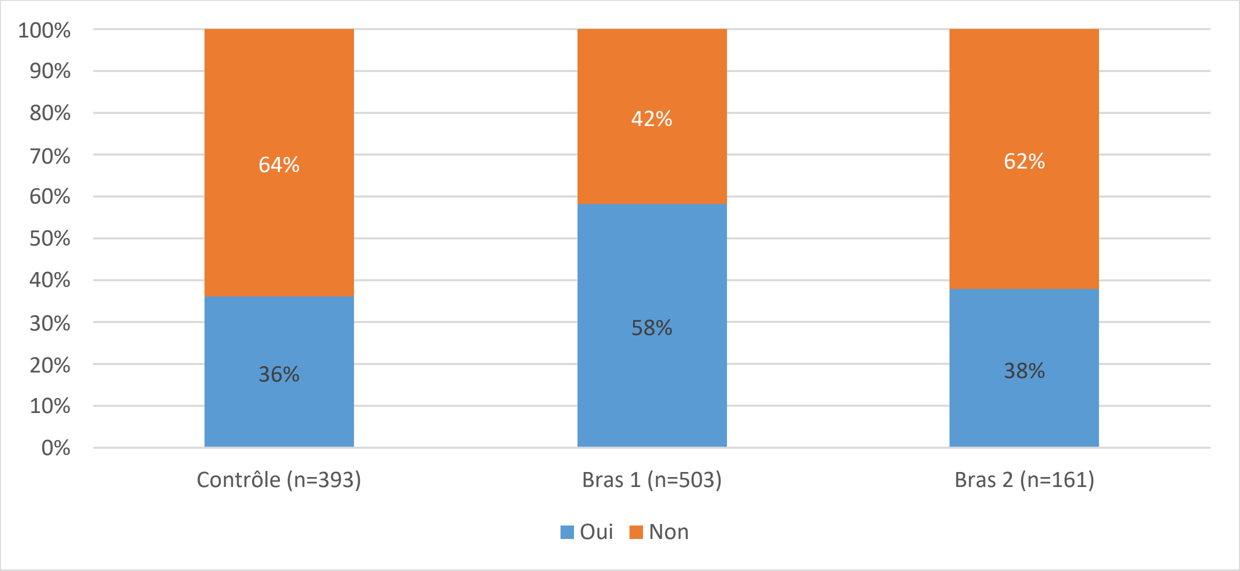

Figure 1: Réponse à la question: Votre enfant a-t-il été dépisté à la maison par un ruban PB avant ? (« n » est égal au nombre de répondants dans chaque bras de l'étude)

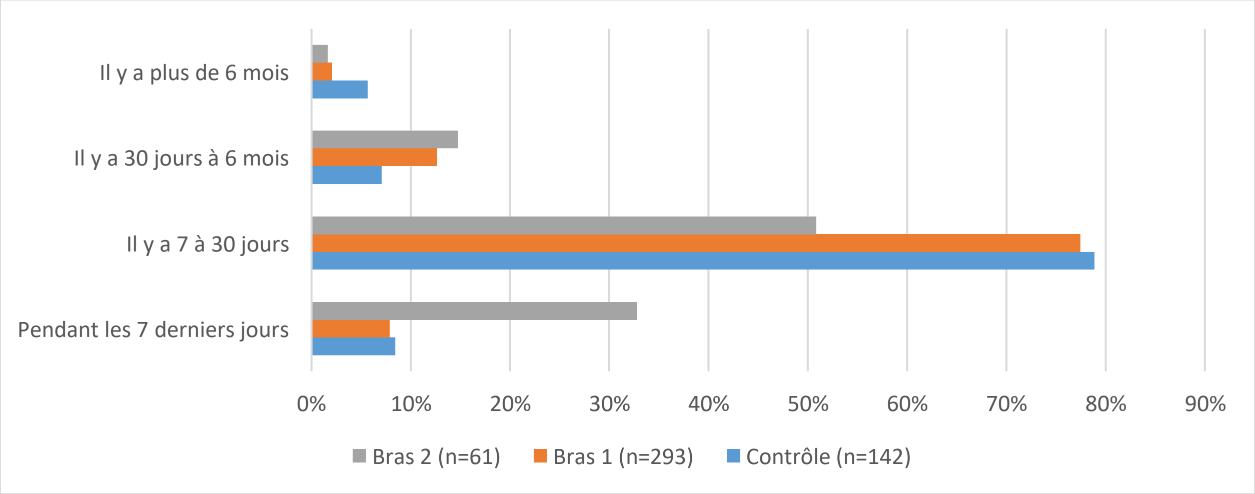

Figure 2: Pour les accompagnants qui ont confirmé que leur enfant a été dépisté par un PB précédemment, la période depuis de la mesure.

2. Analyse des questionnaires complétées avec les cas couverts et non-couverts

Cas couverts :

Les réponses des accompagnants des cas couverts par le programme de traitement PCIMA (les cas MAM et MAS) ont été analysées afin de déterminer la raison principale pour laquelle il/elle ont inscrit leur enfant dans le programme. Les résultats sont résumés dans les figures 3 à 5 :

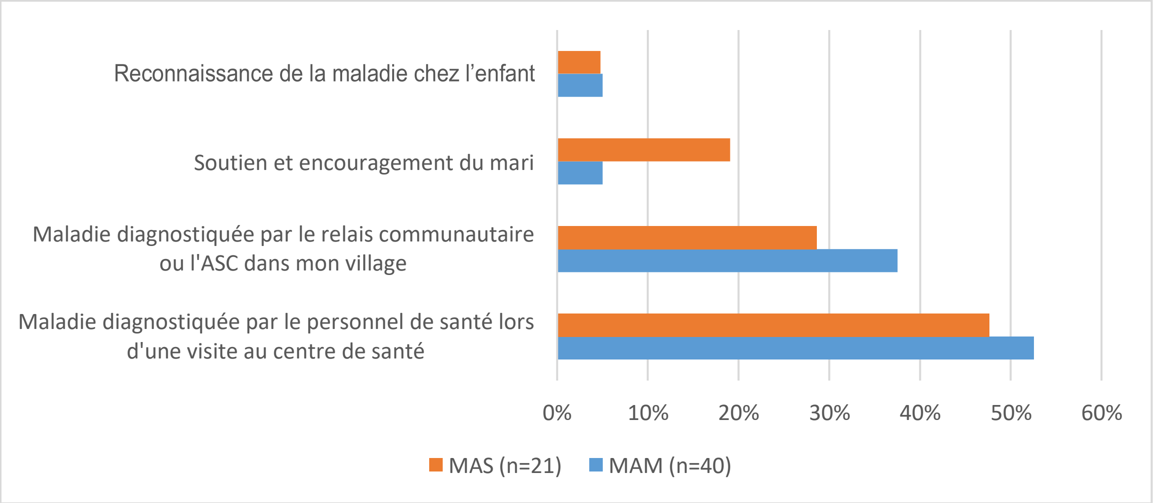

Figure 3: Zone contrôle : Les raisons principales pour lesquelles les accompagnants des cas MAM et MAS couverts ont inscrit leur enfant dans le programme PCIMA

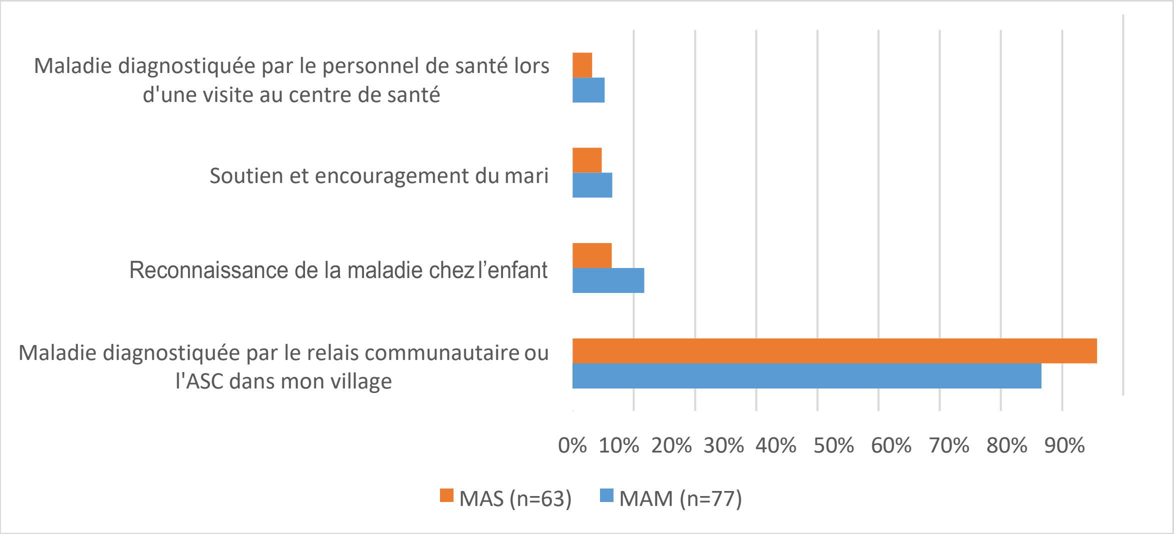

Figure 4: Bras 1: Les raisons principales pour lesquelles les accompagnants des cas MAM

et MAS couverts ont inscrit leur enfant dans le programme PCIMA.

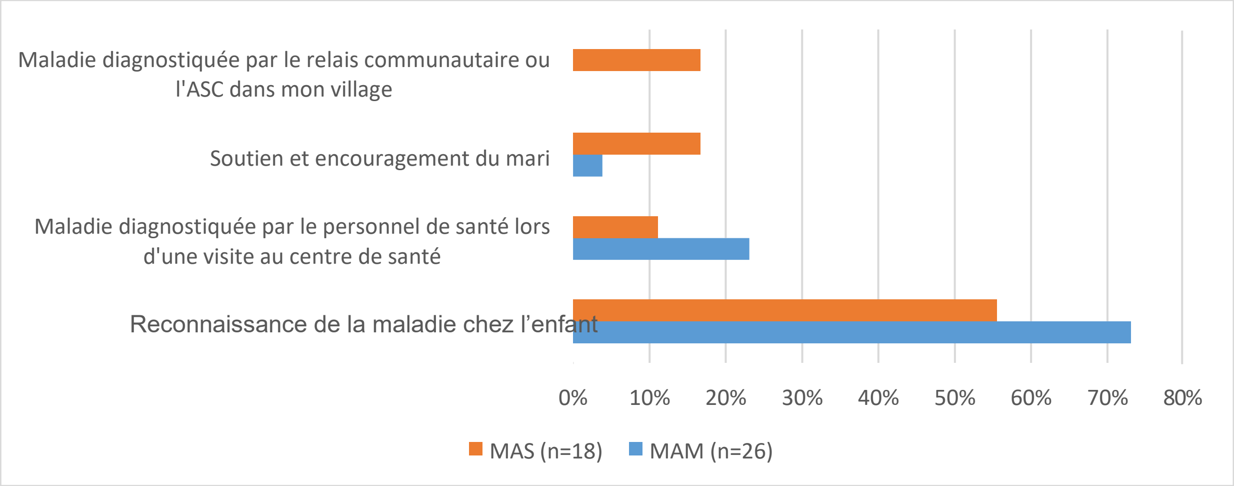

Figure 5: Les raisons principales pour lesquelles les accompagnants des cas MAM et MAS couverts ont inscrit leur enfant dans le programme PCIMA

Les données des figures 3 à 5 indiquent les comportements des accompagnants variant dans les trois zones de l'étude. Dans le bras Contrôle, on peut constater que, pour les cas qui ont été admis au programme, la plupart d'enfants malnutris ont été admis lors d'une visite au centre de santé (CSCOM). Ces informations suggèrent que le dépistage actif dans les centres de santé est assez fort et que le dépistage au niveau communautaire par les acteurs communautaires (relais) est plus faible (qui a été triangulé par les résultats dans le Figure 1). Ces données indiquent également que l'accès aux centres de santé est plus facile en comparaison avec les autres zones de l'étude.

Par contre, dans le Bras 1, où 68% des accompagnants ont confirmé que leur enfant avait été dépisté précédemment par un ASC chez eux, on peut constater que la plupart des cas couverts ont confirmé qu'ils ont amené leur enfant au centre de santé par suite d'un dépistage par un acteur communautaire (relais, ASC) chez eux.

Dans Bras 2, qui ont été confirmé d'avoir les couvertures de traitement les plus élevées parmi les trois zones de l'étude, la reconnaissance de la maladie chez l'enfant a été la raison la plus fréquente pour les cas MAM et MAS déjà enregistres dans le programme PCIMA. Il se peut que les ASC aient effectué beaucoup de sensibilisations dans les villages de cette zone qui pourrait expliquer ce résultat.

Cas non-couverts :

Les raisons principales pour lesquelles les accompagnants des cas MAM et MAS non-couverts n'ont pas amené leurs enfants au CSCOM ou au niveau du site ASC pour le traitement sont montrées dans les Figures 6, 7 et 8.

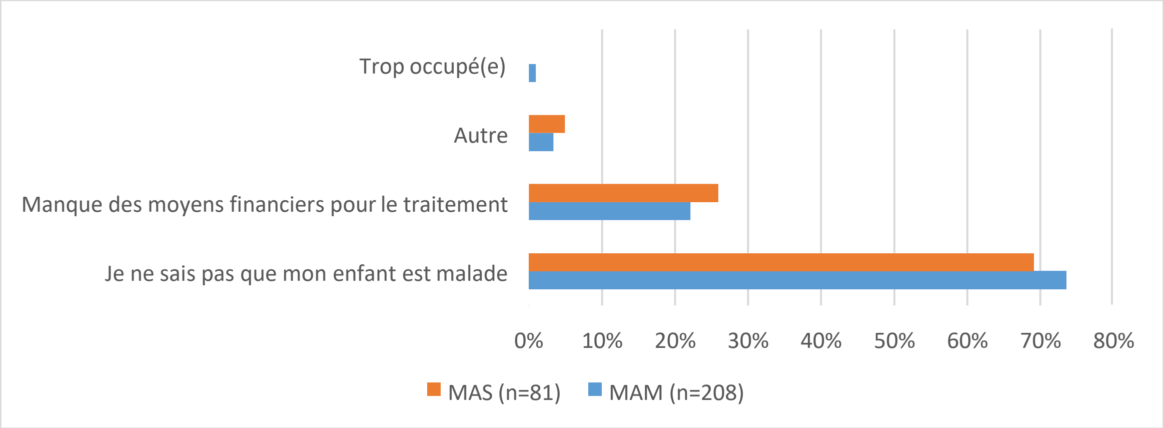

Figure 6: Zone contrôle : Les raisons principales pour lesquelles les accompagnants des cas MAM et MAS n'ont pas amené leur enfant au CSCOM

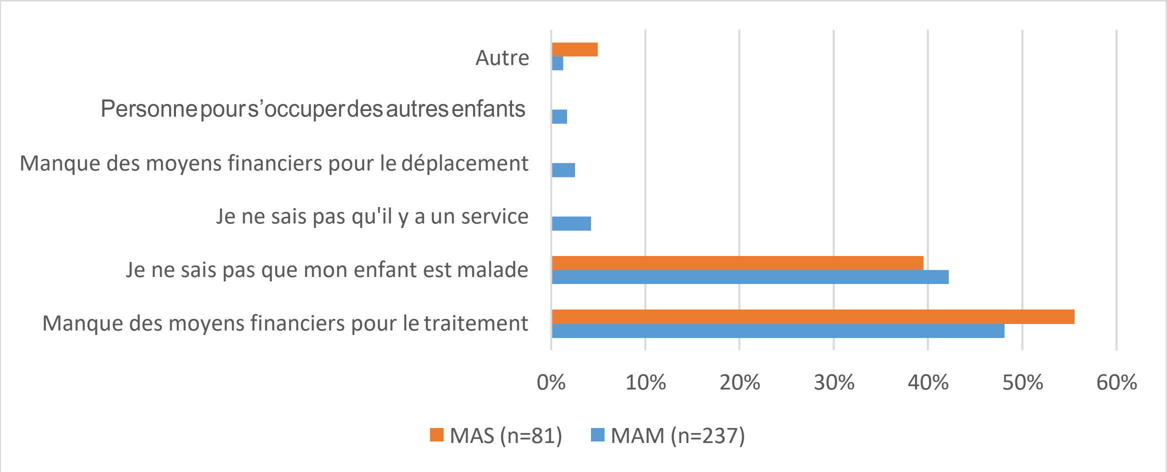

Figure 7: Bras 1: Les raisons principales pour lesquelles les accompagnants des cas MAM et MAS n'ont pas amené leur enfant au CSCOM et/ou site ASC

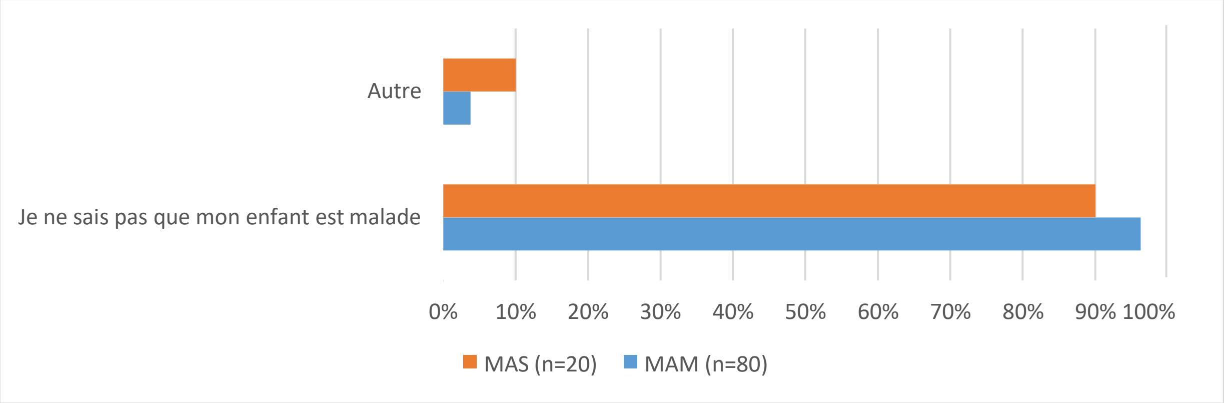

Figure 8: Bras 2: Les raisons principales pour lesquelles les accompagnants des cas MAM et MAS n'ont pas amené leur enfant au CSCOM et ou site ASC

Les résultats dans les Figures 7 à 9 résument les barrières au traitement selon les accompagnants des cas MAS et MAM qui n'ont pas été couverts dans le programme PCIMA des zones de l'étude.

Dans le bras Contrôle et dans le Bras 2, on peut constater que la première raison pour la non-couverture a été que l'accompagnant n'a pas connu que son enfant a été malade. Certes que, la moitié des enfants MAS et MAM identifiés pendant l'enquête ont été MAS et MAM basés uniquement sur leurs mesures de poids et taille, il est possible que la plupart des accompagnants n'ont pas pu reconnaitre les symptômes de MAS ou MAM de leur enfant.

Aussi, dans ces deux zones de l'étude, il semble qu'il n'y a pas un dépistage régulier par les acteurs communautaires (Figure 1). Et surtout dans le bras Contrôle, nous pouvons conclure que ce résultat est dû à un manque de mobilisation communautaire ou de dépistage porte à porte par les relais communautaire ou ASC. Cependant, dans le Bras 2, où nous avons constaté des estimations de couverture assez élevées, les résultats nous indiquent que là où il y a un dépistage régulier par les acteurs communautaires (relais ou ASC) la reconnaissance des symptômes de malnutrition est assez forte. Cependant, le dépistage et la mobilisation communautaire ne sont pas forte partout dans le Bras 2.

La première barrière dans le Bras 1 (et la deuxième dans le bras Contrôle) a été le manque de moyens financiers pour le traitement. Certes que le traitement pour la MAS et la MAM est gratuit (ou devrait être gratuite), il est surprenant que cette raison ait été identifiée comme la première barrière au traitement par la plupart des accompagnants. Ces informations peuvent sous-entendre que :

- Certains CSCOM sollicitent un paiement pour le traitement (vu que la gratuité des soins n'est pas effective dans tous les CSCOM),
- Certaines communautés croient que le traitement pour MAS ou MAM est payant.
- Manque de moyens financiers pour le transport du village au CSCOM surtout

dans le bras contrôle.

Quel qu'en soit la raison, il est essentiel que l'équipe d'Action Contre Faim mène une investigation sans délai afin de connaitre la ou les raison/s.

En plus, dans les questionnaires complétés avec les accompagnants, les enquêteurs ont pu enregistrer les commentaires additionnels concernant le programme de traitement pour le MAS et MAM. Dans ces commentaires, les ruptures de stock ont été souvent identifiées comme un facteur négatif du programme dans le Bras 1.

V. DISCUSSION

Le projet ICCM+ dans les 3 bras a pour but d'intégrer les traitements MAS au niveau communautaire (ASC) dans le bras 1 et le bras 2 à l'aide du protocole standard et modifié de la PCIMA. Cette enquête de couverture de mai 2021 visait à évaluer la couverture finale des traitements MAS et MAM chez les enfants âgés de 6 à 59 mois dans les bras d'intervention (bras 1 et bras 2) et le bras contrôle de l'étude dans le district sanitaire de Gao. Les résultats de l'évaluation de couverture aboutissent à une estimation la couverture qui est ainsi estimée :

	Bras contrôle				Bras 1				Bras 2			
	Mars-20		Mai-21		Mars-20		Mai-21		Mars-20		Mai-21	
	MAS	MAM	MAS	MAM	MAS	MAM	MAS	MAM	MAS	MAM	MAS	MAM
Couverture Unique	20,5%	9,8%	18.9%	16.1%	25,00	13,9%	38.5%	23.4%	6,3%	12,1%	48.8%	25.9%
Intervalle de Confiance	12,0%-28,9%	6,4%-13,2%	11.6%-26.2%	11.7%-20.5%	13,7%-36,3%	8,2%-19,5%	31.1%-45.8%	19% - 27.9%	1,4%-11,2%	6,8%-17,3%	33.5%-64.1%	17.9%-33.8%

Les résultats de l'enquête montrent une couverture unique des MAS, MAM en dessous de 50% qui est le seuil recommandé par les normes sphères en zone rurale. Les estimations de couverture du programme MAS indiquent une augmentation de la couverture dans les Bras 1 et Bras 2 comparativement à l'enquête Baseline de mars 2020. La couverture de traitement de la MAS dans le bras « Contrôle » n'a pas évoluée pendant la mise en place l'étude. Mais les couvertures du traitement de la MAS dans Bras 1 et 2 peuvent être classifie comme « Modérée » (entre 20 et 50%). Les estimations de couverture du programme MAM montrent également une augmentation de la couverture dans les bras d'intervention (Bras 1 et Bras 2) comparativement à l'enquête Baseline de mars 2020 mais

nous n'avons eu aucun changement dans le bras Contrôle entre l'enquête Baseline et End-line. Les estimations de couverture pour les Bras 1 et 2 se trouvent dans la classification « modérée » parce qu'elles dépassent les 20%. Mais dans ces bras de l'étude, seulement un quart des enfants MAM ont été couverts par le traitement.

Dans les bras Contrôle et Bras 2, moins de 40% des accompagnant(e)s des cas de MAS et de MAM (dans le programme ou non) ont dit que leur enfant a été dépisté chez eux avant l'enquête. Ces informations indiquent que dans certains villages de ces zones d'étude, les activités communautaires sont limitées c'est-à-dire que les relais communautaires ne réalisent pas un dépistage actif (dépistage porte-à- porte).

Dans le bras Contrôle, les estimations de couverture du traitement pour la MAS et MAM basses (18.9% et 16.1% respectivement) démontrent l'impact de la faible mobilisation communautaire. En plus, la plupart des accompagnants des cas non-couverts ne reconnaissent pas les symptômes de la malnutrition de leurs enfants ce qui ajoute à l'évidence que les acteurs communautaires n'effectuent pas une sensibilisation effective aux accompagnants dans ces communautés.

La couverture de traitement a été plus élevée dans les Bras 1 et 2, avec les estimations de couverture de 38.5% et 48.8% pour le traitement MAS respectivement. Ces résultats suggèrent que si le traitement est administré par les ASC dans la communauté, on attendrait une couverture de traitement plus élevée en comparaison avec un traitement administré dans les CSCOM. Cependant, parmi les cas non-couverts identifies dans Bras 2, les raisons pour la non-couverture ont été semblables que dans le bras Contrôle. Ceci suggère qu'il y a certaines communautés dans le Bras 2 qui ne sont pas bien soutenues par les acteurs communautaires.

La différence entre les estimations dans Bras 1 et Bras 2 suggèrent également que le protocole simplifié mené dans Bras 2 entrainerait une couverture plus élevée en comparaison avec le protocole standard. On peut avancer l'hypothèse que, pour les ASC, le protocole simplifié est plus facile à mettre en place que le protocole standard. Mais dans cette enquête, a l'exception des estimations de couverture légèrement différentes dans les deux Bras de l'étude, il n'existe pas des évidences à soutenir cette hypothèse.

Dans le Bras 1, 58% des accompagnant(e)s ont confirmé que leur enfant a été dépisté dans le ménage ce qui indique une mobilisation communautaire plus forte dans les aires de sante de ce bras d'étude. Par contre, la plupart des accompagnants des cas non-couverts identifies dans Bras 1 ont dit qu'il/elle n'a pas amené son enfant au CSCOM pour le traitement a cause du cout de traitement ou d'une rupture de stock. Ces informations suggèrent qu'il y a des faiblesses liées au fonctionnement du programme PCIMA et que, dans certaines communautés, il y a une perception que le service est payant.

VI. CONCLUSION

En conclusion, basée sur les résultats de l'enquête de couverture end line dans le DS de Gao, nous pouvons constater une augmentation de la couverture de traitement dans les deux bras d'intervention Bras 1 et 2. La plus grande augmentation a été observée dans le Bras 2, ou un protocole simplifié pour le traitement de MAS et MAM a été mise en place dans les communautés par les ASC ainsi que dans les CSCom. Dans le Bras 1, où les ASC ont mis en place le protocole PCIMA standard au niveau communautaire ainsi de même dans les CSCom par les agents de santé qualifiés, la couverture de traitement dans ce bras a également augmenté surtout pour les enfants MAS. Cependant, il semble que les ruptures de stock, et les perceptions que le service soit payant, ont limité l'augmentation de la couverture.

Basé sur les informations dans ce rapport, il est difficile de conclure quel aspect du protocole suivi a conduit à l'augmentation de la couverture dans le Bras 2. Avec les ressources limitées pour cette enquête, il n'était pas possible d'inclure dans la méthodologie une collecte des données qualitatives et une analyse des données de programme qui pourraient aider à expliquer la couverture élevée.

Donc il est conseillé de mener une analyse de toutes les données (par ex. les données du programme, les données qualitatives collectées pendant les recherches qualitatives menées pendant et après l'étude dans la communauté et les résultats de l'enquête de couverture endline) afin de présenter les évidences triangulées concernant l'efficacité du traitement de MAS et MAM par les ASC avec le protocole modifi
